# Minorities’ Diminished Psychedelic Returns: Income and Educations Impact on Whites, Blacks, Hispanics, and Asians

**DOI:** 10.1007/s40615-024-02023-y

**Published:** 2024-05-16

**Authors:** Sean Matthew Vina

**Affiliations:** Department of Sociology, The University of the Incarnate Word, 4301 Broadway, San Antonio, TX 78209 USA

**Keywords:** Psychedelic, Race, Ethnicity, Mental health, Inequality

## Abstract

Growing evidence suggests that the race and ethnic minority population may experience fewer protective effects of psychedelics on mental health. The minority diminished psychedelic returns theory proposes that racism, manifested in socioeconomic inequality, could partially account for the smaller health gains observed. Therefore, it is important to investigate whether socioeconomic inequality reduces the impact of psychedelics on health outcomes for minority populations. Additionally, despite having higher socioeconomic status, it remains unclear whether psychedelic use among minorities is associated with the same level of health benefits as observed in non-Hispanic whites. This study utilizes data from the National Survey of Drug Use (*N* = 2008 to 2019), which involved 458,372 participants aged 18 and above. The objective is to examine the impact of various psychedelics (MDMA, psilocybin, DMT, ayahuasca, peyote/mescaline, and LSD), as well as lifetime classic psychedelics use (LCPU), on psychological distress in the past month, taking into account socioeconomic factors (education level and family income) and race/ethnic differences (White, Black, Hispanic, and Asian). The analysis employed a series of nested ordinary least-square regression models using Stata 18. The results indicate that, after controlling for socioeconomic status, there is no association between Black and Hispanic psychedelic use and distress. However, white psychedelic use remains associated with lower levels of distress. Additionally, despite having higher levels of education and income, psychedelic use among minority groups does not appear to be linked to reduced stress. In fact, for Asians with higher education and income, certain psychedelic use is associated with increased distress.

**Supplementary Information:**

The online version contains supplementary material available at 10.1007/s40615-024-02023-y.

## Introduction

Despite ample research indicating a strong positive correlation between psychedelics and various health outcomes such as depression, anxiety, suicidality, post-traumatic stress disorder (PTSD), drug dependency, and behavioral addictions [19, 25, 31–33, 38, 50, 55, 64, 68, 70, 71, 73, 74, 91, 93], emerging evidence suggests that the protective effects of psychedelics vary among different subpopulations, with minority populations experiencing fewer benefits. For instance, national data has shown that while psilocybin and MDMA (3,4-methylenedioxymethamphetamineuse) use improved health outcomes for white users, the same effects were minimal or nonexistent among users from racial or ethnic minority groups [36, 38]. Another study found that employed psychedelic users had significantly lower levels of psychological distress compared to non-employed users, who exhibited higher levels of distress [88]. Additionally, research has demonstrated that Black psychedelic users did not experience a decrease in stress, unlike their white counterparts whose stress levels significantly decreased, particularly among those with higher levels of education [87]. Moreover, the study revealed that white men seemed to derive the most benefits from psychedelics, and that criminal history weakened the positive association between psychedelics and health outcomes for white users, but not for Black users. It is also worth noting that social factors, such as integration, may moderate these effects [85], as supported by other research that found larger household sizes can negatively impact the effectiveness of psychedelics in reducing distress [86].

The minorities’ diminished psychedelic returns theory (MDPR) proposes an explanation for the smaller health benefits of psychedelic use among minority populations. This theory utilizes a modified version of the cultural set-and-setting theory and takes a social-epidemiological approach [87]. By incorporating structural inequalities into the framework, the theory suggests that these inequalities can negatively impact the effects of psychedelics on health, not only during use but also before and after [87]. While initial research focused on how cultural conditions can influence perceptions of psychedelics during use [11, 30], the modified theory proposes that structural inequalities can also affect access to resources and exposure to trauma/stress after psychedelic use, leading to a quicker diminishing of the drugs’ efficacy [87]. Importantly, MDPR suggests that systemic racism plays a significant role in the entire relationship individuals have with psychedelics, both before and after use. Systemic racism affects how minorities perceive, access, and relate to psychedelics, potentially altering their effectiveness in promoting mental health. Additionally, structural racism can impact the relationship between psychedelics and health post-use due to unequal access to resources that could influence the drugs’ efficacy or exposure to trauma that may diminish their effectiveness more rapidly [87]. In summary, MDPR proposes that, all things being equal, minorities experience suboptimal set-and-setting conditions and have fewer necessary conditions for optimal or sustained health outcomes associated with psychedelic use.

Although previous research has found that minorities benefit less from psychedelics, this paper aims to test whether socioeconomic inequality contributes to these disparate outcomes. The study addresses two research questions: does socioeconomic inequality diminish the impact of psychedelics on health outcomes for minority populations, and despite having higher socioeconomic status, do minorities experience the same health benefits from psychedelic use as non-Hispanic whites? By examining race/ethnic differences in the relationship between psychedelics, socioeconomic status, and distress, this study takes an intersectional approach to bridge the gap between sociomedical scholarships and psychedelic studies. It builds upon a modified theory of cultural set and setting and draws upon a robust history of the stress process to analyze health pathways across social structures [62, 63]. The study predicts several relationships between these variables and psychological distress, highlighting potential relationships that are conceptually distinct but not mutually exclusive. Specifically, the study assesses four empirical predictions regarding the relationship between race/ethnicity, socioeconomic status, health, and psychedelics. Firstly, (1) although psychedelic use will be independently associated with lower levels of distress, controlling for socioeconomic status will weaken this association. Secondly, (2) the negative impact of socioeconomic inequality on the positive association between psychedelics and distress will be more pronounced for racial/ethnic minorities compared to non-Hispanic whites. Thirdly, (3) the positive association between psychedelics and health will be weaker for individuals with lower socioeconomic status. Finally, even with high levels of education and income, there will be no association between psychedelics and distress for racial/ethnic minorities.

The goal of this analysis is to understand if socioeconomic inequality affects psychedelics returns for minority populations. To test the predications on the relationship between race/ethnicity, socioeconomic status, psychedelics, and distress, this paper draws from the National Survey of Drug Use (*N* = 2008 to 2019) which included 458,372 participants aged 18 or older. The study examines the impact of various psychedelics (such as MDMA, psilocybin, DMT (N,N-dimethyltryptamine), ayahuasca, peyote/mescaline, and LSD (lysergic acid diethylamide)) and one measure of lifetime classic psychedelics use (LCPU) psychological distress in the past month. A series of nested ordinary least-square regression models were used in the analysis, conducted using Stata 18. In addition, to analyze race/ethnic differences, the study employs an intersectional approach by running the analysis by non-Hispanic whites, non-Hispanic African Americans, Hispanics, and non-Hispanic Asians.

## Data and Methods

This present study utilized pooled, cross-sectional data from the National Survey of Drug Use and Health (NSDUH) conducted from 2008 to 2019 (*N* = 458,372). The NSDUH is an annual survey conducted in all 50 states and the District of Columbia. Its purpose is to measure the prevalence of substance use and mental health issues in the United States. The data was weighted to accurately represent the civilian noninstitutionalized population. Table 1 provides descriptive statistics for the dependent, independent, and control variables, all of which are derived from publicly available data. The NSDUH public-use data files can be accessed on their homepage: https://www.datafiles.samhsa.gov/dataset/nsduh-2002-2019-ds0001-nsduh-2002-2019-ds0001.

**Table 1:** Descriptive statistics for dependent variables, independent variables, and controls (2005–2019) (weighted)

	Mean	SD	*n*	%/ min–max
Dependent variables				
Psychological distress in past 30 days	9.52	6.06	151,683	0–24
Key independent variables				
Lifetime psychedelic use				
MDMA			32,468	7.09
Psilocybin			43,294	9.45
DMT			370	.08|
Ayahuasca			41	.01
Peyote/mescaline			19,997	4.36
LSD			49,720	11.80
LCPU			63,893	13.94
Race/ethnicity			48,413	10.56
White			301,989	65.88
Black			53,430	11.66
Hispanic			68,640	14.97
Asian			23,563	5.14
Education	2.77	1.02	458,372	1–4
Family income	4.97	2.02	458,372	1–7
Control variables				
Race				
Hawaiian			1635	0.36
Native American			2397	0.52
Other (multi-racial)			6715	1.46
Women			237,730	51.86
Age	14.82	2.06	458,372	8–17
Marital status				
Single, never married			120,128	26.21
Married			245,431	53.54
Widowed			27,879	6.08
Divorced/separated			64,932	14.17
Religious attendance	1.89	1.88	454,781	0–5
Religiosity	4.93	2.60	446,968	0–9
Lifetime drug use				
Cocaine			75,007	16.36
Marijuana			213,442	46.57
PCP			12,144	2.65
Inhalants			40,656	8.88
Stimulants			46,617	10.17
Sedatives			38,369	8.37
Tranquilizer			76,047	16.59
Heroine			9121	1.99
Pain relievers			165,821	36.18
Tobacco			266,963	63.34
Age of first alcohol use	2.87	1.15	458,372	1–5
Self-reported risky behavior	1.60	0.74	456,796	0–3

Source: 2008–2019 National Survey of Drug Use and Health, *n* = *458,372*

The NSDUH was initially conducted with approval from the internal review board of the Substance Abuse and Mental Health Services Administration. Consent from participants was obtained in accordance with the guidelines set by the IRB. To ensure complete confidentiality, all identifying information was removed from the publicly available data. As this study utilizes anonymous and publicly available data, there was no need to seek ethics approval or obtain consent.

### Study Replications

The present study aims to replicate previous research that has analyzed the association between psychedelic use and health outcomes, using data from the National Survey on Drug Use and Health (NSDUH) [32, 33, 37, 44, 45, 52, 72, 71, 73, 74, 85, 87, 88]. While this paper replicates the study that analyzes the association between psychedelics and mental health [33, 37, 71, 73, 74], there are almost a dozen other papers that use an established statistical procedure for analyzing psychedelics and outcomes using the NSDUH, which this paper follows closely. This study uses the same dependent, independent, and control variables for regression analyses.

### Dependent Variables

This analysis includes one measure of level of distress in the past month using the Kessler Psychological Distress Scale (K6) [40, 41], which was provided by the NSDUH and was used in previous research on psychedelics and health [33, 37, 71, 73, 74]. Participants indicate how often they have had six different feelings or experiences during the past 30 days using a 5-point Likert scale: 4 (all of the time), 3 (most of the time), 2 (some of the time), 1 (a little of the time), and 0 (none of the time). The feelings and experiences for this first item are the following: “nervous,” “hopeless,” “restless or fidgety,” “so depressed that nothing could cheer you up,” “that everything was an effort,” and “worthless.” Psychological distress in the past month is created by adding all measures into one scale that ranges from 0 to 24, with higher scores indicating more distress. The Kessler scale of psychological distress is a well-established, reliable, and valid measure of psychological distress in people (adults and adolescents across genders) with panic disorder, generalized anxiety disorder, bipolar disorder, and schizophrenia [18, 66, 82].

### Dependent Variables: Psychedelics

This analysis includes seven key measures of drug use. Respondents were asked if they had ever used the following drugs, even once: MDMA, DMT, ayahuasca, psilocybin, LSD, and mescaline peyote. Six of these measures are classic psychedelics, a subclass of psychedelics that have little toxicity [20], and promote neurogenesis [50, 57]. These classic psychedelics include N-dimethyltryptamine (DMT), the DMT-containing admixture ayahuasca, psilocybin, lysergic acid diethylamide (LSD), mescaline, and the mescaline-containing cacti peyote. Consistent with previous [71, 73, 74], the six classic psychedelics were used to create a dummy variable indicating any lifetime classic psychedelic use (LCPU) (yes vs. no).^1^

On the other hand, MDMA induces a “flood of serotonin in the brain” and other cascading effects which is also associated with a host of better mental health outcomes in both clinical trials and population studies [10, 14, 37, 54, 55, 89, 90]. For instance, one nationally represented study found MDMA was associated with lower suicide risk (ideation and planning) less psychological distress [37].

Additionally, while the paper replicates previous research and creates a single measure of lifetime classic psychedelic use (LCPU), it also analyzes each drug independently. Although grouping the classic psychedelics into one measure of LCPU is valid because of their similar properties, as conducted in previous population studies [33, 44, 72, 71, 71, 73, 73, 74, 74], it is important to note that the ways in which people use these drugs may vary when measuring naturalistic use versus carefully controlled clinical settings. There is ample evidence that certain psychedelics are used in different situations. For example, MDMA, psilocybin, and LSD are some of the most widely available drugs on the illicit marketplace [35]. Furthermore, MDMA, LSD, and psilocybin are more commonly used for recreational purposes by young people [15, 61, 67]. In contrast, ayahuasca, peyote, and mescaline are predominantly used within specific communities, such as indigenous and religious retreats [21, 39]. These drugs are more likely to be consumed in naturalistic settings for spiritual healing. By testing LCPU and individual drugs, this analysis considers those different uses and cultural distinctions. Mescaline and peyote were grouped together as one drug due to their shared origin and the fact that they are consumed by similar groups in similar social situations [27, 39].

### Socioeconomic Status and Race/Ethnicity Independent Variables

Socioeconomic class is measured by two continuous variables: (1) annual household income (less than US $10,000, US $10,000–US $19,999, US $20,000–US $29,999, US $30,000–US $39,999, US $40,000–US $49,999, US $50,000–US $74,999, and US $75,000 or more) and (2) educational attainment (high school degree, some college, college degree or higher, and less than a high school degree, serving as the reference category). The analysis followed recommendation to test both variables as continuous and categorial variables throughout the analysis [48], but results proved substantially identical, so they were left as continuous variables to facilitate interpretations. There are four relevant dummy variables for race/ethnicity for this analysis: (1) non-Hispanic African Americans, (2) Hispanics, and (3) non-Hispanic Asians, with (4) non-Hispanic whites serving as the reference category. Three other race/ethnicity dummy variables were included in the analysis as controls variables: non-Hispanic Native American/Alaska Native, non-Hispanic Native Hawaiian/Pacific Islander, and non-Hispanic more than one race.^2^

### Control Variables

The analysis includes several sociodemographic controls. Women is a binary variable with “men” set as the reference category. Age is a continuous measure (18, 19, 20, 21, 22–23, 24–25, 25–29, 30–34, 35–49, 50–64, and 65 +) and marital status is a categorical variable (married, widowed, divorces/separated, and single-never married serving as the reference category). The regression analysis also controls for the year of the survey, based on previous research using the NSDUH. Previous research has found a strong correlation between drug use, risky behavior, and mental illness. Therefore, this study follows the guidance of similar research that uses the NSDUH by including controls for other drug use [32, 33, 37, 44, 45, 52, 72, 71, 73, 74, 85, 87, 88]. It includes the following binary control variables: lifetime use of cocaine, marijuana use, phencyclidine (PCP), inhalants, other stimulants, sedatives, pain relievers, and tobacco use (i.e., smokeless tobacco, pipe tobacco, cigar, and daily cigarette use). Age of first alcohol use and self-reported engagement in risky behavior are continuous variables. Finally, there are two religiosity variables. First, religious attendance measures how often a person attended religious services in the last year with the following option: (0 =) 0 ties, (1 =) 1 to 2 times, (2 =) 3–5 times, (3 =) 6 to 24 times, (4 =) 25 to 52 times, and (5 =) more than 52 times. Religious salience is an index of the following three variables: (1) my religious beliefs are very important, (2) my religious beliefs influence life, and (3) it is important that my friends are religious (Cronbach’s *a* = 0.83). Table 1 shows the descriptive statistics for all variables.

### Analytic Strategy

To address study’s questions, the study began by calculating the mean of each variable in the sample by race. Then, the analyses conducted a post-estimation LINCOM (nonlinear combination) commands, which computes the statistical difference of two subpopulation means [49]. Statistical mean differences were calculated of, for instance, white People minus ( −) all non-white people and or Black people minus ( −) all non-Black people. Next, a series of ordinary least-square regression (OLS) models were used to test the relationship between race/ethnicity, psychedelics, educational level, family income, and psychological distress with all control variables (Supplemental Tables 1–5).^3^ Model 1 examined the relationship between MDMA and LCPU on psychological distress. Model 2 add education and family income. Model 3 examines the relationships between each individual psychedelic variable (MDMA, psilocybin, DMT, peyote/mescaline, and LSD) on psychological distress. Model 4 adds education and family income. Models 5–11 test the interaction between each psychedelic and educational level on psychological distress. Models 12–18 test the interaction between each psychedelic and family income on psychological distress.

Using the intersectional approach that understands different groups have substantially different lived experiences (i.e., whites vs Blacks), the analysis follows recommendations to run individual models by race/ethnicity to compare substantially meaningful profiles [48]. Supplemental Table 1 displays the models for the full population. Supplemental Table 2 displays the models for non-Hispanic whites. Supplemental Table 3 displays the models for non-Hispanic Blacks. Supplemental Table 4 displays the models for Hispanics. Supplemental Table 5 displays the models for non-Hispanic, Asians. To facilitate the analysis of race/ethnicity differences across regression models, the analysis assessed the statistical difference of coefficients by running post-estimation SUEST (seemingly unrelated estimation) commands, which combine regression estimation results and allow for generalized Hausman specification tests [58]. The post-estimation commands are appropriate when the estimates were obtained on the same or overlapping data. NSDUH created weights by adjusting the single-year weights by a scalar factor (i.e., the number of years of data used) so that the estimated number of individuals reported is representative of the national population. All analyses incorporate the sampling weights provided by the NSDUH and conducted in STATA 18.

Finally, as with previous research on psychedelics using the NSDUH, there was no control for multiple comparisons in the present study [52, 71, 73, 74]. However, according to Armstrong [4], a Bonferroni correction is not needed for this study because the paper meets the following requirements: (1) it does not require a single test of the universal null hypothesis, (2) it does not need to avoid a type I error, and (3) it uses extensive preplanned hypothesis that drives this analysis.

## Results

### Descriptive Statistics

Mean differences by race (Table 2) reveal that white people have higher rates of psychological distress, income, and education compared to non-whites (*p* < 0.001). Additionally, white individuals are more likely to use any psychedelics, except for ayahuasca (*p* < 0.001). On the other hand, Black individuals (Table 2) have lower levels of psychological distress, education, and income compared to all other racial groups. Furthermore, they are less likely to use any psychedelics (*p* < 0.001). Hispanics, when compared to non-Hispanics (Table 3), have higher levels of psychological distress, lower education and income, and a lower likelihood of using any psychedelics, except for ayahuasca (*p* < 0.001). Lastly, Asians, in comparison to non-Asians (Table 3), have lower levels of psychological distress, higher education and income, and a lower likelihood of using any psychedelics, except for ayahuasca (*p* < 0.001).

**Table 2:** Mean difference of dependent, independent, and control variables by White and Black people (weighted)

	Mean		Mean difference^a^		Mean		Mean difference^a^	
	Non-White	White			Non-Black	Black		
Dependent variables								
Psychological distress in past 30 days	9.66	9.45	0.20	***	9.54	9.27	0.27	***
	(.045)	(.031)	(.052)		(.028)	(.064)	(.070)	
Independent variables								
Psychedelics								
MDMA	.05	.07	− .02	***	.07	.04	.02	***
Psilocybin	.04	0.12	− .07	***	0.10	.01	.08	***
DMT	.00	.01	− .01	***	.01	.00	.01	***
Ayahuasca	.00	.00	00		.01	.00	.01	***
Peyote/mescaline	.02	.05	− .03	***	.04	.01	.03	***
LSD	.05	0.13	− .08	***	0.11	.03	.08	***
LCPU	.07	0.17	− 0.10	***	0.15	.04	0.10	***
Educational level	2.53	2.89	− 0.35	***	2.85	2.52	0.27	***
	(.005)	(.003)	(.006)		(.003)	(.007)	(.008)	
Family income	4.43	5.25	− 0.81	***	5.08	4.09	0.98	***
	(.011)	(.008)	(.012)		(.008)	(.018)	(.019)	
Controls								
Women	0.52	0.51	.01	**	0.51	0.55	− .03	***
Age	14.42	15.02	− 0.66	***	14.85	14.57	0.28	***
	(.009)	(.006)	(.010)		(.006)	(.012)	(.012)	
Marital status								
Single, never married	0.34	0.21	0.13	***	0.24	0.42	− 0.18	***
Married	0.45	0.57	− 0.11	***	0.56	0.33	0.22	***
Widowed	.04	.06	− .02	***	.06	.06	− .00	
Divorced/separated	0.14	0.14	.00		0.13	0.17	− .04	***
Religious attendance	1.95	1.86	.08	***	1.84	2.28	− 0.44	***
	(.008)	(.006)	(.010)		(.005)	(.014)	(.015)	
Religiosity	5.34	4.72	0.62	***	4.81	5.86	− 1.05	***
	(.011)	(.007)	(.013)		(.007)	(.018)	(.019)	
Lifetime drug use								
Cocaine	0.11	0.18	− .07	***	0.17	0.10	.06	***
Marijuana	0.37	0.51	− 0.14	***	0.46	0.44	.01	***
PCP	.01	.03	− 0.01	***	.02	.02	.00	
Inhalants	.05	0.10	− .05	***	.09	.03	.06	***
Stimulants	.05	0.12	− .07	***	0.10	.04	.06	***
Sedatives	.04	0.10	− .05	***	.08	.04	.04	***
Tranquilizer	0.10	0.20	− .09	***	0.17	.09	.08	***
Heroine	.01	.02	− .01	***	.02	.01	.01	*
Pain relievers	0.28	0.40	− 0.11	***	0.37	0.29	.07	***
Tobacco	0.42	0.65	− 0.23	***	0.59	0.46	0.12	***
Age of first alcohol use	3.22	2.68	0.53	***	2.82	3.22	− 0.40	***
	(.005)	(.003)	(.006)		(.002)	(.008)	(.008)	
Self-reported risky behavior	1.45	1.68	− 0.27	***	1.63	1.41	0.22	***
	(.002)	(.003)	(.003)		(.001)	(.004)	(.004)	

Source: 2008–2019 National Survey of Drug Use and Health, *n* = *458,372*. ^a^Calculate using post-estimation LINCOM (nonlinear combination) commands, which compute the statistical difference of two subpopulation means. ^b^Standard deviations in parentheses. †*p* < 0.10, *p < 0.05, **p < 0.01, ***p < 0.001 (two-tailed)

**Table 3:** Mean difference of dependent, independent, and control variables by White and Black people (weighted)

	Mean		Mean difference^a^		Mean		Mean difference^a^	
	Non-Hispanic	Hispanic			Non-Asian	Asian		
Dependent variables								
Psychological distress in past 30 days	9.45	9.96	− 0.50	***	9.54	9.05	0.48	***
	(.028)	(.069)	(.073)		(.026)	(0.110)	(0.110)	
Independent variables								
Psychedelics								
MDMA	.07	.05	.01	***	.07	.04	.02	***
Psilocybin	0.10	.05	.04	***	.09	.02	.06	***
DMT	.01	.00	.00	***	.01	.00	.01	***
Ayahuasca	.00	.00	.00		.00	.00	.00	
Peyote/mescaline	.04	.02	.02	***	.04	.00	.03	***
LSD	0.11	.05	.05	***	0.10	.02	.08	***
LCPU	0.14	.08	.06	***	0.14	.04	0.10	***
Educational level	2.86	2.24	0.61	***	2.74	3.38	− 0.63	***
	(.003)	(.007)	(.007)		(.003)	(.012)	(.013)	
Family income	5.08	4.34	0.73	***	4.94	5.49	− 0.54	***
	(.008)	(.016)	(.016)		(.008)	(.028)	(.028)	
Controls								
Women	0.52	0.59	.02	***	0.51	0.53	− .01	***
Age	14.92	14.26	0.65	***	14.84	14.53	0.30	***
	(.005)	(.014)	(.015)		(.005)	(.021)	(.022)	
Marital status								
Single, never married	0.25	0.32	− .07	***	0.26	0.25	.00	
Married	0.54	0.50	.04	***	0.52	0.63	− 0.10	***
Widowed	.06	.03	.03	***	.06	.03	.02	***
Divorced/separated	0.14	0.13	.01	**	0.14	.06	.07	***
Religious attendance	1.91	1.78	0.12	***	1.89	1.82	.07	**
	(.005)	(.012)	(.013)		(.005)	(.024)	(.024)	
Religiosity	4.90	5.10	− 0.19	***	4.92	5.07	− 0.14	***
	(.007)	(.017)	(.018)		(.006)	(.033)	(.035)	
Lifetime drug use								
Cocaine	0.16	0.13	.03	***	0.17	.04	0.12	***
Marijuana	0.48	0.33	0.14	***	0.48	0.19	0.28	***
PCP	.02	.01	.01	***	.02	.00	.02	***
Inhalants	.09	.06	.03	***	.09	.03	.05	***
Stimulants	0.10	.06	.04	***	0.10	.03	.06	***
Sedatives	.09	.04	.04	***	.08	.03	.05	***
Tranquilizer	0.17	0.10	.06	***	0.17	.05	0.11	***
Heroine	.02	.01	.01	***	.02	.00	.01	***
Pain relievers	0.37	0.27	.09	***	0.36	0.21	0.15	***
Tobacco	0.60	0.40	0.19	***	0.59	0.27	0.31	***
Age of first alcohol use	2.82	3.13	− 0.30	***	2.82	3.67	− 0.85	***
	(.003)	(.007)	(.008)		(.002)	(.011)	(.011)	
Self-reported risky behavior	1.63	1.45	0.18	***	1.61	1.44	0.16	***
	(.001)	(.003)	(.004)		(.001)	(.008)	(.008)	

Source: 2008–2019 National Survey of Drug Use and Health, *n* = *458,372*. ^a^Calculate using post-estimation LINCOM (nonlinear combination) commands, which compute the statistical difference of two subpopulation means. ^b^Standard deviations in parentheses. †*p* < 0.10, *p < 0.05, **p < 0.01, ***p < 0.001 (two-tailed)

### Does Socioeconomic Status Affect Psychedelic Returns

Among the total population (Supplemental Table 1), LCPU is associated with less psychological distress (Model 1, *b* =  − 0.185, *p* < 0.01), which becomes slightly less significant when controlling for education and income (Model 2, *b* =  − 0.173, *p* < 0.05). MDMA is not significant in Model 3, but once controlling for education and income in Model 4, MDMA becomes significantly associated with higher levels of distress (*b* = 0.184, *p* < 0.05). Results also found that both psilocybin (*b* =  − 0.278, *p* < 0.01) and peyote/mescaline (*b* =  − 0.447, *p* < 0.001) were associated with less distress in Model 4, which is slightly less significant after controlling for education and income.

Among non-Hispanic whites (Supplemental Table 2), LCPU is associated with less psychological distress (Model 2, *b* =  − 0.147, *p* < 0.1) but only at the 0.1 significance level. Model 4 shows that unlike the total population, there is no association between MDMA and distress for non-Hispanic whites. Furthermore, psilocybin (*b* =  − 0.343, *p* < 0.001) and peyote/mescaline (*b* =  − 0.535, *p* < 0.001) are strongly associated with less psychological distress. While LSD is significantly associated with more psychological distress among whites (Model 3, *b* = 0.387, *p* < 0.001), the association lost all significance when controlling for distress in Model 4.

Among non-Hispanic Black people (Supplemental Table 3), LCPU is associated with less psychological distress (Model 1, *b* =  − 0.693, *p* < 0.05), but the association loses all significance when controlling for education and income. While psilocybin is not associated with higher distress in Model 3, that association becomes larger and more significant once controlling for education and income (Model 4, *b* = 1.391, *p* < 0.001). Peyote/mescaline is associated with less distress and does not change much when controlling for education and income (Model 4, *b* =  − 1.090, *p* < 0.05).

Like non-Hispanic Blacks, among Hispanics (Supplemental Table 4), only psilocybin is associated with less psychological distress (Model 3, *b* =  − 0.656, *p* < 0.05), but the association loses all significance when controlling for education and income.

Finally, among non-Hispanic Asian people (Supplemental Table 5), while DMT is associated with more distress (Model 3, *b* = 8.540, *p* < 0.01), ayahuasca is associated with less distress (Model 3, *b* =  − 2.947, *p* < 0.01). A post-estimation analysis reveals that there is no statistical change in these associations once controlling for education and income in Model 4.

Overall, results indicate that socioeconomic status is a significant contributor to psychedelic use and returns. Among the total population, socioeconomic inequality may partially reduce the positive association of LCPU, psilocybin, and peyote/mescaline on distress. It is also possible that socioeconomic hardships may also lead to higher MDMA use among those who are most distressed. Among whites, those who use LSD may also be experiencing socioeconomic hardships. In contrast, the distress associated with socioeconomic inequality appears to be driving, in part, Black people’s psilocybin use. Results also indicate that for both Black and Hispanic people, socioeconomic inequality is potentially eliminating some benefits associated with psychedelics use. On the other hand, socioeconomic status may play less of a role in the relationships of distress and psychedelic use for Asians compared to other race/ethnic groups.

### Does Education and Income Moderate the Association Between Psychedelics and Distress?

Interaction terms reveal that among the total population (Supplemental Table 1), the negative association between education and distress was significantly larger for LCPU (Model 5, *b* =  − 0.195, *p* < 0.001) and LSD (Model 11, *b* =  − 0.211, *p* < 0.01). Also, the negative association between family income and distress was significantly larger by LCPU (Model 12, *b* =  − 0.0570, *p* < 0.1) and LSD (Model 18, *b* =  − 0.672, *p* < 0.05). These patterns remained consistent among white, non-Hispanics (Supplemental Table 2), although sometimes less significant. For whites, the negative association between education psychedelics was larger for LCPU (Model 5, *b* =  − 0.167, *p* < 0.01) and LSD (Model 11, *b* =  − 201, *p* < 0.01), while the association of family income was larger LCPU (Model 12, *b* =  − 0.0595, *p* < 0.1) and LSD (Model 18, *b* =  − 0.646, *p* < 0.01).

For non-Hispanic Blacks (Supplemental Table 3), only DMT significantly amplified the negative association between education (Model 8, *b* =  − 18.60, *p* < 0.001) and family income (Model 15, *b* =  − 18.60, *p* < 0.001). Among Hispanics (Supplemental Table 4), no psychedelic amplified the negative associations between education and income on distress. Among non-Hispanic Asians (Supplemental Table 5), only LDS (Model 18, *b* =  − 0.550, *p* < 0.001) amplified the negative association between family income and distress. However, it was also found that the negative association of education and distress was smaller by DMT (Model 8, *b* = 4.457, *p* < 0.001), while the negative association between family income and distress was smaller by DMT (Model 15, *b* = 3.149, *p* < 0.01) and peyote/mescaline (Model 17, *b* = 0.701, *p* < 0.001).

Results indicate that psychedelic use was associated with better outcomes for those individuals with higher levels of education and income. Additionally, this pattern was persistent for white people: the association between psychedelic use and distress was more pronounced among higher-educated and higher-income white individuals. However, regardless of socioeconomic status, the use of psychedelics among Hispanics was not associated with better outcomes. On the other hand, only DMT use was associated for Black people with higher levels of education. Similarly, LSD use among Asians with high income was associated with less stress. Unfortunately, Asians with higher income who used DMT or peyote/mescaline also reported higher levels of distress. Overall, these findings support both hypotheses of diminished psychedelic returns among minorities and the potential impact of inequality associated with socioeconomic status.

## Discussion

This study investigated the association of psychedelic use with mental health outcomes among different racial groups in the general population. The research aimed to answer two main questions: does socioeconomic inequality diminish the effects of psychedelics on health outcomes for minority populations? And, despite having higher socioeconomic status, do minorities experience the same health benefits from psychedelic use as non-Hispanic whites? The results support our predictions and show that socioeconomic status significantly affects psychedelic health outcomes. While some psychedelic use was linked to less distress for Black and Hispanic individuals, these associations became non-significant when accounting for socioeconomic inequality. However, psychedelic use remained correlated with lower distress levels for non-Hispanic whites, even when controlling for socioeconomic status. Higher education and income strengthened the positive association between LSD and LCPU use and reduced distress for non-Hispanic whites. Among higher socioeconomic status non-Hispanic Blacks, only DMT was associated with less distress. For Hispanics, no psychedelic amplified the negative associations between education, income, and distress. Only LSD was associated with lower distress levels for higher-income non-Hispanic Asians, but some instances showed higher distress among Asians with higher education and income who used psychedelics. These findings demonstrate that Black and Hispanic individuals benefit less from psychedelic use due to socioeconomic inequality. Non-Hispanic Asians with higher socioeconomic status may be using psychedelics as a form of self-medication, possibly due to cultural stigma around mental health. These implications align with previous research showing that education and income may not always lead to better mental or physical health outcomes [6, 7] and emotional wellbeing [9]. Systemic inequality negatively affects health outcomes related to psychedelics for minority populations, similar to other health research.

Why is not psychedelic use associated with better mental health outcomes for Black and Hispanic individuals? The answer lies in the fundamental role of racism in contributing to adverse health outcomes and inequities for racial and ethnic minorities [89, 90], which finds discrimination leads to poorer physical health outcomes for minorities. Black individuals, for instance, are more likely to experience pervasive and everyday mistreatment, such as receiving less courtesy or poor commercial services, which is linked to worse allostatic load, sleep duration and efficiency, systemic inflammation, distress and mental illness, negative coping behaviors like smoking, and late-life cognitive function [1, 59, 60, 75, 84, 94]. Similarly, Latinos also report higher rates of discrimination and ethnic-based violence, leading to increased substance use, daily distress, and mental health issues [2, 3, 13, 16, 81]. Research on fundamental causality highlights the structural conditions that impact the efficacy of health interventions and perpetuate health inequalities along social fault lines of race, class, and gender [28, 47, 65]. Psychedelics are just one intervention, while social stratification influences numerous diseases, disease risk factors, and access to resources that help mitigate health risks. Since psychedelic benefits depend on psychosocial conditions, we should expect that the same conditions that undermine the health of Black and Latino individuals in other areas will persist for mental health.

Importantly, similar to research that suggests education may be a risk factor for depression in Black men [5], these findings indicate that psilocybin is associated with poorer mental health outcomes for Black people, but this association is only significant after accounting for socioeconomic status. Conversely, LSD is linked to increased distress in whites, but this relationship loses significance when socioeconomic status is included in the models. These findings may be explained in a few ways. It is possible that these whites with lower economic status may be using LSD recreationally, while the distress caused by socioeconomic inequality directly influences the use of psilocybin among Black people. These findings once again underscore the detrimental impact of socioeconomic inequality on drug use among Black individuals: limited access to healthcare, which is significantly lower for Black people [80], drives Black people to negative coping strategies including smoking and drinking [8]. Perhaps the use of psychedelics is another strategy employed by this population.

The study revealed a notable and unexpected outcome regarding the impact of DMT on socioeconomic status. It found that Black individuals with higher socioeconomic status experienced less distress when using DMT. In contrast, among Asians with higher socioeconomic status, DMT was associated with increased distress. These findings are especially remarkable given the study’s modest sample size of only 370 DMT users and the general lower rates of drug use among minority populations. The significance of DMT in this context may be related to the characteristics of the individuals who use it. While DMT is classified as a classic psychedelic, it stands out due to its unique origins and usage. Unlike other psychedelics that are easily accessible, DMT is more challenging to obtain on the underground market [23, 29]. Additionally, while other psychedelics are typically consumed orally, DMT is most commonly obtained through the venom of the *Bufo alvarius* toad and smoked. Furthermore, DMT trips have a much shorter duration, lasting on average only 20 min compared to the hours-long trips of other psychedelics [83]. It is likely that these unique consumption and usage aspects of DMT are capturing sociocultural differences, specifically attracting a particular type of person with class privileges who possesses the knowledge to obtain and use it for health or spiritual purposes. In fact, DMT was initially referred to as the “businessman’s trip” due to its short duration, making it particularly appealing to individuals seeking the benefits of psychedelics but with limited time to use other psychoactive drugs [78].

Additionally, it is important to consider the interaction results related to DMT among Asians, as they do not necessarily indicate that socioeconomic status hinders access. On the contrary, the findings suggest that Asians with higher socioeconomic status may be more likely to use DMT for self-mediation purposes. These results could be explained by cultural stigma. It is worth noting that Asians report high rates of discrimination and stigma associated with mental illness [77, 92, 95]. Research has shown that Asians with mental illness experience higher rates of discrimination compared to whites [81]. Asians also report cultural stigma attached to mental illness and drug use [42, 77, 95]. In fact, Asians across all ethnic groups have the lowest rates of mental health treatment and the highest unmet mental health needs during the COVID-19 pandemic [51]. A systematic review by Zhang and colleagues (2019 found that Asians were more likely to view those with mental illness as dangerous and aggressive,see all psychiatric illnesses as socially unacceptable and a personal weakness and consider mental illness as shameful to the family. Furthermore, Asian Americans have negative views of drug use, associating it with bringing shame to the family and experiencing a cultural clash between their traditional Asian identity and harmful acculturation towards an American identity [56]. Therefore, these results may be capturing Asian with higher socioeconomic status who avoid mental health treatment due to cultural stigma but then access DMT due to their privileged social class.

### Limitations and Future Research

While this study uncovers significant connections between race, socioeconomic status, psychedelics, and mental health, it is important to note its limitations. The primary limitation lies in the data itself, as it does not provide a comprehensive understanding of the long-term effects of psychedelics on minorities. To address this, longitudinal data that includes information on the timing of drug use would be required. Additionally, there could be other unmeasured factors that contribute to the observed associations. Despite the inclusion of various standard control variables, it is likely that additional factors were overlooked. For instance, personality traits, the occurrence and response to peak experiences, and dosage have all been shown to impact outcomes in previous psychedelic clinical trials. Given our focus on socioeconomic inequality, factors like access to healthcare or occupational types may further elucidate these disparities.

Most importantly, the results of this study cannot be used to draw definitive causal inferences due to its cross-sectional design. This is especially true because we lack information about the motivations behind the use of psychedelics. While it is likely that some individuals use psychedelics as a response to distress caused by socioeconomic inequality, without specific measures, we cannot be certain. On the other hand, those who use psychedelics in a clinical setting are likely doing so for health benefits. However, this paper intentionally adopts a modified cultural set and setting theoretical approach, which emphasizes the influence of cultural differences and social inequality on the effectiveness of certain drugs. By employing this enhanced theory, the objective of this study is to demonstrate disparities in outcomes related to drug use by examining broader epidemiological patterns. This strategy aligns closely with the research approach of this study. By establishing a solid foundation to highlight disparities at a macro level, this study sets the stage for future investigations to analyze these differences from a micro perspective. While I anticipate that a more detailed examination of the relationship between race, mental health, and psychedelic use would offer a more nuanced explanation, I do not expect it to significantly alter the results found in this paper, particularly in terms of the implications of structural racism on health.

There are several important avenues for future research in the field. Firstly, it is crucial to replicate these findings across other marginalized groups, such as Indian Americans, women, immigrants, and those who identify as LGBTQ + . By doing so, we can gain a more comprehensive understanding of how structural inequality affects psychedelic use and outcomes among different minority populations. Secondly, future research should aim to collect more precise indicators of structural inequality and motivations for psychedelic use. This will enable us to better comprehend the negative impact of structural inequality on Black individuals’ psychedelic use and outcomes. It is also important to consider the potential influence of existing neighborhood and racial segregation on unequal psychedelic outcomes. Previous studies have shown that neighborhood and racial segregation can lead to various negative consequences like substance use, higher stress, and chronic illness [34, 46]. Future research should aim to identify the specific aspects of structural inequality that have the largest negative effects on MDPR and prioritize addressing them to achieve the best outcomes for minority populations.

## Conclusion

Despite its limitations, this study contributes valuable insights to the growing body of research on the relationship between psychedelics and health. It is important to consider race/ethnic results from a population-level perspective, focusing on the lifetime use of classic psychedelics, rather than solely relying on clinical trials. These findings provide a glimpse into how the general population interacts with these substances in their everyday lives, as opposed to a controlled clinical setting. With the increasing availability of psychedelics, their naturalistic use is likely to surpass clinical treatment, particularly in areas with limited mental health resources. Furthermore, regardless of the underlying motivation (whether clinical or recreational), psychedelics have been associated with improved mental health outcomes for white populations based on various indicators. However, this study reveals that socioeconomic inequality plays a significant role in determining the psychedelic benefits experienced by minority populations.

These results are also significance due to the limited exploration of the relationship between marginalized groups and psychedelics, as well as the lack of diversity in current clinical psychedelic research. A comprehensive analysis of contemporary clinical trials on psychedelics has revealed that more than 80% of study participants are white, leading to a significant underrepresentation of BIPOC individuals [79]. This underrepresentation raises concerns about the generalizability of findings to non-white populations [24, 53]. As scholars who study the intersectionality of race and gender have demonstrated [17], the lived experiences of marginalized individuals vary significantly. Therefore, results obtained from a predominantly class-privileged Asian sample may not be applicable to the lives of Black and Indigenous populations.

Furthermore, despite a lack of research, some researchers argue that psychedelics could have a positive impact on the health of BIPOC individuals and could potentially serve as a means to alleviate the negative psychological effects of racism. Smith et al. [76], p. 11) highlight the seriousness and prevalence of trauma and PTSD resulting from racism, suggesting that psychedelics may offer a viable solution to address the consequences of race-based experiences. Another study suggests that psychedelics may have the potential to help immigrants overcome incidents of discrimination, promote positive social and health behaviors, and foster feelings of connection to others [43]. In an effort to promote inclusivity, the MAPS Bulletin documents two events that provided MDMA therapy training specifically for communities of color [12]. Despite the challenges faced by the program, the authors believe that initiatives like theirs are crucial in making psychedelic therapy more accessible to people of color.

MDPR raises concerns about the optimistic belief that psychedelics can effectively address the effects of racism, discrimination, or other inequality. This perspective fails to acknowledge the need for structural change and instead perpetuates the idea that marginalized individuals should lift themselves up without addressing the root causes of discrimination [69]. Moreover, this theory calls into questions those who suggests that marginalized individuals should rely on drugs to numb themselves from societal discrimination. While future research may uncover potential benefits for racial and ethnic minorities in controlled clinical settings, the decriminalization of these drugs should still be pursued. Nevertheless, MDPR argues against viewing psychedelics as a cure-all solution, as the availability of these interventions primarily benefits privileged white individuals. Even if marginalized individuals experience benefits within clinical settings, it remains uncertain whether they will continue to enjoy these benefits in their everyday lives. MDR scholars emphasize that enforcing civil rights is crucial for reducing health inequalities, as it grants marginalized individuals access to social and psychological resources that can improve their sense of self-respect and overall health [26]. Without addressing racism and structural inequality, which undoubtedly impact all aspects of the psychedelic health field, these drugs are unlikely to significantly improve the health of marginalized groups, thus exacerbating existing health disparities.

## Supplementary Information

Below is the link to the electronic supplementary material.

Supplementary file1 (DOCX 299 KB)

## Data Availability

This project uses secondary data from the NSDUH which is available for download at https://www.datafiles.samhsa.gov/dataset/nsduh-2002-2019-ds0001-nsduh-2002-2019-ds0001

## Footnotes

1 The NSDUH asks two questions about the use of DMT. The first question asks about lifetime use of DMT. The second question asks about the use of “DMT, AMT (alpha-methyltryptamine) or 5-MeO-DIPT?” The second variable was excluded because neither AMT nor 5-MeO-DIPT are classified as classic psychedelics, and DMT use alone could not be determined from the question.

2 Non-Hispanic Native American/Alaska Native, non-Hispanic Native Hawaiian/Pacific Islander, and “other” non-Hispanics were not considered in the main analysis of race/ethnic differences because the sample sizes for these groups were too small to conduct a meaningful regression analysis.

3 I followed recommendation to run a sensitivity analysis of alternative models (i.e., probit, multinomial, and ordinary least-square regression), but results proved substantially identical [22].

## Code Availability

Stata coding will be made available upon request by contacting the contributing author.
