# Supplementary material for: Minorities’ Diminished Psychedelic Returns: Income and Educations Impact on Whites, Blacks, Hispanics, and Asians
Source: J Racial Ethn Health Disparities. 2024 May 16;12(3):1937–50. doi: 10.1007/s40615-024-02023-y (PMC12069501; doi:10.1007/s40615-024-02023-y)
Supplement: Supplementary file 1 — Supplementary file1 (DOCX 299 KB) [file 40615_2024_2023_MOESM1_ESM.docx]

Supplemental Tables

| Supplemental Table 1A. Weighted Multivariate Ordinary Squared Logistics Regression Predicting Psychological Distress Levels In the Past 30 Days Among the Total Population | | | | |
| --- | --- | --- | --- | --- |
|  | Model 1 | Model 2 | Model 3 | Model 4 |
| Age | -0.594^***^ | -0.555^***^ | -0.592^***^ | -0.552^***^ |
|  | (0.0115) | (0.0111) | (0.0117) | (0.0113) |
|  |  |  |  |  |
| ^a^Women | 0.944^***^ | 0.926^***^ | 0.933^***^ | 0.917^***^ |
|  | (0.0493) | (0.0485) | (0.0489) | (0.0480) |
|  |  |  |  |  |
| ^b^Married | -0.916^***^ | -0.402^***^ | -0.920^***^ | -0.404^***^ |
|  | (0.0624) | (0.0620) | (0.0628) | (0.0622) |
|  |  |  |  |  |
| Divorced | -0.101 | -0.451^***^ | -0.107 | -0.453^***^ |
|  | (0.121) | (0.120) | (0.122) | (0.121) |
|  |  |  |  |  |
| Widowed | 0.612^***^ | 0.473^***^ | 0.607^***^ | 0.472^***^ |
|  | (0.0906) | (0.0899) | (0.0912) | (0.0903) |
|  |  |  |  |  |
| ^c^Black | -0.0668 | -0.424^***^ | -0.0722 | -0.428^***^ |
|  | (0.0713) | (0.0709) | (0.0710) | (0.0705) |
|  |  |  |  |  |
| Hispanic | 0.374^***^ | -0.00177 | 0.371^***^ | -0.00303 |
|  | (0.0761) | (0.0784) | (0.0761) | (0.0785) |
|  |  |  |  |  |
| Asian | -0.0162 | 0.314^**^ | -0.0160 | 0.313^**^ |
|  | (0.101) | (0.0996) | (0.101) | (0.0997) |
|  |  |  |  |  |
| Native American | 1.335^***^ | 0.782^**^ | 1.367^***^ | 0.816^**^ |
|  | (0.272) | (0.260) | (0.273) | (0.262) |
|  |  |  |  |  |
| Hawaiian/Pacific I | 0.619 | 0.275 | 0.616 | 0.272 |
|  | (0.406) | (0.432) | (0.406) | (0.432) |
|  |  |  |  |  |
| Multi Racial | 0.583^***^ | 0.320^*^ | 0.595^***^ | 0.331^*^ |
|  | (0.159) | (0.158) | (0.159) | (0.157) |
|  |  |  |  |  |
| Religious Salience | 0.00846 | -0.0425^***^ | 0.00755 | -0.0429^***^ |
|  | (0.0114) | (0.0114) | (0.0114) | (0.0114) |
|  |  |  |  |  |
| Religious Attendance | -0.201^***^ | -0.125^***^ | -0.201^***^ | -0.126^***^ |
|  | (0.0156) | (0.0150) | (0.0157) | (0.0151) |
|  |  |  |  |  |
| Cocaine | 0.0572 | -0.0640 | 0.0700 | -0.0451 |
|  | (0.0702) | (0.0689) | (0.0714) | (0.0701) |
|  |  |  |  |  |
| Stimulants | 0.591^***^ | 0.611^***^ | 0.599^***^ | 0.619^***^ |
|  | (0.0718) | (0.0692) | (0.0719) | (0.0692) |
|  |  |  |  |  |
| Sedatives | 0.852^***^ | 0.878^***^ | 0.870^***^ | 0.897^***^ |
|  | (0.0741) | (0.0723) | (0.0743) | (0.0726) |
|  |  |  |  |  |
| Tranquilizers | 1.092^***^ | 1.062^***^ | 1.092^***^ | 1.061^***^ |
|  | (0.0562) | (0.0545) | (0.0563) | (0.0546) |
|  |  |  |  |  |
| Heroine | 1.082^***^ | 0.693^***^ | 1.108^***^ | 0.724^***^ |
|  | (0.153) | (0.149) | (0.151) | (0.147) |
|  |  |  |  |  |
| Pain Killers | 0.375^***^ | 0.369^***^ | 0.378^***^ | 0.371^***^ |
|  | (0.0640) | (0.0633) | (0.0640) | (0.0633) |
|  |  |  |  |  |
| Marijuana | 0.297^***^ | 0.409^***^ | 0.290^***^ | 0.405^***^ |
|  | (0.0715) | (0.0699) | (0.0716) | (0.0700) |
|  |  |  |  |  |
| PCP | 0.178 | -0.0332 | 0.249 | 0.0578 |
|  | (0.187) | (0.183) | (0.186) | (0.181) |
|  |  |  |  |  |
| Inhalants | 0.482^***^ | 0.542^***^ | 0.506^***^ | 0.563^***^ |
|  | (0.0756) | (0.0747) | (0.0756) | (0.0746) |
|  |  |  |  |  |
| Tobacco | 0.649^***^ | 0.449^***^ | 0.645^***^ | 0.445^***^ |
|  | (0.0535) | (0.0548) | (0.0535) | (0.0548) |
|  |  |  |  |  |
| Age of 1^st^ Alcohol Use | 0.0652^*^ | -0.0268 | 0.0656^*^ | -0.0266 |
|  | (0.0297) | (0.0282) | (0.0296) | (0.0281) |
|  |  |  |  |  |
| Risk Behaviors | 0.537^***^ | 0.655^***^ | 0.540^***^ | 0.657^***^ |
|  | (0.0317) | (0.0310) | (0.0316) | (0.0310) |
|  |  |  |  |  |
| ^d^2009 | -0.0167 | -0.0305 | -0.0157 | -0.0294 |
|  | (0.115) | (0.120) | (0.115) | (0.121) |
|  |  |  |  |  |
| 2010 | 0.111 | 0.0593 | 0.113 | 0.0603 |
|  | (0.122) | (0.117) | (0.122) | (0.117) |
|  |  |  |  |  |
| 2011 | 0.0996 | 0.0691 | 0.103 | 0.0717 |
|  | (0.114) | (0.116) | (0.114) | (0.116) |
|  |  |  |  |  |
| 2012 | 0.186 | 0.168 | 0.189 | 0.171 |
|  | (0.120) | (0.118) | (0.121) | (0.119) |
|  |  |  |  |  |
| 2013 | 0.240^†^ | 0.290^*^ | 0.243^†^ | 0.291^*^ |
|  | (0.141) | (0.142) | (0.140) | (0.142) |
|  |  |  |  |  |
| 2014 | 0.0655 | 0.104 | 0.0645 | 0.101 |
|  | (0.103) | (0.104) | (0.104) | (0.104) |
|  |  |  |  |  |
| 2015 | -0.413^***^ | -0.318^**^ | -0.415^***^ | -0.320^**^ |
|  | (0.120) | (0.118) | (0.120) | (0.118) |
|  |  |  |  |  |
| 2016 | -0.300^**^ | -0.204^†^ | -0.305^**^ | -0.208^†^ |
|  | (0.114) | (0.112) | (0.115) | (0.113) |
|  |  |  |  |  |
| 2017 | -0.207^†^ | -0.0562 | -0.214^†^ | -0.0626 |
|  | (0.118) | (0.118) | (0.118) | (0.118) |
|  |  |  |  |  |
| 2018 | 0.0748 | 0.240^†^ | 0.0696 | 0.234^†^ |
|  | (0.124) | (0.123) | (0.123) | (0.122) |
|  |  |  |  |  |
| 2019 | 0.357^**^ | 0.567^***^ | 0.349^**^ | 0.559^***^ |
|  | (0.128) | (0.127) | (0.127) | (0.126) |
|  |  |  |  |  |
| MDMA | 0.144 | 0.188^*^ | 0.147 | 0.184^*^ |
|  | (0.0867) | (0.0845) | (0.0878) | (0.0851) |
|  |  |  |  |  |
| LCPU | -0.185^**^ | -0.173^*^ |  |  |
|  | (0.0669) | (0.0664) |  |  |
|  |  |  |  |  |
| Educational Level |  | -0.418^***^ |  | -0.414^***^ |
|  |  | (0.0250) |  | (0.0253) |
|  |  |  |  |  |
| Family Income |  | -0.365^***^ |  | -0.366^***^ |
|  |  | (0.0128) |  | (0.0128) |
|  |  |  |  |  |
| Psilocybin |  |  | -0.375^***^ | -0.278^**^ |
|  |  |  | (0.0860) | (0.0851) |
|  |  |  |  |  |
| DMT |  |  | 0.368 | 0.565 |
|  |  |  | (0.616) | (0.597) |
|  |  |  |  |  |
| Ayahuasca |  |  | 1.023 | 1.333 |
|  |  |  | (1.699) | (1.704) |
|  |  |  |  |  |
| Peyote/Mescaline |  |  | -0.436^**^ | -0.447^***^ |
|  |  |  | (0.129) | (0.126) |
|  |  |  |  |  |
| LSD |  |  | 0.211^*^ | 0.126 |
|  |  |  | (0.0819) | (0.0827) |
|  |  |  |  |  |
| Constant | 15.77^***^ | 18.27^***^ | 15.75^***^ | 18.23^***^ |
|  | (0.210) | (0.227) | (0.213) | (0.230) |
| Observations | 149002 | 149002 | 149002 | 149002 |
| *R*^2^ | 0.127 | 0.148 | 0.127 | 0.148 |
| Source: 2008-2019 National Survey of Drug Use and Health, N=458,372  Standard errors in parentheses  ^a^ Men serve as the references category  ^b^ Single Serves as the reference category for marital statuses  ^c^ White, Non-Hispanic Serves as the reference category for race/ethnicity  ^d^ 2008 serves as the reference category for the year survey was taken  ^†^ *p* < 0.1, ^*^ *p* < 0.05, ^**^ *p* < 0.01, ^***^ *p* < 0.001 | | | | |

| Supplemental Table 1B. Weighted Multivariate Ordinary Squared Logistics Regression Predicting Psychological Distress Levels In the Past 30 Days Among the Total Population with Interaction Terms (Psychedelics * Educational Level) | | | | | | | |
| --- | --- | --- | --- | --- | --- | --- | --- |
|  | Model 5 | Model 6 | Model 7 | Model 8 | Model 9 | Model 10 | Model 11 |
| Age | -0.553^***^ | -0.551^***^ | -0.552^***^ | -0.552^***^ | -0.552^***^ | -0.552^***^ | -0.551^***^ |
|  | (0.0111) | (0.0112) | (0.0113) | (0.0113) | (0.0113) | (0.0113) | (0.0113) |
|  |  |  |  |  |  |  |  |
| ^a^Women | 0.929^***^ | 0.918^***^ | 0.918^***^ | 0.917^***^ | 0.917^***^ | 0.917^***^ | 0.920^***^ |
|  | (0.0484) | (0.0480) | (0.0479) | (0.0480) | (0.0480) | (0.0481) | (0.0479) |
|  |  |  |  |  |  |  |  |
| ^b^Married | -0.402^***^ | -0.404^***^ | -0.403^***^ | -0.404^***^ | -0.404^***^ | -0.403^***^ | -0.404^***^ |
|  | (0.0621) | (0.0623) | (0.0623) | (0.0622) | (0.0623) | (0.0623) | (0.0625) |
|  |  |  |  |  |  |  |  |
| Divorced | -0.443^***^ | -0.453^***^ | -0.452^***^ | -0.454^***^ | -0.454^***^ | -0.451^***^ | -0.445^***^ |
|  | (0.120) | (0.121) | (0.121) | (0.121) | (0.121) | (0.121) | (0.121) |
|  |  |  |  |  |  |  |  |
| Widowed | 0.470^***^ | 0.471^***^ | 0.471^***^ | 0.472^***^ | 0.472^***^ | 0.472^***^ | 0.470^***^ |
|  | (0.0899) | (0.0903) | (0.0903) | (0.0903) | (0.0903) | (0.0903) | (0.0903) |
|  |  |  |  |  |  |  |  |
| ^c^Black | -0.415^***^ | -0.427^***^ | -0.426^***^ | -0.428^***^ | -0.428^***^ | -0.426^***^ | -0.420^***^ |
|  | (0.0712) | (0.0706) | (0.0705) | (0.0706) | (0.0705) | (0.0704) | (0.0708) |
|  |  |  |  |  |  |  |  |
| Hispanic | 0.00883 | -0.000672 | -0.000745 | -0.00312 | -0.00284 | -0.000730 | 0.00652 |
|  | (0.0786) | (0.0788) | (0.0784) | (0.0785) | (0.0785) | (0.0783) | (0.0788) |
|  |  |  |  |  |  |  |  |
| Asian | 0.301^**^ | 0.312^**^ | 0.311^**^ | 0.313^**^ | 0.313^**^ | 0.310^**^ | 0.302^**^ |
|  | (0.0992) | (0.0995) | (0.0993) | (0.0997) | (0.0997) | (0.0994) | (0.0995) |
|  |  |  |  |  |  |  |  |
| Native American | 0.778^**^ | 0.814^**^ | 0.816^**^ | 0.816^**^ | 0.816^**^ | 0.814^**^ | 0.819^**^ |
|  | (0.260) | (0.261) | (0.261) | (0.262) | (0.262) | (0.261) | (0.262) |
|  |  |  |  |  |  |  |  |
| Hawaiian/Pacific I | 0.280 | 0.272 | 0.273 | 0.272 | 0.272 | 0.274 | 0.274 |
|  | (0.432) | (0.432) | (0.432) | (0.432) | (0.432) | (0.432) | (0.432) |
|  |  |  |  |  |  |  |  |
| Multi Racial | 0.321^*^ | 0.332^*^ | 0.331^*^ | 0.331^*^ | 0.330^*^ | 0.331^*^ | 0.330^*^ |
|  | (0.157) | (0.157) | (0.157) | (0.157) | (0.157) | (0.157) | (0.157) |
|  |  |  |  |  |  |  |  |
| Religious Salience | -0.0429^***^ | -0.0431^***^ | -0.0430^***^ | -0.0429^***^ | -0.0429^***^ | -0.0430^***^ | -0.0431^***^ |
|  | (0.0114) | (0.0113) | (0.0114) | (0.0114) | (0.0114) | (0.0114) | (0.0114) |
|  |  |  |  |  |  |  |  |
| Religious Attendance | -0.127^***^ | -0.126^***^ | -0.126^***^ | -0.126^***^ | -0.126^***^ | -0.126^***^ | -0.127^***^ |
|  | (0.0150) | (0.0151) | (0.0150) | (0.0151) | (0.0151) | (0.0151) | (0.0150) |
|  |  |  |  |  |  |  |  |
| Cocaine | -0.0648 | -0.0439 | -0.0443 | -0.0451 | -0.0446 | -0.0433 | -0.0465 |
|  | (0.0688) | (0.0702) | (0.0702) | (0.0701) | (0.0700) | (0.0701) | (0.0700) |
|  |  |  |  |  |  |  |  |
| Stimulants | 0.614^***^ | 0.621^***^ | 0.619^***^ | 0.619^***^ | 0.619^***^ | 0.619^***^ | 0.621^***^ |
|  | (0.0693) | (0.0695) | (0.0692) | (0.0692) | (0.0692) | (0.0692) | (0.0692) |
|  |  |  |  |  |  |  |  |
| Sedatives | 0.882^***^ | 0.897^***^ | 0.897^***^ | 0.897^***^ | 0.897^***^ | 0.898^***^ | 0.902^***^ |
|  | (0.0722) | (0.0726) | (0.0726) | (0.0726) | (0.0726) | (0.0725) | (0.0725) |
|  |  |  |  |  |  |  |  |
| Tranquilizers | 1.060^***^ | 1.060^***^ | 1.061^***^ | 1.061^***^ | 1.061^***^ | 1.061^***^ | 1.059^***^ |
|  | (0.0544) | (0.0546) | (0.0546) | (0.0546) | (0.0546) | (0.0546) | (0.0545) |
|  |  |  |  |  |  |  |  |
| Heroine | 0.652^***^ | 0.710^***^ | 0.714^***^ | 0.724^***^ | 0.724^***^ | 0.714^***^ | 0.687^***^ |
|  | (0.148) | (0.148) | (0.146) | (0.147) | (0.147) | (0.146) | (0.147) |
|  |  |  |  |  |  |  |  |
| Pain Killers | 0.361^***^ | 0.368^***^ | 0.369^***^ | 0.371^***^ | 0.371^***^ | 0.370^***^ | 0.364^***^ |
|  | (0.0633) | (0.0634) | (0.0633) | (0.0633) | (0.0633) | (0.0633) | (0.0632) |
|  |  |  |  |  |  |  |  |
| Marijuana | 0.410^***^ | 0.405^***^ | 0.405^***^ | 0.405^***^ | 0.405^***^ | 0.405^***^ | 0.404^***^ |
|  | (0.0699) | (0.0701) | (0.0700) | (0.0700) | (0.0701) | (0.0700) | (0.0700) |
|  |  |  |  |  |  |  |  |
| PCP | -0.0773 | 0.0479 | 0.0479 | 0.0581 | 0.0577 | 0.0435 | 0.0132 |
|  | (0.183) | (0.181) | (0.181) | (0.181) | (0.181) | (0.180) | (0.183) |
|  |  |  |  |  |  |  |  |
| Inhalants | 0.549^***^ | 0.566^***^ | 0.565^***^ | 0.563^***^ | 0.563^***^ | 0.565^***^ | 0.570^***^ |
|  | (0.0745) | (0.0746) | (0.0745) | (0.0746) | (0.0746) | (0.0747) | (0.0745) |
|  |  |  |  |  |  |  |  |
| Tobacco | 0.453^***^ | 0.446^***^ | 0.446^***^ | 0.445^***^ | 0.445^***^ | 0.446^***^ | 0.449^***^ |
|  | (0.0548) | (0.0549) | (0.0549) | (0.0548) | (0.0548) | (0.0548) | (0.0548) |
|  |  |  |  |  |  |  |  |
| Age of 1^st^ Alcohol Use | -0.0236 | -0.0259 | -0.0259 | -0.0266 | -0.0265 | -0.0260 | -0.0241 |
|  | (0.0283) | (0.0282) | (0.0283) | (0.0281) | (0.0281) | (0.0282) | (0.0283) |
|  |  |  |  |  |  |  |  |
| Risk Behaviors | 0.654^***^ | 0.656^***^ | 0.656^***^ | 0.657^***^ | 0.657^***^ | 0.656^***^ | 0.655^***^ |
|  | (0.0310) | (0.0310) | (0.0311) | (0.0310) | (0.0310) | (0.0310) | (0.0310) |
|  |  |  |  |  |  |  |  |
| ^d^2009 | -0.0316 | -0.0301 | -0.0295 | -0.0294 | -0.0295 | -0.0293 | -0.0294 |
|  | (0.120) | (0.121) | (0.121) | (0.121) | (0.121) | (0.121) | (0.120) |
|  |  |  |  |  |  |  |  |
| 2010 | 0.0614 | 0.0604 | 0.0603 | 0.0603 | 0.0600 | 0.0605 | 0.0626 |
|  | (0.118) | (0.118) | (0.118) | (0.117) | (0.117) | (0.118) | (0.118) |
|  |  |  |  |  |  |  |  |
| 2011 | 0.0680 | 0.0707 | 0.0711 | 0.0716 | 0.0717 | 0.0709 | 0.0723 |
|  | (0.116) | (0.116) | (0.116) | (0.116) | (0.116) | (0.116) | (0.116) |
|  |  |  |  |  |  |  |  |
| 2012 | 0.168 | 0.170 | 0.170 | 0.171 | 0.170 | 0.171 | 0.170 |
|  | (0.118) | (0.119) | (0.119) | (0.119) | (0.119) | (0.119) | (0.119) |
|  |  |  |  |  |  |  |  |
| 2013 | 0.292^*^ | 0.290^*^ | 0.292^*^ | 0.291^*^ | 0.291^*^ | 0.291^*^ | 0.294^*^ |
|  | (0.142) | (0.142) | (0.142) | (0.142) | (0.142) | (0.142) | (0.142) |
|  |  |  |  |  |  |  |  |
| 2014 | 0.103 | 0.101 | 0.100 | 0.101 | 0.101 | 0.101 | 0.100 |
|  | (0.104) | (0.104) | (0.104) | (0.104) | (0.104) | (0.104) | (0.104) |
|  |  |  |  |  |  |  |  |
| 2015 | -0.314^**^ | -0.319^**^ | -0.319^**^ | -0.320^**^ | -0.320^**^ | -0.320^**^ | -0.315^**^ |
|  | (0.118) | (0.118) | (0.118) | (0.118) | (0.118) | (0.118) | (0.118) |
|  |  |  |  |  |  |  |  |
| 2016 | -0.200^†^ | -0.207^†^ | -0.207^†^ | -0.208^†^ | -0.208^†^ | -0.208^†^ | -0.203^†^ |
|  | (0.112) | (0.113) | (0.113) | (0.113) | (0.113) | (0.113) | (0.112) |
|  |  |  |  |  |  |  |  |
| 2017 | -0.0531 | -0.0608 | -0.0622 | -0.0625 | -0.0628 | -0.0626 | -0.0590 |
|  | (0.118) | (0.118) | (0.118) | (0.118) | (0.118) | (0.118) | (0.118) |
|  |  |  |  |  |  |  |  |
| 2018 | 0.242^†^ | 0.235^†^ | 0.235^†^ | 0.234^†^ | 0.234^†^ | 0.234^†^ | 0.238^†^ |
|  | (0.122) | (0.122) | (0.122) | (0.122) | (0.122) | (0.122) | (0.122) |
|  |  |  |  |  |  |  |  |
| 2019 | 0.570^***^ | 0.560^***^ | 0.559^***^ | 0.559^***^ | 0.559^***^ | 0.558^***^ | 0.562^***^ |
|  | (0.126) | (0.126) | (0.126) | (0.126) | (0.126) | (0.127) | (0.126) |
|  |  |  |  |  |  |  |  |
| MDMA | 0.193^*^ | 0.445^*^ | 0.184^*^ | 0.184^*^ | 0.185^*^ | 0.183^*^ | 0.189^*^ |
|  | (0.0840) | (0.217) | (0.0851) | (0.0851) | (0.0851) | (0.0852) | (0.0849) |
|  |  |  |  |  |  |  |  |
| LCPU | 0.405^*^ |  |  |  |  |  |  |
|  | (0.159) |  |  |  |  |  |  |
|  |  |  |  |  |  |  |  |
| Educational Level | -0.381^***^ | -0.405^***^ | -0.407^***^ | -0.414^***^ | -0.414^***^ | -0.407^***^ | -0.384^***^ |
|  | (0.0259) | (0.0265) | (0.0253) | (0.0255) | (0.0253) | (0.0264) | (0.0253) |
|  |  |  |  |  |  |  |  |
| Family Income | -0.366^***^ | -0.366^***^ | -0.366^***^ | -0.366^***^ | -0.366^***^ | -0.366^***^ | -0.367^***^ |
|  | (0.0128) | (0.0128) | (0.0128) | (0.0128) | (0.0128) | (0.0128) | (0.0128) |
|  |  |  |  |  |  |  |  |
| LCPU * Educational Level | -0.195^***^ |  |  |  |  |  |  |
|  | (0.0456) |  |  |  |  |  |  |
|  |  |  |  |  |  |  |  |
| Psilocybin |  | -0.274^**^ | -0.106 | -0.278^**^ | -0.279^**^ | -0.278^**^ | -0.263^**^ |
|  |  | (0.0850) | (0.196) | (0.0851) | (0.0851) | (0.0851) | (0.0855) |
|  |  |  |  |  |  |  |  |
| DMT |  | 0.579 | 0.572 | 0.206 | 0.555 | 0.578 | 0.601 |
|  |  | (0.594) | (0.597) | (1.195) | (0.599) | (0.596) | (0.593) |
|  |  |  |  |  |  |  |  |
| Ayahuasca |  | 1.356 | 1.341 | 1.338 | 5.151 | 1.358 | 1.355 |
|  |  | (1.710) | (1.704) | (1.705) | (5.824) | (1.707) | (1.701) |
|  |  |  |  |  |  |  |  |
| Peyote/Mescaline |  | -0.449^***^ | -0.449^***^ | -0.447^***^ | -0.447^***^ | -0.0771 | -0.440^***^ |
|  |  | (0.126) | (0.126) | (0.126) | (0.126) | (0.371) | (0.126) |
|  |  |  |  |  |  |  |  |
| LSD |  | 0.128 | 0.126 | 0.126 | 0.126 | 0.128 | 0.731^***^ |
|  |  | (0.0827) | (0.0828) | (0.0828) | (0.0826) | (0.0823) | (0.172) |
|  |  |  |  |  |  |  |  |
| MDMA * Educational Level |  | -0.0900 |  |  |  |  |  |
|  |  | (0.0661) |  |  |  |  |  |
|  |  |  |  |  |  |  |  |
| Psilocybin * Educational Level |  |  | -0.0571 |  |  |  |  |
|  |  |  | (0.0560) |  |  |  |  |
|  |  |  |  |  |  |  |  |
| DMT * Educational Level |  |  |  | 0.118 |  |  |  |
|  |  |  |  | (0.435) |  |  |  |
|  |  |  |  |  |  |  |  |
| Ayahuasca * Educational Level |  |  |  |  | -1.160 |  |  |
|  |  |  |  |  | (1.678) |  |  |
|  |  |  |  |  |  |  |  |
| Peyote/Mescaline * Educational Level |  |  |  |  |  | -0.128 |  |
|  |  |  |  |  |  | (0.111) |  |
|  |  |  |  |  |  |  |  |
| LSD * Educational Level |  |  |  |  |  |  | -0.211^***^ |
|  |  |  |  |  |  |  | (0.0579) |
|  |  |  |  |  |  |  |  |
| Constant | 18.14^***^ | 18.20^***^ | 18.20^***^ | 18.23^***^ | 18.23^***^ | 18.21^***^ | 18.13^***^ |
|  | (0.229) | (0.234) | (0.229) | (0.231) | (0.230) | (0.229) | (0.230) |
| Observations | 149002 | 149002 | 149002 | 149002 | 149002 | 149002 | 149002 |
| *R*^2^ | 0.148 | 0.148 | 0.148 | 0.148 | 0.148 | 0.148 | 0.148 |
| Source: 2008-2019 National Survey of Drug Use and Health, N=458,372  Standard errors in parentheses  ^a^ Men serve as the references category  ^b^ Single Serves as the reference category for marital statuses  ^c^ White, Non-Hispanic Serves as the reference category for race/ethnicity  ^d^ 2008 serves as the reference category for the year survey was taken  ^†^ *p* < 0.1, ^*^ *p* < 0.05, ^**^ *p* < 0.01, ^***^ *p* < 0.001 | | | | | | | |

| Supplemental Table 1C. Weighted Multivariate Ordinary Squared Logistics Regression Predicting Psychological Distress Levels In the Past 30 Days Among the Total Population with Interaction Terms (Psychedelics * Family Income Level) | | | | | | | |
| --- | --- | --- | --- | --- | --- | --- | --- |
|  | Model 12 | Model 13 | Model 14 | Model 15 | Model 16 | Model 17 | Model 18 |
| Age | -0.555^***^ | -0.552^***^ | -0.552^***^ | -0.552^***^ | -0.552^***^ | -0.552^***^ | -0.553^***^ |
|  | (0.0111) | (0.0113) | (0.0113) | (0.0113) | (0.0113) | (0.0113) | (0.0113) |
|  |  |  |  |  |  |  |  |
| ^a^Women | 0.928^***^ | 0.917^***^ | 0.917^***^ | 0.917^***^ | 0.917^***^ | 0.917^***^ | 0.918^***^ |
|  | (0.0484) | (0.0480) | (0.0480) | (0.0480) | (0.0480) | (0.0481) | (0.0480) |
|  |  |  |  |  |  |  |  |
| ^b^Married | -0.400^***^ | -0.402^***^ | -0.403^***^ | -0.403^***^ | -0.404^***^ | -0.403^***^ | -0.402^***^ |
|  | (0.0621) | (0.0623) | (0.0624) | (0.0623) | (0.0622) | (0.0624) | (0.0624) |
|  |  |  |  |  |  |  |  |
| Divorced | -0.442^***^ | -0.458^***^ | -0.452^***^ | -0.454^***^ | -0.454^***^ | -0.452^***^ | -0.445^***^ |
|  | (0.120) | (0.121) | (0.121) | (0.121) | (0.121) | (0.121) | (0.121) |
|  |  |  |  |  |  |  |  |
| Widowed | 0.473^***^ | 0.470^***^ | 0.472^***^ | 0.472^***^ | 0.472^***^ | 0.471^***^ | 0.472^***^ |
|  | (0.0900) | (0.0902) | (0.0903) | (0.0903) | (0.0903) | (0.0904) | (0.0903) |
|  |  |  |  |  |  |  |  |
| ^c^Black | -0.415^***^ | -0.430^***^ | -0.426^***^ | -0.429^***^ | -0.428^***^ | -0.426^***^ | -0.420^***^ |
|  | (0.0713) | (0.0708) | (0.0710) | (0.0705) | (0.0705) | (0.0706) | (0.0709) |
|  |  |  |  |  |  |  |  |
| Hispanic | 0.00215 | -0.00468 | -0.00219 | -0.00342 | -0.00301 | -0.00202 | 0.000676 |
|  | (0.0783) | (0.0784) | (0.0782) | (0.0785) | (0.0785) | (0.0783) | (0.0784) |
|  |  |  |  |  |  |  |  |
| Asian | 0.312^**^ | 0.313^**^ | 0.313^**^ | 0.313^**^ | 0.313^**^ | 0.312^**^ | 0.311^**^ |
|  | (0.0996) | (0.0998) | (0.0997) | (0.0997) | (0.0997) | (0.0998) | (0.0996) |
|  |  |  |  |  |  |  |  |
| Native American | 0.774^**^ | 0.816^**^ | 0.816^**^ | 0.815^**^ | 0.816^**^ | 0.811^**^ | 0.818^**^ |
|  | (0.260) | (0.262) | (0.261) | (0.262) | (0.262) | (0.260) | (0.262) |
|  |  |  |  |  |  |  |  |
| Hawaiian/Pacific I | 0.280 | 0.268 | 0.273 | 0.272 | 0.272 | 0.274 | 0.275 |
|  | (0.433) | (0.433) | (0.432) | (0.432) | (0.432) | (0.432) | (0.432) |
|  |  |  |  |  |  |  |  |
| Multi Racial | 0.319^*^ | 0.331^*^ | 0.331^*^ | 0.331^*^ | 0.331^*^ | 0.331^*^ | 0.331^*^ |
|  | (0.158) | (0.157) | (0.157) | (0.157) | (0.157) | (0.157) | (0.157) |
|  |  |  |  |  |  |  |  |
| Religious Salience | -0.0427^***^ | -0.0429^***^ | -0.0430^***^ | -0.0430^***^ | -0.0429^***^ | -0.0430^***^ | -0.0431^***^ |
|  | (0.0114) | (0.0114) | (0.0114) | (0.0114) | (0.0114) | (0.0114) | (0.0114) |
|  |  |  |  |  |  |  |  |
| Religious Attendance | -0.126^***^ | -0.126^***^ | -0.126^***^ | -0.126^***^ | -0.126^***^ | -0.126^***^ | -0.126^***^ |
|  | (0.0150) | (0.0151) | (0.0151) | (0.0151) | (0.0151) | (0.0151) | (0.0150) |
|  |  |  |  |  |  |  |  |
| Cocaine | -0.0623 | -0.0460 | -0.0442 | -0.0450 | -0.0450 | -0.0442 | -0.0440 |
|  | (0.0691) | (0.0702) | (0.0706) | (0.0702) | (0.0701) | (0.0703) | (0.0702) |
|  |  |  |  |  |  |  |  |
| Stimulants | 0.609^***^ | 0.618^***^ | 0.618^***^ | 0.618^***^ | 0.619^***^ | 0.618^***^ | 0.616^***^ |
|  | (0.0690) | (0.0692) | (0.0690) | (0.0691) | (0.0692) | (0.0692) | (0.0691) |
|  |  |  |  |  |  |  |  |
| Sedatives | 0.879^***^ | 0.896^***^ | 0.897^***^ | 0.897^***^ | 0.897^***^ | 0.897^***^ | 0.899^***^ |
|  | (0.0722) | (0.0726) | (0.0726) | (0.0725) | (0.0726) | (0.0726) | (0.0726) |
|  |  |  |  |  |  |  |  |
| Tranquilizers | 1.061^***^ | 1.062^***^ | 1.061^***^ | 1.061^***^ | 1.061^***^ | 1.061^***^ | 1.060^***^ |
|  | (0.0545) | (0.0547) | (0.0546) | (0.0546) | (0.0546) | (0.0546) | (0.0546) |
|  |  |  |  |  |  |  |  |
| Heroine | 0.669^***^ | 0.735^***^ | 0.719^***^ | 0.724^***^ | 0.724^***^ | 0.715^***^ | 0.700^***^ |
|  | (0.149) | (0.148) | (0.147) | (0.147) | (0.147) | (0.146) | (0.148) |
|  |  |  |  |  |  |  |  |
| Pain Killers | 0.366^***^ | 0.373^***^ | 0.370^***^ | 0.371^***^ | 0.371^***^ | 0.370^***^ | 0.369^***^ |
|  | (0.0634) | (0.0634) | (0.0634) | (0.0633) | (0.0633) | (0.0634) | (0.0634) |
|  |  |  |  |  |  |  |  |
| Marijuana | 0.410^***^ | 0.405^***^ | 0.405^***^ | 0.405^***^ | 0.405^***^ | 0.405^***^ | 0.405^***^ |
|  | (0.0699) | (0.0700) | (0.0700) | (0.0700) | (0.0700) | (0.0701) | (0.0700) |
|  |  |  |  |  |  |  |  |
| PCP | -0.0463 | 0.0626 | 0.0554 | 0.0583 | 0.0578 | 0.0514 | 0.0451 |
|  | (0.182) | (0.181) | (0.181) | (0.181) | (0.181) | (0.180) | (0.181) |
|  |  |  |  |  |  |  |  |
| Inhalants | 0.546^***^ | 0.561^***^ | 0.564^***^ | 0.563^***^ | 0.563^***^ | 0.564^***^ | 0.567^***^ |
|  | (0.0745) | (0.0745) | (0.0746) | (0.0747) | (0.0747) | (0.0745) | (0.0745) |
|  |  |  |  |  |  |  |  |
| Tobacco | 0.451^***^ | 0.445^***^ | 0.446^***^ | 0.445^***^ | 0.445^***^ | 0.446^***^ | 0.447^***^ |
|  | (0.0549) | (0.0549) | (0.0550) | (0.0548) | (0.0548) | (0.0548) | (0.0549) |
|  |  |  |  |  |  |  |  |
| Age of 1^st^ Alcohol Use | -0.0249 | -0.0274 | -0.0262 | -0.0267 | -0.0266 | -0.0260 | -0.0248 |
|  | (0.0282) | (0.0281) | (0.0281) | (0.0281) | (0.0281) | (0.0281) | (0.0281) |
|  |  |  |  |  |  |  |  |
| Risk Behaviors | 0.655^***^ | 0.657^***^ | 0.656^***^ | 0.656^***^ | 0.657^***^ | 0.656^***^ | 0.656^***^ |
|  | (0.0310) | (0.0310) | (0.0310) | (0.0310) | (0.0310) | (0.0310) | (0.0310) |
|  |  |  |  |  |  |  |  |
| ^d^2009 | -0.0318 | -0.0290 | -0.0295 | -0.0295 | -0.0294 | -0.0299 | -0.0306 |
|  | (0.120) | (0.121) | (0.121) | (0.121) | (0.121) | (0.121) | (0.121) |
|  |  |  |  |  |  |  |  |
| 2010 | 0.0593 | 0.0602 | 0.0603 | 0.0601 | 0.0603 | 0.0596 | 0.0600 |
|  | (0.117) | (0.118) | (0.117) | (0.117) | (0.117) | (0.118) | (0.117) |
|  |  |  |  |  |  |  |  |
| 2011 | 0.0687 | 0.0717 | 0.0717 | 0.0722 | 0.0717 | 0.0709 | 0.0712 |
|  | (0.115) | (0.116) | (0.116) | (0.116) | (0.116) | (0.116) | (0.116) |
|  |  |  |  |  |  |  |  |
| 2012 | 0.167 | 0.171 | 0.171 | 0.171 | 0.171 | 0.170 | 0.169 |
|  | (0.118) | (0.119) | (0.119) | (0.119) | (0.119) | (0.119) | (0.119) |
|  |  |  |  |  |  |  |  |
| 2013 | 0.291^*^ | 0.292^*^ | 0.292^*^ | 0.291^*^ | 0.291^*^ | 0.291^*^ | 0.292^*^ |
|  | (0.142) | (0.142) | (0.142) | (0.142) | (0.142) | (0.142) | (0.141) |
|  |  |  |  |  |  |  |  |
| 2014 | 0.104 | 0.100 | 0.101 | 0.100 | 0.101 | 0.100 | 0.101 |
|  | (0.104) | (0.104) | (0.104) | (0.104) | (0.104) | (0.104) | (0.104) |
|  |  |  |  |  |  |  |  |
| 2015 | -0.317^**^ | -0.321^**^ | -0.319^**^ | -0.320^**^ | -0.320^**^ | -0.320^**^ | -0.320^**^ |
|  | (0.118) | (0.118) | (0.118) | (0.118) | (0.118) | (0.118) | (0.118) |
|  |  |  |  |  |  |  |  |
| 2016 | -0.203^†^ | -0.209^†^ | -0.208^†^ | -0.208^†^ | -0.208^†^ | -0.209^†^ | -0.208^†^ |
|  | (0.112) | (0.113) | (0.113) | (0.113) | (0.113) | (0.113) | (0.113) |
|  |  |  |  |  |  |  |  |
| 2017 | -0.0554 | -0.0642 | -0.0624 | -0.0627 | -0.0626 | -0.0628 | -0.0623 |
|  | (0.118) | (0.118) | (0.118) | (0.118) | (0.118) | (0.118) | (0.118) |
|  |  |  |  |  |  |  |  |
| 2018 | 0.241^†^ | 0.233^†^ | 0.235^†^ | 0.234^†^ | 0.234^†^ | 0.234^†^ | 0.235^†^ |
|  | (0.123) | (0.122) | (0.122) | (0.122) | (0.122) | (0.122) | (0.122) |
|  |  |  |  |  |  |  |  |
| 2019 | 0.567^***^ | 0.557^***^ | 0.559^***^ | 0.559^***^ | 0.559^***^ | 0.558^***^ | 0.558^***^ |
|  | (0.127) | (0.126) | (0.126) | (0.126) | (0.126) | (0.127) | (0.126) |
|  |  |  |  |  |  |  |  |
| MDMA | 0.186^*^ | -0.0495 | 0.184^*^ | 0.184^*^ | 0.184^*^ | 0.184^*^ | 0.182^*^ |
|  | (0.0845) | (0.177) | (0.0850) | (0.0850) | (0.0850) | (0.0851) | (0.0848) |
|  |  |  |  |  |  |  |  |
| LCPU | 0.113 |  |  |  |  |  |  |
|  | (0.164) |  |  |  |  |  |  |
|  |  |  |  |  |  |  |  |
| Educational Level | -0.419^***^ | -0.413^***^ | -0.414^***^ | -0.414^***^ | -0.414^***^ | -0.414^***^ | -0.416^***^ |
|  | (0.0251) | (0.0254) | (0.0255) | (0.0253) | (0.0253) | (0.0253) | (0.0255) |
|  |  |  |  |  |  |  |  |
| Family Income | -0.354^***^ | -0.371^***^ | -0.364^***^ | -0.366^***^ | -0.366^***^ | -0.363^***^ | -0.356^***^ |
|  | (0.0128) | (0.0130) | (0.0130) | (0.0128) | (0.0128) | (0.0123) | (0.0129) |
|  |  |  |  |  |  |  |  |
| LCPU * Family Income | -0.0570^†^ |  |  |  |  |  |  |
|  | (0.0293) |  |  |  |  |  |  |
|  |  |  |  |  |  |  |  |
| Psilocybin |  | -0.282^**^ | -0.205 | -0.278^**^ | -0.278^**^ | -0.279^**^ | -0.275^**^ |
|  |  | (0.0847) | (0.179) | (0.0851) | (0.0851) | (0.0851) | (0.0849) |
|  |  |  |  |  |  |  |  |
| DMT |  | 0.563 | 0.565 | -0.626 | 0.563 | 0.580 | 0.571 |
|  |  | (0.598) | (0.597) | (1.203) | (0.601) | (0.598) | (0.596) |
|  |  |  |  |  |  |  |  |
| Ayahuasca |  | 1.311 | 1.336 | 1.377 | 1.776 | 1.340 | 1.344 |
|  |  | (1.693) | (1.703) | (1.706) | (5.853) | (1.706) | (1.705) |
|  |  |  |  |  |  |  |  |
| Peyote/Mescaline |  | -0.446^***^ | -0.448^***^ | -0.450^***^ | -0.447^***^ | -0.223 | -0.448^***^ |
|  |  | (0.126) | (0.126) | (0.126) | (0.126) | (0.326) | (0.126) |
|  |  |  |  |  |  |  |  |
| LSD |  | 0.126 | 0.126 | 0.127 | 0.126 | 0.128 | 0.459^*^ |
|  |  | (0.0827) | (0.0827) | (0.0827) | (0.0827) | (0.0824) | (0.188) |
|  |  |  |  |  |  |  |  |
| MDMA * Family Income |  | 0.0488 |  |  |  |  |  |
|  |  | (0.0305) |  |  |  |  |  |
|  |  |  |  |  |  |  |  |
| Psilocybin * Family Income |  |  | -0.0146 |  |  |  |  |
|  |  |  | (0.0353) |  |  |  |  |
|  |  |  |  |  |  |  |  |
| DMT * Family Income |  |  |  | 0.249 |  |  |  |
|  |  |  |  | (0.221) |  |  |  |
|  |  |  |  |  |  |  |  |
| Ayahuasca * Family Income |  |  |  |  | -0.0828 |  |  |
|  |  |  |  |  | (0.959) |  |  |
|  |  |  |  |  |  |  |  |
| Peyote/Mescaline * Family Income |  |  |  |  |  | -0.0459 |  |
|  |  |  |  |  |  | (0.0557) |  |
|  |  |  |  |  |  |  |  |
| LSD * Family Income |  |  |  |  |  |  | -0.0672^*^ |
|  |  |  |  |  |  |  | (0.0339) |
|  |  |  |  |  |  |  |  |
| Constant | 18.22^***^ | 18.26^***^ | 18.22^***^ | 18.23^***^ | 18.23^***^ | 18.22^***^ | 18.19^***^ |
|  | (0.226) | (0.229) | (0.226) | (0.231) | (0.230) | (0.229) | (0.230) |
| Observations | 149002 | 149002 | 149002 | 149002 | 149002 | 149002 | 149002 |
| *R*^2^ | 0.148 | 0.148 | 0.148 | 0.148 | 0.148 | 0.148 | 0.148 |
| Source: 2008-2019 National Survey of Drug Use and Health, N=458,372  Standard errors in parentheses  ^a^ Men serve as the references category  ^b^ Single Serves as the reference category for marital statuses  ^c^ White, Non-Hispanic Serves as the reference category for race/ethnicity  ^d^ 2008 serves as the reference category for the year survey was taken  ^†^ *p* < 0.1, ^*^ *p* < 0.05, ^**^ *p* < 0.01, ^***^ *p* < 0.001 | | | | | | | |

| Supplemental Table 2a. Weighted Multivariate Ordinary Squared Logistics Regression Predicting Psychological Distress Levels In the Past 30 Days Among White People | | | | |
| --- | --- | --- | --- | --- |
|  | Model 1 | Model 2 | Model 3 | Model 4 |
| Age | -0.667^***^ | -0.619^***^ | -0.664^***^ | -0.616^***^ |
|  | (0.0143) | (0.0144) | (0.0144) | (0.0145) |
|  |  |  |  |  |
| ^a^Women | 0.974^***^ | 0.949^***^ | 0.959^***^ | 0.935^***^ |
|  | (0.0600) | (0.0582) | (0.0597) | (0.0577) |
|  |  |  |  |  |
| ^b^Married | -0.835^***^ | -0.309^***^ | -0.843^***^ | -0.314^***^ |
|  | (0.0701) | (0.0735) | (0.0704) | (0.0735) |
|  |  |  |  |  |
| Divorced | -0.0726 | -0.453^***^ | -0.0833 | -0.458^***^ |
|  | (0.133) | (0.132) | (0.133) | (0.132) |
|  |  |  |  |  |
| Widowed | 0.881^***^ | 0.698^***^ | 0.867^***^ | 0.692^***^ |
|  | (0.110) | (0.107) | (0.110) | (0.108) |
|  |  |  |  |  |
| Religious Salience | 0.0161 | -0.0406^**^ | 0.0146 | -0.0413^**^ |
|  | (0.0130) | (0.0131) | (0.0129) | (0.0130) |
|  |  |  |  |  |
| Religious Attendance | -0.200^***^ | -0.123^***^ | -0.200^***^ | -0.123^***^ |
|  | (0.0197) | (0.0193) | (0.0197) | (0.0193) |
|  |  |  |  |  |
| Cocaine | 0.0224 | -0.0591 | 0.0373 | -0.0374 |
|  | (0.0841) | (0.0820) | (0.0840) | (0.0818) |
|  |  |  |  |  |
| Stimulants | 0.530^***^ | 0.540^***^ | 0.541^***^ | 0.549^***^ |
|  | (0.0833) | (0.0794) | (0.0832) | (0.0793) |
|  |  |  |  |  |
| Sedatives | 0.832^***^ | 0.863^***^ | 0.849^***^ | 0.882^***^ |
|  | (0.0835) | (0.0815) | (0.0837) | (0.0819) |
|  |  |  |  |  |
| Tranquilizers | 1.073^***^ | 1.043^***^ | 1.073^***^ | 1.042^***^ |
|  | (0.0689) | (0.0678) | (0.0692) | (0.0680) |
|  |  |  |  |  |
| Heroine | 1.301^***^ | 0.903^***^ | 1.322^***^ | 0.932^***^ |
|  | (0.176) | (0.171) | (0.173) | (0.168) |
|  |  |  |  |  |
| Pain Killers | 0.353^***^ | 0.329^***^ | 0.358^***^ | 0.333^***^ |
|  | (0.0690) | (0.0699) | (0.0689) | (0.0699) |
|  |  |  |  |  |
| Marijuana | 0.429^***^ | 0.524^***^ | 0.423^***^ | 0.519^***^ |
|  | (0.0765) | (0.0753) | (0.0763) | (0.0751) |
|  |  |  |  |  |
| PCP | 0.175 | -0.0703 | 0.264 | 0.0465 |
|  | (0.208) | (0.202) | (0.211) | (0.203) |
|  |  |  |  |  |
| Inhalants | 0.506^***^ | 0.576^***^ | 0.527^***^ | 0.595^***^ |
|  | (0.0856) | (0.0853) | (0.0851) | (0.0848) |
|  |  |  |  |  |
| Tobacco | 0.576^***^ | 0.353^***^ | 0.571^***^ | 0.350^***^ |
|  | (0.0646) | (0.0657) | (0.0642) | (0.0653) |
|  |  |  |  |  |
| Age of 1^st^ Alcohol Use | 0.0946^**^ | -0.00795 | 0.0954^**^ | -0.00711 |
|  | (0.0324) | (0.0317) | (0.0324) | (0.0316) |
|  |  |  |  |  |
| Risk Behaviors | 0.465^***^ | 0.579^***^ | 0.469^***^ | 0.581^***^ |
|  | (0.0382) | (0.0379) | (0.0383) | (0.0379) |
|  |  |  |  |  |
| ^c^2009 | -0.0531 | -0.0831 | -0.0514 | -0.0813 |
|  | (0.144) | (0.145) | (0.144) | (0.146) |
|  |  |  |  |  |
| 2010 | 0.0966 | 0.0495 | 0.0984 | 0.0509 |
|  | (0.134) | (0.130) | (0.135) | (0.131) |
|  |  |  |  |  |
| 2011 | 0.114 | 0.0672 | 0.119 | 0.0703 |
|  | (0.144) | (0.142) | (0.145) | (0.143) |
|  |  |  |  |  |
| 2012 | 0.0710 | 0.0518 | 0.0768 | 0.0561 |
|  | (0.141) | (0.137) | (0.142) | (0.138) |
|  |  |  |  |  |
| 2013 | 0.170 | 0.225 | 0.172 | 0.225 |
|  | (0.172) | (0.176) | (0.172) | (0.176) |
|  |  |  |  |  |
| 2014 | 0.0388 | 0.0670 | 0.0404 | 0.0661 |
|  | (0.127) | (0.127) | (0.127) | (0.127) |
|  |  |  |  |  |
| 2015 | -0.538^***^ | -0.440^**^ | -0.539^***^ | -0.441^**^ |
|  | (0.149) | (0.148) | (0.149) | (0.147) |
|  |  |  |  |  |
| 2016 | -0.316^*^ | -0.222^†^ | -0.324^*^ | -0.229^†^ |
|  | (0.138) | (0.133) | (0.138) | (0.133) |
|  |  |  |  |  |
| 2017 | -0.283^†^ | -0.130 | -0.292^*^ | -0.139 |
|  | (0.147) | (0.144) | (0.147) | (0.144) |
|  |  |  |  |  |
| 2018 | -0.0387 | 0.139 | -0.0445 | 0.133 |
|  | (0.144) | (0.143) | (0.145) | (0.144) |
|  |  |  |  |  |
| 2019 | 0.211 | 0.410^**^ | 0.202 | 0.400^**^ |
|  | (0.141) | (0.138) | (0.141) | (0.138) |
|  |  |  |  |  |
| MDMA | 0.0170 | 0.0603 | 0.0149 | 0.0489 |
|  | (0.101) | (0.0976) | (0.105) | (0.101) |
|  |  |  |  |  |
| LCPU | -0.120 | -0.147^†^ |  |  |
|  | (0.0798) | (0.0796) |  |  |
|  |  |  |  |  |
| Educational Level |  | -0.438^***^ |  | -0.431^***^ |
|  |  | (0.0279) |  | (0.0284) |
|  |  |  |  |  |
| Family Income |  | -0.374^***^ |  | -0.375^***^ |
|  |  | (0.0171) |  | (0.0170) |
|  |  |  |  |  |
| Psilocybin |  |  | -0.437^***^ | -0.346^***^ |
|  |  |  | (0.0980) | (0.0970) |
|  |  |  |  |  |
| DMT |  |  | 0.264 | 0.490 |
|  |  |  | (0.645) | (0.625) |
|  |  |  |  |  |
| Ayahuasca |  |  | -0.0240 | 0.243 |
|  |  |  | (1.920) | (1.955) |
|  |  |  |  |  |
| Peyote/Mescaline |  |  | -0.503^**^ | -0.535^***^ |
|  |  |  | (0.152) | (0.148) |
|  |  |  |  |  |
| LSD |  |  | 0.387^***^ | 0.270 |
|  |  |  | (0.102) | (0.104) |
|  |  |  |  |  |
| Constant | 16.80^***^ | 19.38^***^ | 16.77^***^ | 19.33^***^ |
|  | (0.239) | (0.258) | (0.241) | (0.261) |
| Observations | 97184 | 97184 | 97184 | 97184 |
| *R*^2^ | 0.138 | 0.159 | 0.139 | 0.160 |
| Source: 2008-2019 National Survey of Drug Use and Health, N=458,372  Standard errors in parentheses  ^a^ Men serve as the references category  ^b^ Single Serves as the reference category for marital statuses  ^c^ 2008 serves as the reference category for the year survey was taken  ^†^ *p* < 0.1, ^*^ *p* < 0.05, ^**^ *p* < 0.01, ^***^ *p* < 0.001 | | | | |

| Supplemental Table 2b. Weighted Multivariate Ordinary Squared Logistics Regression Predicting Psychological Distress Levels In the Past 30 Days Among White People with interaction terms (Psychedelics * Educational Level) | | | | | | | |
| --- | --- | --- | --- | --- | --- | --- | --- |
|  | Model 5 | Model 6 | Model 7 | Model 8 | Model 9 | Model 10 | Model 11 |
| Age | -0.617^***^ | -0.615^***^ | -0.615^***^ | -0.616^***^ | -0.616^***^ | -0.616^***^ | -0.615^***^ |
|  | (0.0144) | (0.0145) | (0.0145) | (0.0145) | (0.0145) | (0.0145) | (0.0145) |
|  |  |  |  |  |  |  |  |
| ^a^Women | 0.953^***^ | 0.936^***^ | 0.935^***^ | 0.935^***^ | 0.935^***^ | 0.935^***^ | 0.939^***^ |
|  | (0.0580) | (0.0578) | (0.0574) | (0.0577) | (0.0577) | (0.0576) | (0.0575) |
|  |  |  |  |  |  |  |  |
| ^b^Married | -0.309^***^ | -0.314^***^ | -0.313^***^ | -0.314^***^ | -0.313^***^ | -0.313^***^ | -0.313^***^ |
|  | (0.0736) | (0.0736) | (0.0735) | (0.0735) | (0.0735) | (0.0735) | (0.0737) |
|  |  |  |  |  |  |  |  |
| Divorced | -0.442^**^ | -0.456^***^ | -0.457^***^ | -0.458^***^ | -0.458^***^ | -0.457^***^ | -0.446^**^ |
|  | (0.132) | (0.132) | (0.132) | (0.132) | (0.132) | (0.132) | (0.132) |
|  |  |  |  |  |  |  |  |
| Widowed | 0.697^***^ | 0.690^***^ | 0.692^***^ | 0.692^***^ | 0.692^***^ | 0.692^***^ | 0.691^***^ |
|  | (0.107) | (0.108) | (0.108) | (0.108) | (0.108) | (0.108) | (0.108) |
|  |  |  |  |  |  |  |  |
| Religious Salience | -0.0408^**^ | -0.0414^**^ | -0.0413^**^ | -0.0413^**^ | -0.0413^**^ | -0.0413^**^ | -0.0414^**^ |
|  | (0.0131) | (0.0130) | (0.0130) | (0.0130) | (0.0130) | (0.0130) | (0.0130) |
|  |  |  |  |  |  |  |  |
| Religious Attendance | -0.125^***^ | -0.124^***^ | -0.123^***^ | -0.123^***^ | -0.123^***^ | -0.123^***^ | -0.125^***^ |
|  | (0.0193) | (0.0193) | (0.0193) | (0.0193) | (0.0193) | (0.0193) | (0.0192) |
|  |  |  |  |  |  |  |  |
| Cocaine | -0.0609 | -0.0363 | -0.0372 | -0.0374 | -0.0376 | -0.0372 | -0.0405 |
|  | (0.0818) | (0.0819) | (0.0819) | (0.0817) | (0.0818) | (0.0817) | (0.0814) |
|  |  |  |  |  |  |  |  |
| Stimulants | 0.542^***^ | 0.551^***^ | 0.550^***^ | 0.549^***^ | 0.549^***^ | 0.549^***^ | 0.551^***^ |
|  | (0.0794) | (0.0795) | (0.0794) | (0.0793) | (0.0793) | (0.0793) | (0.0792) |
|  |  |  |  |  |  |  |  |
| Sedatives | 0.867^***^ | 0.883^***^ | 0.883^***^ | 0.882^***^ | 0.882^***^ | 0.883^***^ | 0.887^***^ |
|  | (0.0815) | (0.0819) | (0.0818) | (0.0819) | (0.0819) | (0.0818) | (0.0818) |
|  |  |  |  |  |  |  |  |
| Tranquilizers | 1.041^***^ | 1.040^***^ | 1.041^***^ | 1.042^***^ | 1.042^***^ | 1.041^***^ | 1.039^***^ |
|  | (0.0677) | (0.0680) | (0.0681) | (0.0680) | (0.0680) | (0.0680) | (0.0679) |
|  |  |  |  |  |  |  |  |
| Heroine | 0.870^***^ | 0.916^***^ | 0.929^***^ | 0.932^***^ | 0.932^***^ | 0.931^***^ | 0.899^***^ |
|  | (0.170) | (0.170) | (0.167) | (0.168) | (0.168) | (0.168) | (0.168) |
|  |  |  |  |  |  |  |  |
| Pain Killers | 0.322^***^ | 0.330^***^ | 0.332^***^ | 0.333^***^ | 0.333^***^ | 0.333^***^ | 0.326^***^ |
|  | (0.0698) | (0.0703) | (0.0699) | (0.0699) | (0.0699) | (0.0700) | (0.0700) |
|  |  |  |  |  |  |  |  |
| Marijuana | 0.523^***^ | 0.519^***^ | 0.519^***^ | 0.519^***^ | 0.519^***^ | 0.519^***^ | 0.517^***^ |
|  | (0.0753) | (0.0751) | (0.0751) | (0.0751) | (0.0751) | (0.0750) | (0.0750) |
|  |  |  |  |  |  |  |  |
| PCP | -0.107 | 0.0370 | 0.0436 | 0.0468 | 0.0468 | 0.0441 | 0.00421 |
|  | (0.203) | (0.204) | (0.203) | (0.203) | (0.203) | (0.203) | (0.205) |
|  |  |  |  |  |  |  |  |
| Inhalants | 0.583^***^ | 0.597^***^ | 0.595^***^ | 0.595^***^ | 0.595^***^ | 0.595^***^ | 0.601^***^ |
|  | (0.0852) | (0.0849) | (0.0847) | (0.0849) | (0.0850) | (0.0850) | (0.0848) |
|  |  |  |  |  |  |  |  |
| Tobacco | 0.359^***^ | 0.351^***^ | 0.350^***^ | 0.350^***^ | 0.350^***^ | 0.350^***^ | 0.355^***^ |
|  | (0.0656) | (0.0654) | (0.0654) | (0.0653) | (0.0653) | (0.0650) | (0.0653) |
|  |  |  |  |  |  |  |  |
| Age of 1^st^ Alcohol Use | -0.00466 | -0.00638 | -0.00688 | -0.00711 | -0.00709 | -0.00701 | -0.00418 |
|  | (0.0320) | (0.0317) | (0.0319) | (0.0316) | (0.0316) | (0.0316) | (0.0318) |
|  |  |  |  |  |  |  |  |
| Risk Behaviors | 0.578^***^ | 0.580^***^ | 0.581^***^ | 0.581^***^ | 0.581^***^ | 0.581^***^ | 0.579^***^ |
|  | (0.0379) | (0.0380) | (0.0380) | (0.0379) | (0.0379) | (0.0380) | (0.0380) |
|  |  |  |  |  |  |  |  |
| ^c^2009 | -0.0835 | -0.0821 | -0.0814 | -0.0813 | -0.0812 | -0.0813 | -0.0807 |
|  | (0.145) | (0.146) | (0.146) | (0.146) | (0.146) | (0.146) | (0.145) |
|  |  |  |  |  |  |  |  |
| 2010 | 0.0519 | 0.0512 | 0.0510 | 0.0509 | 0.0512 | 0.0510 | 0.0541 |
|  | (0.130) | (0.131) | (0.131) | (0.131) | (0.131) | (0.131) | (0.131) |
|  |  |  |  |  |  |  |  |
| 2011 | 0.0666 | 0.0693 | 0.0701 | 0.0702 | 0.0704 | 0.0702 | 0.0711 |
|  | (0.142) | (0.143) | (0.143) | (0.143) | (0.143) | (0.143) | (0.143) |
|  |  |  |  |  |  |  |  |
| 2012 | 0.0522 | 0.0560 | 0.0559 | 0.0563 | 0.0562 | 0.0562 | 0.0568 |
|  | (0.138) | (0.139) | (0.139) | (0.138) | (0.138) | (0.138) | (0.139) |
|  |  |  |  |  |  |  |  |
| 2013 | 0.227 | 0.224 | 0.225 | 0.225 | 0.225 | 0.225 | 0.229 |
|  | (0.176) | (0.176) | (0.176) | (0.176) | (0.176) | (0.176) | (0.176) |
|  |  |  |  |  |  |  |  |
| 2014 | 0.0663 | 0.0658 | 0.0659 | 0.0660 | 0.0660 | 0.0661 | 0.0661 |
|  | (0.127) | (0.127) | (0.127) | (0.127) | (0.127) | (0.127) | (0.127) |
|  |  |  |  |  |  |  |  |
| 2015 | -0.436^**^ | -0.440^**^ | -0.441^**^ | -0.441^**^ | -0.441^**^ | -0.441^**^ | -0.436^**^ |
|  | (0.148) | (0.147) | (0.147) | (0.147) | (0.147) | (0.147) | (0.147) |
|  |  |  |  |  |  |  |  |
| 2016 | -0.218 | -0.228^†^ | -0.229^†^ | -0.229^†^ | -0.229^†^ | -0.229^†^ | -0.224^†^ |
|  | (0.133) | (0.133) | (0.133) | (0.133) | (0.133) | (0.133) | (0.133) |
|  |  |  |  |  |  |  |  |
| 2017 | -0.126 | -0.137 | -0.139 | -0.139 | -0.139 | -0.139 | -0.134 |
|  | (0.144) | (0.144) | (0.144) | (0.144) | (0.144) | (0.144) | (0.144) |
|  |  |  |  |  |  |  |  |
| 2018 | 0.142 | 0.133 | 0.133 | 0.133 | 0.133 | 0.133 | 0.137 |
|  | (0.143) | (0.144) | (0.144) | (0.144) | (0.144) | (0.144) | (0.143) |
|  |  |  |  |  |  |  |  |
| 2019 | 0.414^**^ | 0.402^**^ | 0.400^**^ | 0.400^**^ | 0.400^**^ | 0.400^**^ | 0.405^**^ |
|  | (0.137) | (0.138) | (0.137) | (0.138) | (0.138) | (0.138) | (0.137) |
|  |  |  |  |  |  |  |  |
| MDMA | 0.0654 | 0.320 | 0.0489 | 0.0488 | 0.0487 | 0.0487 | 0.0536 |
|  | (0.0971) | (0.282) | (0.100) | (0.100) | (0.100) | (0.100) | (0.100) |
|  |  |  |  |  |  |  |  |
| LCPU | 0.355^*^ |  |  |  |  |  |  |
|  | (0.172) |  |  |  |  |  |  |
|  |  |  |  |  |  |  |  |
| Educational Level | -0.400^***^ | -0.421^***^ | -0.428^***^ | -0.431^***^ | -0.431^***^ | -0.430^***^ | -0.396^***^ |
|  | (0.0296) | (0.0296) | (0.0292) | (0.0286) | (0.0284) | (0.0296) | (0.0295) |
|  |  |  |  |  |  |  |  |
| Family Income | -0.375^***^ | -0.376^***^ | -0.375^***^ | -0.375^***^ | -0.375^***^ | -0.375^***^ | -0.376^***^ |
|  | (0.0171) | (0.0170) | (0.0170) | (0.0170) | (0.0170) | (0.0171) | (0.0170) |
|  |  |  |  |  |  |  |  |
| LCPU * Educational Level | -0.167^**^ |  |  |  |  |  |  |
|  | (0.0528) |  |  |  |  |  |  |
|  |  |  |  |  |  |  |  |
| Psilocybin |  | -0.343^***^ | -0.296 | -0.346^***^ | -0.346^***^ | -0.346^***^ | -0.331^***^ |
|  |  | (0.0972) | (0.243) | (0.0970) | (0.0970) | (0.0970) | (0.0973) |
|  |  |  |  |  |  |  |  |
| DMT |  | 0.502 | 0.492 | 0.215 | 0.498 | 0.492 | 0.523 |
|  |  | (0.620) | (0.625) | (1.312) | (0.626) | (0.626) | (0.618) |
|  |  |  |  |  |  |  |  |
| Ayahuasca |  | 0.259 | 0.246 | 0.248 | -2.391 | 0.249 | 0.278 |
|  |  | (1.969) | (1.958) | (1.956) | (4.580) | (1.966) | (1.970) |
|  |  |  |  |  |  |  |  |
| Peyote/Mescaline |  | -0.536^***^ | -0.536^***^ | -0.535^***^ | -0.535^***^ | -0.481 | -0.530^***^ |
|  |  | (0.148) | (0.147) | (0.148) | (0.148) | (0.443) | (0.147) |
|  |  |  |  |  |  |  |  |
| LSD |  | 0.269^*^ | 0.270^*^ | 0.270^*^ | 0.270^*^ | 0.270^*^ | 0.856^***^ |
|  |  | (0.104) | (0.104) | (0.104) | (0.104) | (0.103) | (0.185) |
|  |  |  |  |  |  |  |  |
| MDMA * Educational Level |  | -0.0916 |  |  |  |  |  |
|  |  | (0.0843) |  |  |  |  |  |
|  |  |  |  |  |  |  |  |
| Psilocybin * Educational Level |  |  | -0.0163 |  |  |  |  |
|  |  |  | (0.0704) |  |  |  |  |
|  |  |  |  |  |  |  |  |
| DMT * Educational Level |  |  |  | 0.0904 |  |  |  |
|  |  |  |  | (0.450) |  |  |  |
|  |  |  |  |  |  |  |  |
| Ayahuasca * Educational Level |  |  |  |  | 0.796 |  |  |
|  |  |  |  |  | (1.548) |  |  |
|  |  |  |  |  |  |  |  |
| Peyote/Mescaline * Educational Level |  |  |  |  |  | -0.0186 |  |
|  |  |  |  |  |  | (0.126) |  |
|  |  |  |  |  |  |  |  |
| LSD * Educational Level |  |  |  |  |  |  | -0.201^**^ |
|  |  |  |  |  |  |  | (0.0626) |
|  |  |  |  |  |  |  |  |
| Constant | 19.25^***^ | 19.29^***^ | 19.32^***^ | 19.33^***^ | 19.33^***^ | 19.33^***^ | 19.21^***^ |
|  | (0.262) | (0.266) | (0.261) | (0.261) | (0.261) | (0.260) | (0.261) |
| Observations | 97184 | 97184 | 97184 | 97184 | 97184 | 97184 | 97184 |
| *R*^2^ | 0.160 | 0.160 | 0.160 | 0.160 | 0.160 | 0.160 | 0.160 |
| Source: 2008-2019 National Survey of Drug Use and Health, N=458,372  Standard errors in parentheses  ^a^ Men serve as the references category  ^b^ Single Serves as the reference category for marital statuses  ^c^ 2008 serves as the reference category for the year survey was taken  ^†^ *p* < 0.1, ^*^ *p* < 0.05, ^**^ *p* < 0.01, ^***^ *p* < 0.001 | | | | | | | |

| Supplemental Table 2c. Weighted Multivariate Ordinary Squared Logistics Regression Predicting Psychological Distress Levels In the Past 30 Days Among White People with interaction terms (Psychedelics * Family Income Level) | | | | | | | |
| --- | --- | --- | --- | --- | --- | --- | --- |
|  | Model 12 | Model 13 | Model 14 | Model 15 | Model 16 | Model 17 | Model 18 |
| Age | -0.619^***^ | -0.616^***^ | -0.616^***^ | -0.616^***^ | -0.616^***^ | -0.616^***^ | -0.616^***^ |
|  | (0.0143) | (0.0145) | (0.0145) | (0.0145) | (0.0145) | (0.0144) | (0.0144) |
|  |  |  |  |  |  |  |  |
| ^a^Women | 0.952^***^ | 0.935^***^ | 0.935^***^ | 0.935^***^ | 0.935^***^ | 0.936^***^ | 0.937^***^ |
|  | (0.0581) | (0.0577) | (0.0577) | (0.0577) | (0.0577) | (0.0576) | (0.0576) |
|  |  |  |  |  |  |  |  |
| ^b^Married | -0.307^***^ | -0.312^***^ | -0.313^***^ | -0.313^***^ | -0.313^***^ | -0.313^***^ | -0.312^***^ |
|  | (0.0736) | (0.0736) | (0.0736) | (0.0737) | (0.0735) | (0.0736) | (0.0737) |
|  |  |  |  |  |  |  |  |
| Divorced | -0.440^**^ | -0.464^***^ | -0.456^***^ | -0.458^***^ | -0.458^***^ | -0.456^***^ | -0.446^**^ |
|  | (0.132) | (0.133) | (0.133) | (0.132) | (0.132) | (0.132) | (0.132) |
|  |  |  |  |  |  |  |  |
| Widowed | 0.701^***^ | 0.688^***^ | 0.692^***^ | 0.691^***^ | 0.692^***^ | 0.691^***^ | 0.694^***^ |
|  | (0.107) | (0.108) | (0.108) | (0.108) | (0.108) | (0.108) | (0.108) |
|  |  |  |  |  |  |  |  |
| Religious Salience | -0.0407^**^ | -0.0413^**^ | -0.0413^**^ | -0.0414^**^ | -0.0413^**^ | -0.0413^**^ | -0.0414^**^ |
|  | (0.0131) | (0.0130) | (0.0130) | (0.0130) | (0.0130) | (0.0130) | (0.0130) |
|  |  |  |  |  |  |  |  |
| Religious Attendance | -0.124^***^ | -0.123^***^ | -0.123^***^ | -0.123^***^ | -0.123^***^ | -0.123^***^ | -0.124^***^ |
|  | (0.0193) | (0.0193) | (0.0193) | (0.0193) | (0.0193) | (0.0193) | (0.0192) |
|  |  |  |  |  |  |  |  |
| Cocaine | -0.0577 | -0.0382 | -0.0368 | -0.0373 | -0.0375 | -0.0368 | -0.0370 |
|  | (0.0822) | (0.0817) | (0.0823) | (0.0819) | (0.0818) | (0.0818) | (0.0818) |
|  |  |  |  |  |  |  |  |
| Stimulants | 0.537^***^ | 0.550^***^ | 0.549^***^ | 0.549^***^ | 0.549^***^ | 0.549^***^ | 0.547^***^ |
|  | (0.0793) | (0.0792) | (0.0791) | (0.0792) | (0.0793) | (0.0792) | (0.0792) |
|  |  |  |  |  |  |  |  |
| Sedatives | 0.864^***^ | 0.881^***^ | 0.882^***^ | 0.882^***^ | 0.882^***^ | 0.883^***^ | 0.884^***^ |
|  | (0.0815) | (0.0820) | (0.0819) | (0.0819) | (0.0819) | (0.0819) | (0.0819) |
|  |  |  |  |  |  |  |  |
| Tranquilizers | 1.042^***^ | 1.042^***^ | 1.042^***^ | 1.042^***^ | 1.041^***^ | 1.042^***^ | 1.041^***^ |
|  | (0.0678) | (0.0681) | (0.0680) | (0.0681) | (0.0680) | (0.0680) | (0.0680) |
|  |  |  |  |  |  |  |  |
| Heroine | 0.879^***^ | 0.948^***^ | 0.929^***^ | 0.933^***^ | 0.932^***^ | 0.927^***^ | 0.909^***^ |
|  | (0.171) | (0.169) | (0.170) | (0.168) | (0.168) | (0.168) | (0.169) |
|  |  |  |  |  |  |  |  |
| Pain Killers | 0.326^***^ | 0.335^***^ | 0.333^***^ | 0.333^***^ | 0.333^***^ | 0.333^***^ | 0.331^***^ |
|  | (0.0699) | (0.0699) | (0.0699) | (0.0698) | (0.0699) | (0.0699) | (0.0699) |
|  |  |  |  |  |  |  |  |
| Marijuana | 0.524^***^ | 0.519^***^ | 0.519^***^ | 0.519^***^ | 0.519^***^ | 0.519^***^ | 0.519^***^ |
|  | (0.0753) | (0.0750) | (0.0751) | (0.0750) | (0.0751) | (0.0751) | (0.0751) |
|  |  |  |  |  |  |  |  |
| PCP | -0.0831 | 0.0506 | 0.0448 | 0.0468 | 0.0465 | 0.0414 | 0.0353 |
|  | (0.201) | (0.203) | (0.202) | (0.203) | (0.203) | (0.201) | (0.203) |
|  |  |  |  |  |  |  |  |
| Inhalants | 0.580^***^ | 0.592^***^ | 0.595^***^ | 0.595^***^ | 0.595^***^ | 0.595^***^ | 0.598^***^ |
|  | (0.0851) | (0.0847) | (0.0846) | (0.0849) | (0.0849) | (0.0847) | (0.0846) |
|  |  |  |  |  |  |  |  |
| Tobacco | 0.356^***^ | 0.349^***^ | 0.350^***^ | 0.350^***^ | 0.350^***^ | 0.350^***^ | 0.352^***^ |
|  | (0.0657) | (0.0654) | (0.0654) | (0.0653) | (0.0652) | (0.0652) | (0.0654) |
|  |  |  |  |  |  |  |  |
| Age of 1^st^ Alcohol Use | -0.00526 | -0.00834 | -0.00679 | -0.00735 | -0.00712 | -0.00649 | -0.00466 |
|  | (0.0317) | (0.0317) | (0.0317) | (0.0316) | (0.0316) | (0.0316) | (0.0316) |
|  |  |  |  |  |  |  |  |
| Risk Behaviors | 0.579^***^ | 0.582^***^ | 0.581^***^ | 0.581^***^ | 0.581^***^ | 0.581^***^ | 0.581^***^ |
|  | (0.0379) | (0.0379) | (0.0379) | (0.0379) | (0.0379) | (0.0379) | (0.0379) |
|  |  |  |  |  |  |  |  |
| ^c^2009 | -0.0843 | -0.0809 | -0.0814 | -0.0816 | -0.0812 | -0.0816 | -0.0822 |
|  | (0.145) | (0.146) | (0.146) | (0.146) | (0.146) | (0.146) | (0.146) |
|  |  |  |  |  |  |  |  |
| 2010 | 0.0492 | 0.0514 | 0.0508 | 0.0507 | 0.0510 | 0.0501 | 0.0506 |
|  | (0.130) | (0.131) | (0.131) | (0.131) | (0.131) | (0.131) | (0.131) |
|  |  |  |  |  |  |  |  |
| 2011 | 0.0672 | 0.0701 | 0.0704 | 0.0713 | 0.0704 | 0.0699 | 0.0701 |
|  | (0.142) | (0.143) | (0.143) | (0.143) | (0.143) | (0.143) | (0.143) |
|  |  |  |  |  |  |  |  |
| 2012 | 0.0515 | 0.0563 | 0.0560 | 0.0564 | 0.0562 | 0.0554 | 0.0550 |
|  | (0.137) | (0.138) | (0.139) | (0.138) | (0.139) | (0.139) | (0.139) |
|  |  |  |  |  |  |  |  |
| 2013 | 0.226 | 0.226 | 0.225 | 0.225 | 0.225 | 0.225 | 0.226 |
|  | (0.176) | (0.176) | (0.176) | (0.176) | (0.176) | (0.176) | (0.176) |
|  |  |  |  |  |  |  |  |
| 2014 | 0.0673 | 0.0653 | 0.0661 | 0.0658 | 0.0661 | 0.0655 | 0.0660 |
|  | (0.127) | (0.127) | (0.127) | (0.127) | (0.127) | (0.127) | (0.127) |
|  |  |  |  |  |  |  |  |
| 2015 | -0.438^**^ | -0.442^**^ | -0.441^**^ | -0.441^**^ | -0.441^**^ | -0.442^**^ | -0.441^**^ |
|  | (0.148) | (0.147) | (0.147) | (0.147) | (0.147) | (0.147) | (0.147) |
|  |  |  |  |  |  |  |  |
| 2016 | -0.221 | -0.230^†^ | -0.229^†^ | -0.229^†^ | -0.229^†^ | -0.229^†^ | -0.229^†^ |
|  | (0.133) | (0.133) | (0.133) | (0.133) | (0.133) | (0.133) | (0.133) |
|  |  |  |  |  |  |  |  |
| 2017 | -0.128 | -0.141 | -0.139 | -0.139 | -0.139 | -0.139 | -0.138 |
|  | (0.144) | (0.144) | (0.144) | (0.144) | (0.144) | (0.144) | (0.144) |
|  |  |  |  |  |  |  |  |
| 2018 | 0.142 | 0.131 | 0.133 | 0.132 | 0.133 | 0.133 | 0.134 |
|  | (0.144) | (0.143) | (0.144) | (0.144) | (0.144) | (0.144) | (0.144) |
|  |  |  |  |  |  |  |  |
| 2019 | 0.411^**^ | 0.398^**^ | 0.401^**^ | 0.400^**^ | 0.400^**^ | 0.399^**^ | 0.399^**^ |
|  | (0.138) | (0.137) | (0.137) | (0.138) | (0.138) | (0.138) | (0.138) |
|  |  |  |  |  |  |  |  |
| MDMA | 0.0581 | -0.238 | 0.0485 | 0.0487 | 0.0488 | 0.0491 | 0.0464 |
|  | (0.0976) | (0.214) | (0.101) | (0.100) | (0.100) | (0.100) | (0.100) |
|  |  |  |  |  |  |  |  |
| LCPU | 0.160 |  |  |  |  |  |  |
|  | (0.183) |  |  |  |  |  |  |
|  |  |  |  |  |  |  |  |
| Educational Level | -0.440^***^ | -0.430^***^ | -0.431^***^ | -0.431^***^ | -0.431^***^ | -0.431^***^ | -0.433^***^ |
|  | (0.0280) | (0.0284) | (0.0285) | (0.0284) | (0.0284) | (0.0284) | (0.0285) |
|  |  |  |  |  |  |  |  |
| Family Income | -0.360^***^ | -0.383^***^ | -0.374^***^ | -0.376^***^ | -0.375^***^ | -0.373^***^ | -0.364^***^ |
|  | (0.0170) | (0.0175) | (0.0171) | (0.0170) | (0.0170) | (0.0165) | (0.0172) |
|  |  |  |  |  |  |  |  |
| LCPU * Family Income | -0.0595^†^ |  |  |  |  |  |  |
|  | (0.0324) |  |  |  |  |  |  |
|  |  |  |  |  |  |  |  |
| Psilocybin |  | -0.349^***^ | -0.297 | -0.346^***^ | -0.346^***^ | -0.347^***^ | -0.344^***^ |
|  |  | (0.0969) | (0.208) | (0.0970) | (0.0970) | (0.0970) | (0.0970) |
|  |  |  |  |  |  |  |  |
| DMT |  | 0.492 | 0.491 | -1.129 | 0.496 | 0.502 | 0.495 |
|  |  | (0.625) | (0.625) | (1.345) | (0.628) | (0.629) | (0.625) |
|  |  |  |  |  |  |  |  |
| Ayahuasca |  | 0.224 | 0.244 | 0.322 | -0.832 | 0.250 | 0.247 |
|  |  | (1.938) | (1.957) | (1.967) | (6.222) | (1.960) | (1.965) |
|  |  |  |  |  |  |  |  |
| Peyote/Mescaline |  | -0.536^***^ | -0.536^***^ | -0.539^***^ | -0.535^***^ | -0.363 | -0.538^***^ |
|  |  | (0.148) | (0.147) | (0.147) | (0.148) | (0.344) | (0.148) |
|  |  |  |  |  |  |  |  |
| LSD |  | 0.271^*^ | 0.269^*^ | 0.270^*^ | 0.270^*^ | 0.270^*^ | 0.598^**^ |
|  |  | (0.104) | (0.104) | (0.104) | (0.104) | (0.104) | (0.220) |
|  |  |  |  |  |  |  |  |
| MDMA * Family Income |  | 0.0576 |  |  |  |  |  |
|  |  | (0.0366) |  |  |  |  |  |
|  |  |  |  |  |  |  |  |
| Psilocybin * Family Income |  |  | -0.00951 |  |  |  |  |
|  |  |  | (0.0383) |  |  |  |  |
|  |  |  |  |  |  |  |  |
| DMT * Family Income |  |  |  | 0.336 |  |  |  |
|  |  |  |  | (0.241) |  |  |  |
|  |  |  |  |  |  |  |  |
| Ayahuasca * Family Income |  |  |  |  | 0.202 |  |  |
|  |  |  |  |  | (1.065) |  |  |
|  |  |  |  |  |  |  |  |
| Peyote/Mescaline * Family Income |  |  |  |  |  | -0.0347 |  |
|  |  |  |  |  |  | (0.0560) |  |
|  |  |  |  |  |  |  |  |
| LSD * Family Income |  |  |  |  |  |  | -0.0646^†^ |
|  |  |  |  |  |  |  | (0.0376) |
|  |  |  |  |  |  |  |  |
| Constant | 19.31^***^ | 19.37^***^ | 19.32^***^ | 19.34^***^ | 19.33^***^ | 19.32^***^ | 19.28^***^ |
|  | (0.261) | (0.261) | (0.261) | (0.261) | (0.260) | (0.261) | (0.262) |
| Observations | 97184 | 97184 | 97184 | 97184 | 97184 | 97184 | 97184 |
| *R*^2^ | 0.160 | 0.160 | 0.160 | 0.160 | 0.160 | 0.160 | 0.160 |
| Source: 2008-2019 National Survey of Drug Use and Health, N=458,372  Standard errors in parentheses  ^a^ Men serve as the references category  ^b^ Single Serves as the reference category for marital statuses  ^c^ 2008 serves as the reference category for the year survey was taken  ^†^ *p* < 0.1, ^*^ *p* < 0.05, ^**^ *p* < 0.01, ^***^ *p* < 0.001 | | | | | | | |

| Supplemental Table 3a. Weighted Multivariate Ordinary Squared Logistics Regression Predicting Psychological Distress Levels In the Past 30 Days Among Black People | | | | |
| --- | --- | --- | --- | --- |
|  | Model 1 | Model 2 | Model 3 | Model 4 |
| Age | -0.530^***^ | -0.509^***^ | -0.527^***^ | -0.505^***^ |
|  | (0.0322) | (0.0302) | (0.0321) | (0.0301) |
|  |  |  |  |  |
| ^a^Women | 0.745^***^ | 0.711^***^ | 0.761^***^ | 0.728^***^ |
|  | (0.149) | (0.149) | (0.148) | (0.148) |
|  |  |  |  |  |
| ^b^Married | -0.841^***^ | -0.167 | -0.847^***^ | -0.170 |
|  | (0.173) | (0.179) | (0.172) | (0.178) |
|  |  |  |  |  |
| Divorced | -0.0706 | -0.320 | -0.0831 | -0.337 |
|  | (0.392) | (0.384) | (0.392) | (0.383) |
|  |  |  |  |  |
| Widowed | 0.0841 | 0.0886 | 0.0888 | 0.0940 |
|  | (0.220) | (0.216) | (0.219) | (0.215) |
|  |  |  |  |  |
| Religious Salience | -0.0662^*^ | -0.0899^**^ | -0.0621^†^ | -0.0859^**^ |
|  | (0.0326) | (0.0325) | (0.0325) | (0.0324) |
|  |  |  |  |  |
| Religious Attendance | -0.293^***^ | -0.196^***^ | -0.289^***^ | -0.191^***^ |
|  | (0.0418) | (0.0413) | (0.0416) | (0.0413) |
|  |  |  |  |  |
| Cocaine | 0.258 | 0.00471 | 0.182 | -0.0665 |
|  | (0.243) | (0.243) | (0.242) | (0.240) |
|  |  |  |  |  |
| Stimulants | 0.542^†^ | 0.717^*^ | 0.492 | 0.670^*^ |
|  | (0.304) | (0.297) | (0.305) | (0.297) |
|  |  |  |  |  |
| Sedatives | 0.950^***^ | 0.936^***^ | 0.996^***^ | 0.983^***^ |
|  | (0.224) | (0.214) | (0.223) | (0.214) |
|  |  |  |  |  |
| Tranquilizers | 1.070^***^ | 1.010^***^ | 1.037^***^ | 0.977^***^ |
|  | (0.206) | (0.201) | (0.205) | (0.200) |
|  |  |  |  |  |
| Heroine | 0.609 | 0.231 | 0.723 | 0.346 |
|  | (0.610) | (0.625) | (0.610) | (0.624) |
|  |  |  |  |  |
| Pain Killers | 0.498^*^ | 0.483^**^ | 0.495^*^ | 0.483^**^ |
|  | (0.196) | (0.183) | (0.196) | (0.183) |
|  |  |  |  |  |
| Marijuana | -0.129 | -0.0349 | -0.129 | -0.0318 |
|  | (0.175) | (0.166) | (0.176) | (0.167) |
|  |  |  |  |  |
| PCP | 0.220 | 0.156 | 0.288 | 0.237 |
|  | (0.633) | (0.627) | (0.614) | (0.607) |
|  |  |  |  |  |
| Inhalants | 0.479 | 0.538^†^ | 0.434 | 0.491 |
|  | (0.325) | (0.314) | (0.319) | (0.310) |
|  |  |  |  |  |
| Tobacco | 1.108^***^ | 0.825^***^ | 1.106^***^ | 0.821^***^ |
|  | (0.153) | (0.151) | (0.154) | (0.151) |
|  |  |  |  |  |
| Age of 1^st^ Alcohol Use | 0.0540 | -0.0218 | 0.0543 | -0.0224 |
|  | (0.0704) | (0.0664) | (0.0704) | (0.0663) |
|  |  |  |  |  |
| Risk Behaviors | 0.628^***^ | 0.757^***^ | 0.616^***^ | 0.747^***^ |
|  | (0.0959) | (0.0966) | (0.0953) | (0.0961) |
|  |  |  |  |  |
| ^c^2009 | -0.0608 | -0.0229 | -0.0732 | -0.0344 |
|  | (0.271) | (0.275) | (0.276) | (0.281) |
|  |  |  |  |  |
| 2010 | 0.447 | 0.331 | 0.449 | 0.331 |
|  | (0.357) | (0.332) | (0.355) | (0.330) |
|  |  |  |  |  |
| 2011 | 0.390 | 0.344 | 0.391 | 0.344 |
|  | (0.371) | (0.382) | (0.369) | (0.380) |
|  |  |  |  |  |
| 2012 | 1.138^***^ | 1.086^**^ | 1.133^***^ | 1.080^**^ |
|  | (0.327) | (0.329) | (0.325) | (0.327) |
|  |  |  |  |  |
| 2013 | 0.438 | 0.412 | 0.424 | 0.398 |
|  | (0.363) | (0.341) | (0.363) | (0.341) |
|  |  |  |  |  |
| 2014 | 0.498 | 0.535^†^ | 0.483 | 0.522^†^ |
|  | (0.313) | (0.302) | (0.314) | (0.303) |
|  |  |  |  |  |
| 2015 | -0.267 | -0.183 | -0.264 | -0.181 |
|  | (0.308) | (0.306) | (0.307) | (0.306) |
|  |  |  |  |  |
| 2016 | -0.179 | -0.0696 | -0.194 | -0.0855 |
|  | (0.284) | (0.285) | (0.282) | (0.284) |
|  |  |  |  |  |
| 2017 | 0.394 | 0.475 | 0.379 | 0.458 |
|  | (0.297) | (0.301) | (0.297) | (0.302) |
|  |  |  |  |  |
| 2018 | 0.609^†^ | 0.740^*^ | 0.588^†^ | 0.719^*^ |
|  | (0.350) | (0.347) | (0.352) | (0.349) |
|  |  |  |  |  |
| 2019 | 0.896^**^ | 1.145^***^ | 0.858^**^ | 1.108^***^ |
|  | (0.322) | (0.318) | (0.323) | (0.319) |
|  |  |  |  |  |
| MDMA | 0.554^†^ | 0.580^†^ | 0.325 | 0.344 |
|  | (0.302) | (0.294) | (0.304) | (0.298) |
|  |  |  |  |  |
| LCPU | -0.693^*^ | -0.493 |  |  |
|  | (0.339) | (0.343) |  |  |
|  |  |  |  |  |
| Educational Level |  | -0.348^***^ |  | -0.356^***^ |
|  |  | (0.0681) |  | (0.0687) |
|  |  |  |  |  |
| Family Income |  | -0.411^***^ |  | -0.413^***^ |
|  |  | (0.0305) |  | (0.0305) |
|  |  |  |  |  |
| Psilocybin |  |  | 1.391 | 1.559^***^ |
|  |  |  | (0.457) | (0.459) |
|  |  |  |  |  |
| DMT |  |  | -1.677 | -2.305 |
|  |  |  | (6.761) | (6.563) |
|  |  |  |  |  |
| Ayahuasca |  |  | 0 | 0 |
|  |  |  | (.) | (.) |
|  |  |  |  |  |
| Peyote/Mescaline |  |  | -1.137^*^ | -1.090^*^ |
|  |  |  | (0.556) | (0.542) |
|  |  |  |  |  |
| LSD |  |  | -0.474 | -0.363 |
|  |  |  | (0.429) | (0.422) |
|  |  |  |  |  |
| Constant | 15.13^***^ | 17.26^***^ | 15.07^***^ | 17.22^***^ |
|  | (0.594) | (0.624) | (0.591) | (0.620) |
| Observations | 16823 | 16823 | 16823 | 16823 |
| *R*^2^ | 0.104 | 0.129 | 0.105 | 0.131 |
| Source: 2008-2019 National Survey of Drug Use and Health, N=458,372  Standard errors in parentheses  ^a^ Men serve as the references category  ^b^ Single Serves as the reference category for marital statuses  ^c^ 2008 serves as the reference category for the year survey was taken  ^†^ *p* < 0.1, ^*^ *p* < 0.05, ^**^ *p* < 0.01, ^***^ *p* < 0.001 | | | | |

| Supplemental Table 3b. Weighted Multivariate Ordinary Squared Logistics Regression Predicting Psychological Distress Levels In the Past 30 Days Among Black People with interaction terms (Psychedelics * Educational Level) | | | | | | | |
| --- | --- | --- | --- | --- | --- | --- | --- |
|  | Model 5 | Model 6 | Model 7 | Model 8 | Model 9 | Model 10 | Model 11 |
| Age | -0.509^***^ | -0.507^***^ | -0.505^***^ | -0.506^***^ | -0.505^***^ | -0.506^***^ | -0.505^***^ |
|  | (0.0302) | (0.0299) | (0.0301) | (0.0301) | (0.0301) | (0.0301) | (0.0300) |
|  |  |  |  |  |  |  |  |
| ^a^Women | 0.711^***^ | 0.727^***^ | 0.727^***^ | 0.729^***^ | 0.728^***^ | 0.729^***^ | 0.729^***^ |
|  | (0.149) | (0.148) | (0.148) | (0.148) | (0.148) | (0.148) | (0.148) |
|  |  |  |  |  |  |  |  |
| ^b^Married | -0.167 | -0.166 | -0.172 | -0.169 | -0.170 | -0.170 | -0.169 |
|  | (0.179) | (0.178) | (0.178) | (0.178) | (0.178) | (0.178) | (0.178) |
|  |  |  |  |  |  |  |  |
| Divorced | -0.320 | -0.342 | -0.341 | -0.336 | -0.337 | -0.338 | -0.337 |
|  | (0.384) | (0.383) | (0.382) | (0.383) | (0.383) | (0.383) | (0.383) |
|  |  |  |  |  |  |  |  |
| Widowed | 0.0882 | 0.0911 | 0.0922 | 0.0946 | 0.0940 | 0.0950 | 0.0930 |
|  | (0.215) | (0.215) | (0.215) | (0.215) | (0.215) | (0.215) | (0.215) |
|  |  |  |  |  |  |  |  |
| Religious Salience | -0.0898^**^ | -0.0854^**^ | -0.0864^**^ | -0.0859^**^ | -0.0859^**^ | -0.0860^**^ | -0.0858^**^ |
|  | (0.0324) | (0.0324) | (0.0324) | (0.0324) | (0.0324) | (0.0324) | (0.0324) |
|  |  |  |  |  |  |  |  |
| Religious Attendance | -0.196^***^ | -0.191^***^ | -0.191^***^ | -0.191^***^ | -0.191^***^ | -0.191^***^ | -0.191^***^ |
|  | (0.0414) | (0.0413) | (0.0413) | (0.0413) | (0.0413) | (0.0413) | (0.0413) |
|  |  |  |  |  |  |  |  |
| Cocaine | 0.00305 | -0.0648 | -0.0649 | -0.0675 | -0.0665 | -0.0657 | -0.0705 |
|  | (0.243) | (0.240) | (0.239) | (0.240) | (0.240) | (0.239) | (0.241) |
|  |  |  |  |  |  |  |  |
| Stimulants | 0.713^*^ | 0.664^*^ | 0.684^*^ | 0.672^*^ | 0.670^*^ | 0.674^*^ | 0.664^*^ |
|  | (0.297) | (0.297) | (0.297) | (0.297) | (0.297) | (0.297) | (0.297) |
|  |  |  |  |  |  |  |  |
| Sedatives | 0.936^***^ | 0.977^***^ | 0.979^***^ | 0.983^***^ | 0.983^***^ | 0.985^***^ | 0.982^***^ |
|  | (0.214) | (0.215) | (0.215) | (0.214) | (0.214) | (0.214) | (0.214) |
|  |  |  |  |  |  |  |  |
| Tranquilizers | 1.010^***^ | 0.979^***^ | 0.976^***^ | 0.977^***^ | 0.977^***^ | 0.979^***^ | 0.977^***^ |
|  | (0.201) | (0.200) | (0.200) | (0.200) | (0.200) | (0.201) | (0.200) |
|  |  |  |  |  |  |  |  |
| Heroine | 0.248 | 0.347 | 0.323 | 0.353 | 0.346 | 0.331 | 0.381 |
|  | (0.629) | (0.624) | (0.624) | (0.623) | (0.624) | (0.629) | (0.619) |
|  |  |  |  |  |  |  |  |
| Pain Killers | 0.485^**^ | 0.489^**^ | 0.479^*^ | 0.482^**^ | 0.483^**^ | 0.481^**^ | 0.486^**^ |
|  | (0.183) | (0.182) | (0.184) | (0.183) | (0.183) | (0.184) | (0.182) |
|  |  |  |  |  |  |  |  |
| Marijuana | -0.0349 | -0.0334 | -0.0305 | -0.0319 | -0.0318 | -0.0321 | -0.0315 |
|  | (0.166) | (0.167) | (0.167) | (0.167) | (0.167) | (0.167) | (0.167) |
|  |  |  |  |  |  |  |  |
| PCP | 0.156 | 0.251 | 0.228 | 0.242 | 0.237 | 0.241 | 0.236 |
|  | (0.627) | (0.607) | (0.608) | (0.608) | (0.607) | (0.609) | (0.607) |
|  |  |  |  |  |  |  |  |
| Inhalants | 0.539^†^ | 0.488 | 0.471 | 0.493 | 0.491 | 0.489 | 0.490 |
|  | (0.314) | (0.309) | (0.310) | (0.309) | (0.310) | (0.311) | (0.309) |
|  |  |  |  |  |  |  |  |
| Tobacco | 0.824^***^ | 0.821^***^ | 0.824^***^ | 0.821^***^ | 0.821^***^ | 0.822^***^ | 0.820^***^ |
|  | (0.151) | (0.151) | (0.151) | (0.151) | (0.151) | (0.151) | (0.152) |
|  |  |  |  |  |  |  |  |
| Age of 1^st^ Alcohol Use | -0.0220 | -0.0236 | -0.0218 | -0.0223 | -0.0224 | -0.0223 | -0.0230 |
|  | (0.0664) | (0.0662) | (0.0663) | (0.0663) | (0.0663) | (0.0663) | (0.0664) |
|  |  |  |  |  |  |  |  |
| Risk Behaviors | 0.758^***^ | 0.747^***^ | 0.746^***^ | 0.747^***^ | 0.747^***^ | 0.747^***^ | 0.747^***^ |
|  | (0.0968) | (0.0963) | (0.0962) | (0.0961) | (0.0961) | (0.0961) | (0.0963) |
|  |  |  |  |  |  |  |  |
| ^c^2009 | -0.0219 | -0.0319 | -0.0327 | -0.0344 | -0.0344 | -0.0353 | -0.0334 |
|  | (0.275) | (0.281) | (0.281) | (0.281) | (0.281) | (0.281) | (0.281) |
|  |  |  |  |  |  |  |  |
| 2010 | 0.332 | 0.334 | 0.336 | 0.331 | 0.331 | 0.329 | 0.332 |
|  | (0.332) | (0.330) | (0.330) | (0.330) | (0.330) | (0.331) | (0.330) |
|  |  |  |  |  |  |  |  |
| 2011 | 0.343 | 0.346 | 0.345 | 0.344 | 0.344 | 0.344 | 0.341 |
|  | (0.381) | (0.380) | (0.380) | (0.380) | (0.380) | (0.380) | (0.379) |
|  |  |  |  |  |  |  |  |
| 2012 | 1.087^**^ | 1.086^**^ | 1.082^**^ | 1.082^**^ | 1.080^**^ | 1.079^**^ | 1.081^**^ |
|  | (0.329) | (0.327) | (0.327) | (0.328) | (0.327) | (0.328) | (0.327) |
|  |  |  |  |  |  |  |  |
| 2013 | 0.413 | 0.399 | 0.400 | 0.396 | 0.398 | 0.397 | 0.399 |
|  | (0.341) | (0.341) | (0.341) | (0.341) | (0.341) | (0.342) | (0.341) |
|  |  |  |  |  |  |  |  |
| 2014 | 0.536^†^ | 0.522^†^ | 0.525^†^ | 0.522^†^ | 0.522^†^ | 0.521^†^ | 0.522^†^ |
|  | (0.302) | (0.303) | (0.303) | (0.303) | (0.303) | (0.304) | (0.303) |
|  |  |  |  |  |  |  |  |
| 2015 | -0.185 | -0.186 | -0.174 | -0.181 | -0.181 | -0.182 | -0.184 |
|  | (0.306) | (0.305) | (0.305) | (0.306) | (0.306) | (0.305) | (0.305) |
|  |  |  |  |  |  |  |  |
| 2016 | -0.0691 | -0.0860 | -0.0779 | -0.0855 | -0.0855 | -0.0877 | -0.0857 |
|  | (0.285) | (0.283) | (0.285) | (0.284) | (0.284) | (0.285) | (0.284) |
|  |  |  |  |  |  |  |  |
| 2017 | 0.474 | 0.449 | 0.471 | 0.459 | 0.458 | 0.458 | 0.456 |
|  | (0.301) | (0.301) | (0.301) | (0.302) | (0.302) | (0.301) | (0.301) |
|  |  |  |  |  |  |  |  |
| 2018 | 0.740^*^ | 0.716^*^ | 0.723^*^ | 0.719^*^ | 0.719^*^ | 0.719^*^ | 0.718^*^ |
|  | (0.347) | (0.348) | (0.348) | (0.349) | (0.349) | (0.349) | (0.348) |
|  |  |  |  |  |  |  |  |
| 2019 | 1.145^***^ | 1.103^***^ | 1.118^***^ | 1.108^***^ | 1.108^***^ | 1.107^***^ | 1.106^***^ |
|  | (0.318) | (0.319) | (0.321) | (0.319) | (0.319) | (0.319) | (0.319) |
|  |  |  |  |  |  |  |  |
| MDMA | 0.581^†^ | -0.399 | 0.344 | 0.344 | 0.344 | 0.344 | 0.346 |
|  | (0.294) | (0.658) | (0.298) | (0.298) | (0.298) | (0.298) | (0.299) |
|  |  |  |  |  |  |  |  |
| LCPU | -0.665 |  |  |  |  |  |  |
|  | (0.784) |  |  |  |  |  |  |
|  |  |  |  |  |  |  |  |
| Educational Level | -0.353^***^ | -0.374^***^ | -0.344^***^ | -0.356^***^ | -0.356^***^ | -0.354^***^ | -0.363^***^ |
|  | (0.0710) | (0.0739) | (0.0685) | (0.0687) | (0.0687) | (0.0682) | (0.0699) |
|  |  |  |  |  |  |  |  |
| Family Income | -0.411^***^ | -0.413^***^ | -0.413^***^ | -0.413^***^ | -0.413^***^ | -0.413^***^ | -0.412^***^ |
|  | (0.0306) | (0.0305) | (0.0305) | (0.0305) | (0.0305) | (0.0305) | (0.0306) |
|  |  |  |  |  |  |  |  |
| LCPU * Educational Level | 0.0631 |  |  |  |  |  |  |
|  | (0.266) |  |  |  |  |  |  |
|  |  |  |  |  |  |  |  |
| Psilocybin |  | 1.525^**^ | 2.985^*^ | 1.559^***^ | 1.559^***^ | 1.556^***^ | 1.556^***^ |
|  |  | (0.458) | (1.166) | (0.459) | (0.459) | (0.457) | (0.458) |
|  |  |  |  |  |  |  |  |
| DMT |  | -2.271 | -2.438 | 43.84^***^ | -2.305 | -2.332 | -2.281 |
|  |  | (6.670) | (6.368) | (2.538) | (6.563) | (6.527) | (6.622) |
|  |  |  |  |  |  |  |  |
| Ayahuasca |  | 0 | 0 | 0 | 0 | 0 | 0 |
|  |  | (.) | (.) | (.) | (.) | (.) | (.) |
|  |  |  |  |  |  |  |  |
| Peyote/Mescaline |  | -1.093^*^ | -1.092^*^ | -1.092^*^ | -1.090^*^ | -0.750 | -1.107^*^ |
|  |  | (0.540) | (0.543) | (0.542) | (0.542) | (1.683) | (0.546) |
|  |  |  |  |  |  |  |  |
| LSD |  | -0.362 | -0.368 | -0.366 | -0.363 | -0.361 | -0.736 |
|  |  | (0.422) | (0.421) | (0.422) | (0.422) | (0.422) | (0.787) |
|  |  |  |  |  |  |  |  |
| MDMA * Educational Level |  | 0.291 |  |  |  |  |  |
|  |  | (0.232) |  |  |  |  |  |
|  |  |  |  |  |  |  |  |
| Psilocybin * Educational Level |  |  | -0.509 |  |  |  |  |
|  |  |  | (0.402) |  |  |  |  |
|  |  |  |  |  |  |  |  |
| DMT * Educational Level |  |  |  | -18.60^***^ |  |  |  |
|  |  |  |  | (1.009) |  |  |  |
|  |  |  |  |  |  |  |  |
| Ayahuasca * Educational Level |  |  |  |  | 0 |  |  |
|  |  |  |  |  | (.) |  |  |
|  |  |  |  |  |  |  |  |
| Peyote/Mescaline * Educational Level |  |  |  |  |  | -0.124 |  |
|  |  |  |  |  |  | (0.550) |  |
|  |  |  |  |  |  |  |  |
| LSD * Educational Level |  |  |  |  |  |  | 0.142 |
|  |  |  |  |  |  |  | (0.278) |
|  |  |  |  |  |  |  |  |
| Constant | 17.27^***^ | 17.29^***^ | 17.18^***^ | 17.22^***^ | 17.22^***^ | 17.22^***^ | 17.24^***^ |
|  | (0.627) | (0.624) | (0.619) | (0.620) | (0.620) | (0.620) | (0.621) |
| Observations | 16823 | 16823 | 16823 | 16823 | 16823 | 16823 | 16823 |
| *R*^2^ | 0.129 | 0.131 | 0.131 | 0.131 | 0.131 | 0.131 | 0.131 |
| Source: 2008-2019 National Survey of Drug Use and Health, N=458,372  Standard errors in parentheses  ^a^ Men serve as the references category  ^b^ Single Serves as the reference category for marital statuses  ^c^ 2008 serves as the reference category for the year survey was taken  ^†^ *p* < 0.1, ^*^ *p* < 0.05, ^**^ *p* < 0.01, ^***^ *p* < 0.001 | | | | | | | |

| Supplemental Table 3c. Weighted Multivariate Ordinary Squared Logistics Regression Predicting Psychological Distress Levels In the Past 30 Days Among Black People with interaction terms (Psychedelics * Family Income Level) | | | | | | | |
| --- | --- | --- | --- | --- | --- | --- | --- |
|  | Model 12 | Model 13 | Model 14 | Model 15 | Model 16 | Model 17 | Model 18 |
| Age | -0.509^***^ | -0.506^***^ | -0.505^***^ | -0.506^***^ | -0.505^***^ | -0.505^***^ | -0.506^***^ |
|  | (0.0302) | (0.0300) | (0.0300) | (0.0301) | (0.0301) | (0.0301) | (0.0301) |
|  |  |  |  |  |  |  |  |
| ^a^Women | 0.711^***^ | 0.731^***^ | 0.731^***^ | 0.729^***^ | 0.728^***^ | 0.729^***^ | 0.728^***^ |
|  | (0.149) | (0.147) | (0.148) | (0.148) | (0.148) | (0.148) | (0.148) |
|  |  |  |  |  |  |  |  |
| ^b^Married | -0.167 | -0.158 | -0.168 | -0.169 | -0.170 | -0.170 | -0.171 |
|  | (0.179) | (0.179) | (0.178) | (0.178) | (0.178) | (0.178) | (0.178) |
|  |  |  |  |  |  |  |  |
| Divorced | -0.320 | -0.341 | -0.338 | -0.336 | -0.337 | -0.337 | -0.336 |
|  | (0.384) | (0.383) | (0.383) | (0.383) | (0.383) | (0.382) | (0.383) |
|  |  |  |  |  |  |  |  |
| Widowed | 0.0884 | 0.0937 | 0.0953 | 0.0946 | 0.0940 | 0.0923 | 0.0935 |
|  | (0.216) | (0.215) | (0.215) | (0.215) | (0.215) | (0.215) | (0.215) |
|  |  |  |  |  |  |  |  |
| Religious Salience | -0.0899^**^ | -0.0854^**^ | -0.0862^**^ | -0.0859^**^ | -0.0859^**^ | -0.0859^**^ | -0.0856^**^ |
|  | (0.0325) | (0.0324) | (0.0325) | (0.0324) | (0.0324) | (0.0324) | (0.0324) |
|  |  |  |  |  |  |  |  |
| Religious Attendance | -0.196^***^ | -0.191^***^ | -0.191^***^ | -0.191^***^ | -0.191^***^ | -0.191^***^ | -0.191^***^ |
|  | (0.0414) | (0.0413) | (0.0413) | (0.0413) | (0.0413) | (0.0412) | (0.0413) |
|  |  |  |  |  |  |  |  |
| Cocaine | 0.00483 | -0.0696 | -0.0671 | -0.0675 | -0.0665 | -0.0653 | -0.0631 |
|  | (0.243) | (0.240) | (0.240) | (0.240) | (0.240) | (0.240) | (0.241) |
|  |  |  |  |  |  |  |  |
| Stimulants | 0.717^*^ | 0.662^*^ | 0.665^*^ | 0.672^*^ | 0.670^*^ | 0.674^*^ | 0.669^*^ |
|  | (0.296) | (0.296) | (0.296) | (0.297) | (0.297) | (0.297) | (0.297) |
|  |  |  |  |  |  |  |  |
| Sedatives | 0.936^***^ | 0.975^***^ | 0.980^***^ | 0.983^***^ | 0.983^***^ | 0.986^***^ | 0.984^***^ |
|  | (0.214) | (0.214) | (0.213) | (0.214) | (0.214) | (0.215) | (0.215) |
|  |  |  |  |  |  |  |  |
| Tranquilizers | 1.010^***^ | 0.984^***^ | 0.976^***^ | 0.977^***^ | 0.977^***^ | 0.976^***^ | 0.975^***^ |
|  | (0.201) | (0.199) | (0.199) | (0.200) | (0.200) | (0.200) | (0.199) |
|  |  |  |  |  |  |  |  |
| Heroine | 0.229 | 0.369 | 0.351 | 0.353 | 0.346 | 0.327 | 0.327 |
|  | (0.620) | (0.628) | (0.624) | (0.623) | (0.624) | (0.628) | (0.618) |
|  |  |  |  |  |  |  |  |
| Pain Killers | 0.483^**^ | 0.483^**^ | 0.484^**^ | 0.482^**^ | 0.483^**^ | 0.480^*^ | 0.482^**^ |
|  | (0.184) | (0.183) | (0.183) | (0.183) | (0.183) | (0.184) | (0.183) |
|  |  |  |  |  |  |  |  |
| Marijuana | -0.0348 | -0.0306 | -0.0329 | -0.0319 | -0.0318 | -0.0311 | -0.0318 |
|  | (0.166) | (0.167) | (0.167) | (0.167) | (0.167) | (0.167) | (0.167) |
|  |  |  |  |  |  |  |  |
| PCP | 0.156 | 0.251 | 0.246 | 0.242 | 0.237 | 0.218 | 0.238 |
|  | (0.627) | (0.606) | (0.606) | (0.608) | (0.607) | (0.600) | (0.607) |
|  |  |  |  |  |  |  |  |
| Inhalants | 0.538^†^ | 0.484 | 0.494 | 0.493 | 0.491 | 0.483 | 0.490 |
|  | (0.314) | (0.309) | (0.309) | (0.309) | (0.310) | (0.310) | (0.309) |
|  |  |  |  |  |  |  |  |
| Tobacco | 0.825^***^ | 0.820^***^ | 0.819^***^ | 0.821^***^ | 0.821^***^ | 0.822^***^ | 0.824^***^ |
|  | (0.150) | (0.151) | (0.151) | (0.151) | (0.151) | (0.151) | (0.150) |
|  |  |  |  |  |  |  |  |
| Age of 1^st^ Alcohol Use | -0.0218 | -0.0227 | -0.0229 | -0.0223 | -0.0224 | -0.0225 | -0.0221 |
|  | (0.0663) | (0.0663) | (0.0662) | (0.0663) | (0.0663) | (0.0663) | (0.0662) |
|  |  |  |  |  |  |  |  |
| Risk Behaviors | 0.757^***^ | 0.747^***^ | 0.748^***^ | 0.747^***^ | 0.747^***^ | 0.746^***^ | 0.745^***^ |
|  | (0.0968) | (0.0962) | (0.0961) | (0.0961) | (0.0961) | (0.0961) | (0.0963) |
|  |  |  |  |  |  |  |  |
| ^c^2009 | -0.0230 | -0.0349 | -0.0333 | -0.0344 | -0.0344 | -0.0373 | -0.0354 |
|  | (0.275) | (0.282) | (0.282) | (0.281) | (0.281) | (0.282) | (0.281) |
|  |  |  |  |  |  |  |  |
| 2010 | 0.331 | 0.327 | 0.331 | 0.331 | 0.331 | 0.329 | 0.329 |
|  | (0.333) | (0.331) | (0.330) | (0.330) | (0.330) | (0.331) | (0.330) |
|  |  |  |  |  |  |  |  |
| 2011 | 0.344 | 0.342 | 0.345 | 0.344 | 0.344 | 0.342 | 0.346 |
|  | (0.382) | (0.381) | (0.380) | (0.380) | (0.380) | (0.380) | (0.380) |
|  |  |  |  |  |  |  |  |
| 2012 | 1.086^**^ | 1.075^**^ | 1.077^**^ | 1.082^**^ | 1.080^**^ | 1.079^**^ | 1.078^**^ |
|  | (0.330) | (0.327) | (0.327) | (0.328) | (0.327) | (0.328) | (0.328) |
|  |  |  |  |  |  |  |  |
| 2013 | 0.412 | 0.392 | 0.398 | 0.396 | 0.398 | 0.396 | 0.395 |
|  | (0.341) | (0.340) | (0.341) | (0.341) | (0.341) | (0.342) | (0.341) |
|  |  |  |  |  |  |  |  |
| 2014 | 0.535^†^ | 0.517^†^ | 0.522^†^ | 0.522^†^ | 0.522^†^ | 0.520^†^ | 0.520^†^ |
|  | (0.302) | (0.303) | (0.303) | (0.303) | (0.303) | (0.304) | (0.303) |
|  |  |  |  |  |  |  |  |
| 2015 | -0.183 | -0.189 | -0.182 | -0.181 | -0.181 | -0.181 | -0.183 |
|  | (0.306) | (0.306) | (0.306) | (0.306) | (0.306) | (0.306) | (0.306) |
|  |  |  |  |  |  |  |  |
| 2016 | -0.0698 | -0.0850 | -0.0859 | -0.0855 | -0.0855 | -0.0896 | -0.0868 |
|  | (0.286) | (0.284) | (0.284) | (0.284) | (0.284) | (0.285) | (0.285) |
|  |  |  |  |  |  |  |  |
| 2017 | 0.475 | 0.450 | 0.457 | 0.459 | 0.458 | 0.458 | 0.459 |
|  | (0.301) | (0.302) | (0.302) | (0.302) | (0.302) | (0.302) | (0.302) |
|  |  |  |  |  |  |  |  |
| 2018 | 0.740^*^ | 0.717^*^ | 0.721^*^ | 0.719^*^ | 0.719^*^ | 0.721^*^ | 0.715^*^ |
|  | (0.347) | (0.349) | (0.349) | (0.349) | (0.349) | (0.350) | (0.350) |
|  |  |  |  |  |  |  |  |
| 2019 | 1.145^***^ | 1.102^***^ | 1.110^***^ | 1.108^***^ | 1.108^***^ | 1.107^***^ | 1.108^***^ |
|  | (0.317) | (0.319) | (0.319) | (0.319) | (0.319) | (0.320) | (0.320) |
|  |  |  |  |  |  |  |  |
| MDMA | 0.579^†^ | -0.178 | 0.347 | 0.344 | 0.344 | 0.349 | 0.344 |
|  | (0.294) | (0.533) | (0.298) | (0.298) | (0.298) | (0.299) | (0.298) |
|  |  |  |  |  |  |  |  |
| LCPU | -0.463 |  |  |  |  |  |  |
|  | (0.576) |  |  |  |  |  |  |
|  |  |  |  |  |  |  |  |
| Educational Level | -0.349^***^ | -0.354^***^ | -0.356^***^ | -0.356^***^ | -0.356^***^ | -0.357^***^ | -0.357^***^ |
|  | (0.0680) | (0.0684) | (0.0687) | (0.0687) | (0.0687) | (0.0688) | (0.0686) |
|  |  |  |  |  |  |  |  |
| Family Income | -0.411^***^ | -0.423^***^ | -0.416^***^ | -0.413^***^ | -0.413^***^ | -0.410^***^ | -0.408^***^ |
|  | (0.0314) | (0.0323) | (0.0313) | (0.0305) | (0.0305) | (0.0310) | (0.0314) |
|  |  |  |  |  |  |  |  |
| LCPU * Family Income | -0.00734 |  |  |  |  |  |  |
|  | (0.130) |  |  |  |  |  |  |
|  |  |  |  |  |  |  |  |
| Psilocybin |  | 1.553^***^ | 0.982 | 1.559^***^ | 1.559^***^ | 1.555^**^ | 1.556^**^ |
|  |  | (0.457) | (0.810) | (0.459) | (0.459) | (0.461) | (0.461) |
|  |  |  |  |  |  |  |  |
| DMT |  | -2.122 | -2.100 | -49.18^***^ | -2.305 | -2.480 | -2.423 |
|  |  | (6.536) | (6.478) | (2.665) | (6.563) | (6.596) | (6.582) |
|  |  |  |  |  |  |  |  |
| Ayahuasca |  | 0 | 0 | 0 | 0 | 0 | 0 |
|  |  | (.) | (.) | (.) | (.) | (.) | (.) |
|  |  |  |  |  |  |  |  |
| Peyote/Mescaline |  | -1.104^*^ | -1.082^*^ | -1.092^*^ | -1.090^*^ | -0.572 | -1.099^*^ |
|  |  | (0.542) | (0.541) | (0.542) | (0.542) | (1.192) | (0.539) |
|  |  |  |  |  |  |  |  |
| LSD |  | -0.368 | -0.360 | -0.366 | -0.363 | -0.357 | 0.0254 |
|  |  | (0.421) | (0.422) | (0.422) | (0.422) | (0.420) | (0.675) |
|  |  |  |  |  |  |  |  |
| MDMA * Family Income |  | 0.135 |  |  |  |  |  |
|  |  | (0.111) |  |  |  |  |  |
|  |  |  |  |  |  |  |  |
| Psilocybin * Family Income |  |  | 0.138 |  |  |  |  |
|  |  |  | (0.168) |  |  |  |  |
|  |  |  |  |  |  |  |  |
| DMT * Family Income |  |  |  | 18.60^***^ |  |  |  |
|  |  |  |  | (1.009) |  |  |  |
|  |  |  |  |  |  |  |  |
| Ayahuasca * Family Income |  |  |  |  | 0 |  |  |
|  |  |  |  |  | (.) |  |  |
|  |  |  |  |  |  |  |  |
| Peyote/Mescaline * Family Income |  |  |  |  |  | -0.128 |  |
|  |  |  |  |  |  | (0.242) |  |
|  |  |  |  |  |  |  |  |
| LSD * Family Income |  |  |  |  |  |  | -0.0952 |
|  |  |  |  |  |  |  | (0.151) |
|  |  |  |  |  |  |  |  |
| Constant | 17.26^***^ | 17.27^***^ | 17.24^***^ | 17.22^***^ | 17.22^***^ | 17.22^***^ | 17.21^***^ |
|  | (0.624) | (0.624) | (0.619) | (0.620) | (0.620) | (0.620) | (0.619) |
| Observations | 16823 | 16823 | 16823 | 16823 | 16823 | 16823 | 16823 |
| *R*^2^ | 0.129 | 0.131 | 0.131 | 0.131 | 0.131 | 0.131 | 0.131 |
| Source: 2008-2019 National Survey of Drug Use and Health, N=458,372  Standard errors in parentheses  ^a^ Men serve as the references category  ^b^ Single Serves as the reference category for marital statuses  ^c^ 2008 serves as the reference category for the year survey was taken  ^†^ *p* < 0.1, ^*^ *p* < 0.05, ^**^ *p* < 0.01, ^***^ *p* < 0.001 | | | | | | | |

| Supplemental Table 4a. Weighted Multivariate Ordinary Squared Logistics Regression Predicting Psychological Distress Levels In the Past 30 Days Among Hispanics | | | | |
| --- | --- | --- | --- | --- |
|  | Model 1 | Model 2 | Model 3 | Model 4 |
| Age | -0.376^***^ | -0.368^***^ | -0.377^***^ | -0.369^***^ |
|  | (0.0341) | (0.0347) | (0.0341) | (0.0347) |
|  |  |  |  |  |
| ^a^Women | 1.056^***^ | 1.039^***^ | 1.047^***^ | 1.032^***^ |
|  | (0.143) | (0.144) | (0.143) | (0.144) |
|  |  |  |  |  |
| ^b^Married | -1.102^***^ | -0.715^***^ | -1.094^***^ | -0.709^***^ |
|  | (0.188) | (0.191) | (0.187) | (0.191) |
|  |  |  |  |  |
| Divorced | 0.414 | 0.0172 | 0.422 | 0.0274 |
|  | (0.539) | (0.538) | (0.541) | (0.540) |
|  |  |  |  |  |
| Widowed | 0.0359 | -0.0290 | 0.0337 | -0.0313 |
|  | (0.254) | (0.253) | (0.254) | (0.254) |
|  |  |  |  |  |
| Religious Salience | 0.0102 | -0.0346 | 0.00781 | -0.0364 |
|  | (0.0320) | (0.0310) | (0.0320) | (0.0310) |
|  |  |  |  |  |
| Religious Attendance | -0.178^***^ | -0.118^**^ | -0.180^***^ | -0.120^**^ |
|  | (0.0435) | (0.0424) | (0.0433) | (0.0422) |
|  |  |  |  |  |
| Cocaine | 0.0608 | -0.177 | 0.123 | -0.117 |
|  | (0.212) | (0.212) | (0.217) | (0.216) |
|  |  |  |  |  |
| Stimulants | 0.997^***^ | 1.018^***^ | 1.032^***^ | 1.049^***^ |
|  | (0.234) | (0.228) | (0.236) | (0.229) |
|  |  |  |  |  |
| Sedatives | 1.045^**^ | 1.081^**^ | 1.050^**^ | 1.082^**^ |
|  | (0.337) | (0.323) | (0.336) | (0.322) |
|  |  |  |  |  |
| Tranquilizers | 1.358^***^ | 1.372^***^ | 1.372^***^ | 1.382^***^ |
|  | (0.190) | (0.188) | (0.189) | (0.187) |
|  |  |  |  |  |
| Heroine | 0.584 | 0.131 | 0.592 | 0.128 |
|  | (0.495) | (0.478) | (0.495) | (0.479) |
|  |  |  |  |  |
| Pain Killers | 0.407^*^ | 0.463^**^ | 0.412^*^ | 0.466^**^ |
|  | (0.157) | (0.152) | (0.157) | (0.152) |
|  |  |  |  |  |
| Marijuana | -0.0788 | 0.122 | -0.0787 | 0.124 |
|  | (0.194) | (0.194) | (0.192) | (0.193) |
|  |  |  |  |  |
| PCP | 0.361 | 0.189 | 0.461 | 0.267 |
|  | (0.611) | (0.596) | (0.612) | (0.598) |
|  |  |  |  |  |
| Inhalants | 0.554^*^ | 0.593^*^ | 0.629^*^ | 0.653^*^ |
|  | (0.260) | (0.258) | (0.251) | (0.250) |
|  |  |  |  |  |
| Tobacco | 0.697^***^ | 0.630^***^ | 0.698^***^ | 0.632^***^ |
|  | (0.177) | (0.171) | (0.176) | (0.171) |
|  |  |  |  |  |
| Age of 1^st^ Alcohol Use | -0.0232 | -0.108 | -0.0227 | -0.108 |
|  | (0.0765) | (0.0758) | (0.0765) | (0.0758) |
|  |  |  |  |  |
| Risk Behaviors | 0.776^***^ | 0.909^***^ | 0.782^***^ | 0.913^***^ |
|  | (0.112) | (0.112) | (0.112) | (0.112) |
|  |  |  |  |  |
| ^c^2009 | 0.172 | 0.154 | 0.154 | 0.137 |
|  | (0.408) | (0.413) | (0.408) | (0.413) |
|  |  |  |  |  |
| 2010 | 0.0904 | -0.0162 | 0.0822 | -0.0247 |
|  | (0.361) | (0.366) | (0.359) | (0.364) |
|  |  |  |  |  |
| 2011 | -0.477 | -0.470 | -0.482 | -0.476 |
|  | (0.374) | (0.375) | (0.375) | (0.376) |
|  |  |  |  |  |
| 2012 | -0.0382 | -0.0775 | -0.0601 | -0.0983 |
|  | (0.399) | (0.396) | (0.398) | (0.396) |
|  |  |  |  |  |
| 2013 | 0.477 | 0.514 | 0.468 | 0.503 |
|  | (0.372) | (0.367) | (0.373) | (0.369) |
|  |  |  |  |  |
| 2014 | -0.0876 | -0.0466 | -0.0907 | -0.0509 |
|  | (0.288) | (0.292) | (0.288) | (0.293) |
|  |  |  |  |  |
| 2015 | -0.148 | -0.0697 | -0.160 | -0.0792 |
|  | (0.321) | (0.317) | (0.321) | (0.317) |
|  |  |  |  |  |
| 2016 | -0.134 | -0.0561 | -0.146 | -0.0683 |
|  | (0.318) | (0.318) | (0.318) | (0.317) |
|  |  |  |  |  |
| 2017 | -0.449 | -0.286 | -0.455 | -0.289 |
|  | (0.342) | (0.345) | (0.342) | (0.345) |
|  |  |  |  |  |
| 2018 | 0.197 | 0.318 | 0.180 | 0.304 |
|  | (0.356) | (0.350) | (0.355) | (0.349) |
|  |  |  |  |  |
| 2019 | 0.593^†^ | 0.810^*^ | 0.576^†^ | 0.797^*^ |
|  | (0.340) | (0.337) | (0.340) | (0.337) |
|  |  |  |  |  |
| MDMA | 0.0356 | 0.0573 | 0.206 | 0.215 |
|  | (0.238) | (0.237) | (0.236) | (0.236) |
|  |  |  |  |  |
| LCPU | -0.182 | -0.0839 |  |  |
|  | (0.315) | (0.313) |  |  |
|  |  |  |  |  |
| Educational Level |  | -0.339^***^ |  | -0.336^***^ |
|  |  | (0.0840) |  | (0.0834) |
|  |  |  |  |  |
| Family Income |  | -0.355^***^ |  | -0.354^***^ |
|  |  | (0.0385) |  | (0.0385) |
|  |  |  |  |  |
| Psilocybin |  |  | -0.656^*^ | -0.517 |
|  |  |  | (0.293) | (0.285) |
|  |  |  |  |  |
| DMT |  |  | -0.342 | -0.119 |
|  |  |  | (2.080) | (1.948) |
|  |  |  |  |  |
| Ayahuasca |  |  | 1.613 | 2.703 |
|  |  |  | (5.692) | (5.761) |
|  |  |  |  |  |
| Peyote/Mescaline |  |  | 0.397 | 0.482 |
|  |  |  | (0.436) | (0.422) |
|  |  |  |  |  |
| LSD |  |  | -0.278 | -0.267 |
|  |  |  | (0.372) | (0.366) |
|  |  |  |  |  |
| Constant | 13.04^***^ | 15.25^***^ | 13.06^***^ | 15.26^***^ |
|  | (0.647) | (0.663) | (0.647) | (0.663) |
| Observations | 21113 | 21113 | 21113 | 21113 |
| *R*^2^ | 0.102 | 0.121 | 0.103 | 0.121 |
| Source: 2008-2019 National Survey of Drug Use and Health, N=458,372  Standard errors in parentheses  ^a^ Men serve as the references category  ^b^ Single Serves as the reference category for marital statuses  ^c^ 2008 serves as the reference category for the year survey was taken  ^†^ *p* < 0.1, ^*^ *p* < 0.05, ^**^ *p* < 0.01, ^***^ *p* < 0.001 | | | | |

| Supplemental Table 4b. Weighted Multivariate Ordinary Squared Logistics Regression Predicting Psychological Distress Levels In the Past 30 Days Among Hispanics with Interaction Terms (Psychedelics * Educational Level) | | | | | | | |
| --- | --- | --- | --- | --- | --- | --- | --- |
|  | Model 5 | Model 6 | Model 7 | Model 8 | Model 9 | Model 10 | Model 11 |
| Age | -0.367^***^ | -0.369^***^ | -0.371^***^ | -0.369^***^ | -0.369^***^ | -0.369^***^ | -0.367^***^ |
|  | (0.0350) | (0.0348) | (0.0348) | (0.0347) | (0.0347) | (0.0347) | (0.0350) |
|  |  |  |  |  |  |  |  |
| ^a^Women | 1.040^***^ | 1.032^***^ | 1.031^***^ | 1.032^***^ | 1.032^***^ | 1.032^***^ | 1.033^***^ |
|  | (0.144) | (0.144) | (0.144) | (0.144) | (0.144) | (0.145) | (0.145) |
|  |  |  |  |  |  |  |  |
| ^b^Married | -0.715^***^ | -0.709^***^ | -0.708^***^ | -0.708^***^ | -0.709^***^ | -0.708^***^ | -0.709^***^ |
|  | (0.191) | (0.191) | (0.191) | (0.191) | (0.191) | (0.191) | (0.190) |
|  |  |  |  |  |  |  |  |
| Divorced | 0.0206 | 0.0276 | 0.0256 | 0.0281 | 0.0274 | 0.0341 | 0.0290 |
|  | (0.537) | (0.539) | (0.540) | (0.540) | (0.540) | (0.541) | (0.539) |
|  |  |  |  |  |  |  |  |
| Widowed | -0.0313 | -0.0328 | -0.0277 | -0.0309 | -0.0313 | -0.0318 | -0.0372 |
|  | (0.253) | (0.253) | (0.254) | (0.254) | (0.254) | (0.254) | (0.253) |
|  |  |  |  |  |  |  |  |
| Religious Salience | -0.0353 | -0.0365 | -0.0356 | -0.0364 | -0.0364 | -0.0368 | -0.0372 |
|  | (0.0311) | (0.0310) | (0.0310) | (0.0310) | (0.0310) | (0.0310) | (0.0311) |
|  |  |  |  |  |  |  |  |
| Religious Attendance | -0.118^**^ | -0.120^**^ | -0.119^**^ | -0.120^**^ | -0.120^**^ | -0.120^**^ | -0.120^**^ |
|  | (0.0423) | (0.0422) | (0.0421) | (0.0421) | (0.0422) | (0.0422) | (0.0421) |
|  |  |  |  |  |  |  |  |
| Cocaine | -0.176 | -0.116 | -0.122 | -0.116 | -0.117 | -0.112 | -0.115 |
|  | (0.211) | (0.215) | (0.216) | (0.216) | (0.216) | (0.215) | (0.215) |
|  |  |  |  |  |  |  |  |
| Stimulants | 1.025^***^ | 1.052^***^ | 1.038^***^ | 1.049^***^ | 1.049^***^ | 1.049^***^ | 1.062^***^ |
|  | (0.228) | (0.230) | (0.227) | (0.229) | (0.229) | (0.229) | (0.228) |
|  |  |  |  |  |  |  |  |
| Sedatives | 1.081^**^ | 1.083^**^ | 1.086^**^ | 1.081^**^ | 1.082^**^ | 1.081^**^ | 1.087^**^ |
|  | (0.324) | (0.321) | (0.323) | (0.322) | (0.322) | (0.322) | (0.321) |
|  |  |  |  |  |  |  |  |
| Tranquilizers | 1.371^***^ | 1.380^***^ | 1.383^***^ | 1.382^***^ | 1.382^***^ | 1.382^***^ | 1.375^***^ |
|  | (0.188) | (0.186) | (0.186) | (0.187) | (0.187) | (0.187) | (0.188) |
|  |  |  |  |  |  |  |  |
| Heroine | 0.0822 | 0.113 | 0.187 | 0.124 | 0.128 | 0.0902 | 0.0483 |
|  | (0.478) | (0.478) | (0.473) | (0.480) | (0.479) | (0.481) | (0.478) |
|  |  |  |  |  |  |  |  |
| Pain Killers | 0.459^**^ | 0.464^**^ | 0.470^**^ | 0.466^**^ | 0.466^**^ | 0.467^**^ | 0.459^**^ |
|  | (0.153) | (0.153) | (0.153) | (0.152) | (0.152) | (0.152) | (0.153) |
|  |  |  |  |  |  |  |  |
| Marijuana | 0.122 | 0.124 | 0.123 | 0.124 | 0.124 | 0.121 | 0.123 |
|  | (0.194) | (0.192) | (0.193) | (0.193) | (0.193) | (0.191) | (0.192) |
|  |  |  |  |  |  |  |  |
| PCP | 0.136 | 0.254 | 0.350 | 0.267 | 0.267 | 0.233 | 0.164 |
|  | (0.597) | (0.594) | (0.585) | (0.598) | (0.598) | (0.590) | (0.597) |
|  |  |  |  |  |  |  |  |
| Inhalants | 0.601^*^ | 0.654^*^ | 0.641^*^ | 0.653^*^ | 0.653^*^ | 0.652^*^ | 0.667^**^ |
|  | (0.256) | (0.250) | (0.250) | (0.250) | (0.250) | (0.250) | (0.249) |
|  |  |  |  |  |  |  |  |
| Tobacco | 0.629^***^ | 0.632^***^ | 0.634^***^ | 0.632^***^ | 0.632^***^ | 0.631^***^ | 0.632^***^ |
|  | (0.171) | (0.171) | (0.170) | (0.171) | (0.171) | (0.171) | (0.171) |
|  |  |  |  |  |  |  |  |
| Age of 1^st^ Alcohol Use | -0.107 | -0.107 | -0.110 | -0.108 | -0.108 | -0.107 | -0.105 |
|  | (0.0762) | (0.0762) | (0.0761) | (0.0758) | (0.0758) | (0.0758) | (0.0757) |
|  |  |  |  |  |  |  |  |
| Risk Behaviors | 0.907^***^ | 0.912^***^ | 0.915^***^ | 0.913^***^ | 0.913^***^ | 0.913^***^ | 0.909^***^ |
|  | (0.112) | (0.112) | (0.112) | (0.112) | (0.112) | (0.113) | (0.112) |
|  |  |  |  |  |  |  |  |
| ^c^2009 | 0.150 | 0.137 | 0.138 | 0.137 | 0.137 | 0.136 | 0.132 |
|  | (0.413) | (0.413) | (0.413) | (0.413) | (0.413) | (0.413) | (0.412) |
|  |  |  |  |  |  |  |  |
| 2010 | -0.0177 | -0.0256 | -0.0191 | -0.0246 | -0.0247 | -0.0254 | -0.0335 |
|  | (0.367) | (0.364) | (0.364) | (0.364) | (0.364) | (0.365) | (0.366) |
|  |  |  |  |  |  |  |  |
| 2011 | -0.472 | -0.476 | -0.475 | -0.476 | -0.476 | -0.480 | -0.482 |
|  | (0.374) | (0.376) | (0.377) | (0.376) | (0.376) | (0.377) | (0.375) |
|  |  |  |  |  |  |  |  |
| 2012 | -0.0828 | -0.0990 | -0.0980 | -0.0984 | -0.0983 | -0.0985 | -0.112 |
|  | (0.396) | (0.396) | (0.396) | (0.396) | (0.396) | (0.395) | (0.395) |
|  |  |  |  |  |  |  |  |
| 2013 | 0.512 | 0.501 | 0.501 | 0.502 | 0.503 | 0.503 | 0.495 |
|  | (0.366) | (0.369) | (0.369) | (0.369) | (0.369) | (0.369) | (0.367) |
|  |  |  |  |  |  |  |  |
| 2014 | -0.0507 | -0.0521 | -0.0459 | -0.0503 | -0.0509 | -0.0543 | -0.0603 |
|  | (0.293) | (0.292) | (0.293) | (0.292) | (0.293) | (0.293) | (0.293) |
|  |  |  |  |  |  |  |  |
| 2015 | -0.0721 | -0.0785 | -0.0755 | -0.0792 | -0.0792 | -0.0846 | -0.0848 |
|  | (0.317) | (0.317) | (0.317) | (0.317) | (0.317) | (0.317) | (0.317) |
|  |  |  |  |  |  |  |  |
| 2016 | -0.0563 | -0.0677 | -0.0714 | -0.0684 | -0.0683 | -0.0709 | -0.0713 |
|  | (0.317) | (0.317) | (0.318) | (0.317) | (0.317) | (0.317) | (0.315) |
|  |  |  |  |  |  |  |  |
| 2017 | -0.288 | -0.289 | -0.285 | -0.289 | -0.289 | -0.292 | -0.297 |
|  | (0.345) | (0.345) | (0.346) | (0.345) | (0.345) | (0.346) | (0.345) |
|  |  |  |  |  |  |  |  |
| 2018 | 0.314 | 0.302 | 0.308 | 0.304 | 0.304 | 0.293 | 0.296 |
|  | (0.349) | (0.349) | (0.350) | (0.349) | (0.349) | (0.348) | (0.347) |
|  |  |  |  |  |  |  |  |
| 2019 | 0.807^*^ | 0.798^*^ | 0.798^*^ | 0.797^*^ | 0.797^*^ | 0.792^*^ | 0.791^*^ |
|  | (0.337) | (0.337) | (0.338) | (0.337) | (0.337) | (0.337) | (0.337) |
|  |  |  |  |  |  |  |  |
| MDMA | 0.0602 | 0.398 | 0.227 | 0.214 | 0.215 | 0.202 | 0.218 |
|  | (0.237) | (0.643) | (0.234) | (0.236) | (0.236) | (0.234) | (0.237) |
|  |  |  |  |  |  |  |  |
| LCPU | 0.407 |  |  |  |  |  |  |
|  | (0.714) |  |  |  |  |  |  |
|  |  |  |  |  |  |  |  |
| Educational Level | -0.319^***^ | -0.330^***^ | -0.358^***^ | -0.335^***^ | -0.336^***^ | -0.325^***^ | -0.307^***^ |
|  | (0.0831) | (0.0875) | (0.0819) | (0.0834) | (0.0834) | (0.0852) | (0.0805) |
|  |  |  |  |  |  |  |  |
| Family Income | -0.356^***^ | -0.355^***^ | -0.353^***^ | -0.354^***^ | -0.354^***^ | -0.356^***^ | -0.356^***^ |
|  | (0.0382) | (0.0384) | (0.0384) | (0.0385) | (0.0385) | (0.0381) | (0.0382) |
|  |  |  |  |  |  |  |  |
| LCPU * Educational Level | -0.181 |  |  |  |  |  |  |
|  | (0.229) |  |  |  |  |  |  |
|  |  |  |  |  |  |  |  |
| Psilocybin |  | -0.516^†^ | -1.338^†^ | -0.516^†^ | -0.517^†^ | -0.497^†^ | -0.484^†^ |
|  |  | (0.285) | (0.720) | (0.285) | (0.285) | (0.282) | (0.275) |
|  |  |  |  |  |  |  |  |
| DMT |  | -0.107 | -0.176 | 1.632 | -0.119 | -0.144 | -0.0979 |
|  |  | (1.943) | (1.967) | (2.994) | (1.948) | (1.936) | (1.956) |
|  |  |  |  |  |  |  |  |
| Ayahuasca |  | 2.751 | 2.505 | 2.702 | 2.703 | 2.775 | 2.851 |
|  |  | (5.754) | (5.894) | (5.762) | (5.761) | (5.761) | (5.674) |
|  |  |  |  |  |  |  |  |
| Peyote/Mescaline |  | 0.480 | 0.465 | 0.480 | 0.482 | 1.358 | 0.504 |
|  |  | (0.421) | (0.425) | (0.423) | (0.422) | (1.300) | (0.417) |
|  |  |  |  |  |  |  |  |
| LSD |  | -0.264 | -0.272 | -0.268 | -0.267 | -0.261 | 0.666 |
|  |  | (0.366) | (0.365) | (0.366) | (0.366) | (0.367) | (0.826) |
|  |  |  |  |  |  |  |  |
| MDMA * Educational Level |  | -0.0677 |  |  |  |  |  |
|  |  | (0.219) |  |  |  |  |  |
|  |  |  |  |  |  |  |  |
| Psilocybin * Educational Level |  |  | 0.295 |  |  |  |  |
|  |  |  | (0.253) |  |  |  |  |
|  |  |  |  |  |  |  |  |
| DMT * Educational Level |  |  |  | -0.585 |  |  |  |
|  |  |  |  | (1.004) |  |  |  |
|  |  |  |  |  |  |  |  |
| Ayahuasca * Educational Level |  |  |  |  | 0 |  |  |
|  |  |  |  |  | (.) |  |  |
|  |  |  |  |  |  |  |  |
| Peyote/Mescaline * Educational Level |  |  |  |  |  | -0.333 |  |
|  |  |  |  |  |  | (0.484) |  |
|  |  |  |  |  |  |  |  |
| LSD * Educational Level |  |  |  |  |  |  | -0.354 |
|  |  |  |  |  |  |  | (0.254) |
|  |  |  |  |  |  |  |  |
| Constant | 15.18^***^ | 15.24^***^ | 15.33^***^ | 15.26^***^ | 15.26^***^ | 15.24^***^ | 15.18^***^ |
|  | (0.678) | (0.679) | (0.668) | (0.662) | (0.663) | (0.667) | (0.671) |
| Observations | 21113 | 21113 | 21113 | 21113 | 21113 | 21113 | 21113 |
| *R*^2^ | 0.121 | 0.121 | 0.121 | 0.121 | 0.121 | 0.121 | 0.121 |
| Source: 2008-2019 National Survey of Drug Use and Health, N=458,372  Standard errors in parentheses  ^a^ Men serve as the references category  ^b^ Single Serves as the reference category for marital statuses  ^c^ 2008 serves as the reference category for the year survey was taken  ^†^ *p* < 0.1, ^*^ *p* < 0.05, ^**^ *p* < 0.01, ^***^ *p* < 0.001 | | | | | | | |

| Supplemental Table 4c. Weighted Multivariate Ordinary Squared Logistics Regression Predicting Psychological Distress Levels In the Past 30 Days Among Hispanics with Interaction Terms (Psychedelics * Family Income Level) | | | | | | | |
| --- | --- | --- | --- | --- | --- | --- | --- |
|  | Model 12 | Model 13 | Model 14 | Model 15 | Model 16 | Model 17 | Model 18 |
| Age | -0.368^***^ | -0.370^***^ | -0.370^***^ | -0.369^***^ | -0.369^***^ | -0.369^***^ | -0.369^***^ |
|  | (0.0347) | (0.0347) | (0.0347) | (0.0347) | (0.0347) | (0.0347) | (0.0347) |
|  |  |  |  |  |  |  |  |
| ^a^Women | 1.039^***^ | 1.032^***^ | 1.032^***^ | 1.032^***^ | 1.031^***^ | 1.034^***^ | 1.031^***^ |
|  | (0.144) | (0.145) | (0.144) | (0.144) | (0.144) | (0.144) | (0.144) |
|  |  |  |  |  |  |  |  |
| ^b^Married | -0.718^***^ | -0.710^***^ | -0.715^***^ | -0.709^***^ | -0.710^***^ | -0.714^***^ | -0.712^***^ |
|  | (0.192) | (0.191) | (0.191) | (0.191) | (0.191) | (0.191) | (0.192) |
|  |  |  |  |  |  |  |  |
| Divorced | 0.00709 | 0.0124 | 0.00926 | 0.0274 | 0.0270 | 0.0114 | 0.0197 |
|  | (0.537) | (0.540) | (0.539) | (0.540) | (0.540) | (0.539) | (0.538) |
|  |  |  |  |  |  |  |  |
| Widowed | -0.0283 | -0.0377 | -0.0330 | -0.0313 | -0.0328 | -0.0240 | -0.0313 |
|  | (0.254) | (0.254) | (0.254) | (0.254) | (0.254) | (0.255) | (0.254) |
|  |  |  |  |  |  |  |  |
| Religious Salience | -0.0341 | -0.0357 | -0.0356 | -0.0364 | -0.0365 | -0.0356 | -0.0359 |
|  | (0.0310) | (0.0311) | (0.0311) | (0.0310) | (0.0310) | (0.0310) | (0.0311) |
|  |  |  |  |  |  |  |  |
| Religious Attendance | -0.119^**^ | -0.121^**^ | -0.120^**^ | -0.120^**^ | -0.120^**^ | -0.122^**^ | -0.121^**^ |
|  | (0.0424) | (0.0422) | (0.0421) | (0.0422) | (0.0422) | (0.0417) | (0.0422) |
|  |  |  |  |  |  |  |  |
| Cocaine | -0.180 | -0.115 | -0.126 | -0.117 | -0.117 | -0.123 | -0.118 |
|  | (0.212) | (0.217) | (0.216) | (0.216) | (0.216) | (0.217) | (0.216) |
|  |  |  |  |  |  |  |  |
| Stimulants | 1.016^***^ | 1.038^***^ | 1.036^***^ | 1.049^***^ | 1.049^***^ | 1.047^***^ | 1.047^***^ |
|  | (0.228) | (0.229) | (0.227) | (0.229) | (0.229) | (0.228) | (0.229) |
|  |  |  |  |  |  |  |  |
| Sedatives | 1.081^**^ | 1.085^**^ | 1.078^**^ | 1.082^**^ | 1.082^**^ | 1.072^**^ | 1.082^**^ |
|  | (0.323) | (0.323) | (0.322) | (0.322) | (0.322) | (0.324) | (0.322) |
|  |  |  |  |  |  |  |  |
| Tranquilizers | 1.377^***^ | 1.386^***^ | 1.395^***^ | 1.382^***^ | 1.383^***^ | 1.389^***^ | 1.386^***^ |
|  | (0.188) | (0.187) | (0.187) | (0.187) | (0.187) | (0.188) | (0.187) |
|  |  |  |  |  |  |  |  |
| Heroine | 0.165 | 0.154 | 0.168 | 0.128 | 0.128 | 0.180 | 0.153 |
|  | (0.474) | (0.478) | (0.471) | (0.479) | (0.479) | (0.478) | (0.476) |
|  |  |  |  |  |  |  |  |
| Pain Killers | 0.463^**^ | 0.471^**^ | 0.466^**^ | 0.466^**^ | 0.466^**^ | 0.469^**^ | 0.467^**^ |
|  | (0.153) | (0.153) | (0.153) | (0.153) | (0.152) | (0.153) | (0.153) |
|  |  |  |  |  |  |  |  |
| Marijuana | 0.122 | 0.127 | 0.124 | 0.124 | 0.124 | 0.123 | 0.124 |
|  | (0.194) | (0.192) | (0.192) | (0.193) | (0.193) | (0.192) | (0.192) |
|  |  |  |  |  |  |  |  |
| PCP | 0.202 | 0.304 | 0.295 | 0.267 | 0.267 | 0.283 | 0.274 |
|  | (0.602) | (0.603) | (0.604) | (0.599) | (0.598) | (0.599) | (0.603) |
|  |  |  |  |  |  |  |  |
| Inhalants | 0.586^*^ | 0.658^**^ | 0.646^*^ | 0.653^*^ | 0.656^*^ | 0.651^*^ | 0.651^*^ |
|  | (0.258) | (0.251) | (0.251) | (0.250) | (0.251) | (0.250) | (0.250) |
|  |  |  |  |  |  |  |  |
| Tobacco | 0.627^***^ | 0.626^***^ | 0.627^***^ | 0.632^***^ | 0.632^***^ | 0.626^***^ | 0.630^***^ |
|  | (0.172) | (0.171) | (0.171) | (0.171) | (0.171) | (0.169) | (0.172) |
|  |  |  |  |  |  |  |  |
| Age of 1^st^ Alcohol Use | -0.109 | -0.109 | -0.110 | -0.108 | -0.108 | -0.109 | -0.108 |
|  | (0.0759) | (0.0757) | (0.0757) | (0.0758) | (0.0758) | (0.0759) | (0.0758) |
|  |  |  |  |  |  |  |  |
| Risk Behaviors | 0.911^***^ | 0.915^***^ | 0.916^***^ | 0.913^***^ | 0.913^***^ | 0.914^***^ | 0.914^***^ |
|  | (0.113) | (0.112) | (0.112) | (0.112) | (0.113) | (0.113) | (0.112) |
|  |  |  |  |  |  |  |  |
| ^c^2009 | 0.158 | 0.142 | 0.141 | 0.137 | 0.137 | 0.150 | 0.141 |
|  | (0.413) | (0.413) | (0.413) | (0.413) | (0.413) | (0.411) | (0.412) |
|  |  |  |  |  |  |  |  |
| 2010 | -0.0154 | -0.0215 | -0.0207 | -0.0247 | -0.0245 | -0.0201 | -0.0221 |
|  | (0.366) | (0.363) | (0.363) | (0.364) | (0.364) | (0.363) | (0.364) |
|  |  |  |  |  |  |  |  |
| 2011 | -0.466 | -0.470 | -0.466 | -0.476 | -0.473 | -0.465 | -0.474 |
|  | (0.374) | (0.376) | (0.375) | (0.376) | (0.375) | (0.375) | (0.375) |
|  |  |  |  |  |  |  |  |
| 2012 | -0.0714 | -0.0924 | -0.0916 | -0.0983 | -0.0999 | -0.0901 | -0.0932 |
|  | (0.395) | (0.395) | (0.396) | (0.396) | (0.396) | (0.396) | (0.394) |
|  |  |  |  |  |  |  |  |
| 2013 | 0.519 | 0.515 | 0.507 | 0.503 | 0.502 | 0.510 | 0.507 |
|  | (0.366) | (0.369) | (0.369) | (0.369) | (0.369) | (0.367) | (0.367) |
|  |  |  |  |  |  |  |  |
| 2014 | -0.0445 | -0.0444 | -0.0466 | -0.0510 | -0.0509 | -0.0452 | -0.0495 |
|  | (0.292) | (0.293) | (0.293) | (0.292) | (0.293) | (0.293) | (0.292) |
|  |  |  |  |  |  |  |  |
| 2015 | -0.0693 | -0.0796 | -0.0794 | -0.0792 | -0.0794 | -0.0727 | -0.0795 |
|  | (0.317) | (0.317) | (0.318) | (0.317) | (0.317) | (0.317) | (0.317) |
|  |  |  |  |  |  |  |  |
| 2016 | -0.0549 | -0.0682 | -0.0659 | -0.0683 | -0.0683 | -0.0663 | -0.0681 |
|  | (0.318) | (0.318) | (0.318) | (0.317) | (0.317) | (0.317) | (0.317) |
|  |  |  |  |  |  |  |  |
| 2017 | -0.283 | -0.290 | -0.283 | -0.289 | -0.289 | -0.286 | -0.286 |
|  | (0.345) | (0.346) | (0.345) | (0.345) | (0.345) | (0.346) | (0.345) |
|  |  |  |  |  |  |  |  |
| 2018 | 0.320 | 0.304 | 0.306 | 0.304 | 0.303 | 0.309 | 0.304 |
|  | (0.350) | (0.349) | (0.350) | (0.349) | (0.350) | (0.350) | (0.350) |
|  |  |  |  |  |  |  |  |
| 2019 | 0.813^*^ | 0.792^*^ | 0.804^*^ | 0.797^*^ | 0.797^*^ | 0.807^*^ | 0.799^*^ |
|  | (0.336) | (0.337) | (0.336) | (0.337) | (0.337) | (0.336) | (0.336) |
|  |  |  |  |  |  |  |  |
| MDMA | 0.0623 | -0.498 | 0.235 | 0.215 | 0.214 | 0.231 | 0.219 |
|  | (0.238) | (0.451) | (0.237) | (0.236) | (0.236) | (0.238) | (0.237) |
|  |  |  |  |  |  |  |  |
| LCPU | -0.428 |  |  |  |  |  |  |
|  | (0.612) |  |  |  |  |  |  |
|  |  |  |  |  |  |  |  |
| Educational Level | -0.337^***^ | -0.331^***^ | -0.332^***^ | -0.336^***^ | -0.336^***^ | -0.331^***^ | -0.334^***^ |
|  | (0.0844) | (0.0834) | (0.0835) | (0.0835) | (0.0834) | (0.0826) | (0.0839) |
|  |  |  |  |  |  |  |  |
| Family Income | -0.364^***^ | -0.372^***^ | -0.370^***^ | -0.354^***^ | -0.354^***^ | -0.363^***^ | -0.361^***^ |
|  | (0.0421) | (0.0416) | (0.0416) | (0.0386) | (0.0385) | (0.0389) | (0.0411) |
|  |  |  |  |  |  |  |  |
| LCPU * Family Income | 0.0738 |  |  |  |  |  |  |
|  | (0.101) |  |  |  |  |  |  |
|  |  |  |  |  |  |  |  |
| Psilocybin |  | -0.524^†^ | -1.383^*^ | -0.517^†^ | -0.518^†^ | -0.530^†^ | -0.526^†^ |
|  |  | (0.287) | (0.666) | (0.285) | (0.285) | (0.284) | (0.284) |
|  |  |  |  |  |  |  |  |
| DMT |  | -0.227 | -0.203 | -0.191 | -0.121 | -0.196 | -0.147 |
|  |  | (1.969) | (1.968) | (2.235) | (1.947) | (1.992) | (1.951) |
|  |  |  |  |  |  |  |  |
| Ayahuasca |  | 2.537 | 2.519 | 2.703 | 25.41 | 2.689 | 2.637 |
|  |  | (5.842) | (5.876) | (5.761) | (35.55) | (5.765) | (5.807) |
|  |  |  |  |  |  |  |  |
| Peyote/Mescaline |  | 0.490 | 0.484 | 0.482 | 0.474 | -0.675 | 0.478 |
|  |  | (0.420) | (0.419) | (0.421) | (0.422) | (1.161) | (0.422) |
|  |  |  |  |  |  |  |  |
| LSD |  | -0.270 | -0.266 | -0.267 | -0.262 | -0.273 | -0.581 |
|  |  | (0.366) | (0.364) | (0.366) | (0.366) | (0.365) | (0.750) |
|  |  |  |  |  |  |  |  |
| MDMA * Family Income |  | 0.156 |  |  |  |  |  |
|  |  | (0.0968) |  |  |  |  |  |
|  |  |  |  |  |  |  |  |
| Psilocybin * Family Income |  |  | 0.183 |  |  |  |  |
|  |  |  | (0.124) |  |  |  |  |
|  |  |  |  |  |  |  |  |
| DMT * Family Income |  |  |  | 0.0141 |  |  |  |
|  |  |  |  | (0.596) |  |  |  |
|  |  |  |  |  |  |  |  |
| Ayahuasca * Family Income |  |  |  |  | -3.638 |  |  |
|  |  |  |  |  | (5.334) |  |  |
|  |  |  |  |  |  |  |  |
| Peyote/Mescaline * Family Income |  |  |  |  |  | 0.254 |  |
|  |  |  |  |  |  | (0.224) |  |
|  |  |  |  |  |  |  |  |
| LSD * Family Income |  |  |  |  |  |  | 0.0693 |
|  |  |  |  |  |  |  | (0.126) |
|  |  |  |  |  |  |  |  |
| Constant | 15.28^***^ | 15.34^***^ | 15.33^***^ | 15.27^***^ | 15.26^***^ | 15.28^***^ | 15.29^***^ |
|  | (0.669) | (0.663) | (0.665) | (0.662) | (0.662) | (0.665) | (0.666) |
| Observations | 21113 | 21113 | 21113 | 21113 | 21113 | 21113 | 21113 |
| *R*^2^ | 0.121 | 0.121 | 0.121 | 0.121 | 0.121 | 0.121 | 0.121 |
| Source: 2008-2019 National Survey of Drug Use and Health, N=458,372  Standard errors in parentheses  ^a^ Men serve as the references category  ^b^ Single Serves as the reference category for marital statuses  ^c^ 2008 serves as the reference category for the year survey was taken  ^†^ *p* < 0.1, ^*^ *p* < 0.05, ^**^ *p* < 0.01, ^***^ *p* < 0.001 | | | | | | | |

| Supplemental Table 5a. Weighted Multivariate Ordinary Squared Logistics Regression Predicting Psychological Distress Levels In the Past 30 Days Among Asians | | | | |
| --- | --- | --- | --- | --- |
|  | Model 1 | Model 2 | Model 3 | Model 4 |
| Age | -0.433^***^ | -0.371^***^ | -0.434^***^ | -0.372^***^ |
|  | (0.0654) | (0.0687) | (0.0654) | (0.0685) |
|  |  |  |  |  |
| ^a^Women | 0.563^*^ | 0.593^**^ | 0.578^**^ | 0.609^**^ |
|  | (0.215) | (0.217) | (0.214) | (0.217) |
|  |  |  |  |  |
| ^b^Married | -1.751^***^ | -1.523^***^ | -1.738^***^ | -1.512^***^ |
|  | (0.349) | (0.356) | (0.345) | (0.353) |
|  |  |  |  |  |
| Divorced | -0.0686 | -0.176 | -0.0587 | -0.171 |
|  | (0.855) | (0.899) | (0.857) | (0.901) |
|  |  |  |  |  |
| Widowed | -1.332^*^ | -1.424^*^ | -1.300^*^ | -1.385^*^ |
|  | (0.552) | (0.548) | (0.556) | (0.553) |
|  |  |  |  |  |
| Religious Salience | 0.0559 | 0.0289 | 0.0576 | 0.0304 |
|  | (0.0394) | (0.0404) | (0.0400) | (0.0410) |
|  |  |  |  |  |
| Religious Attendance | -0.0932 | -0.0626 | -0.0973 | -0.0660 |
|  | (0.0605) | (0.0620) | (0.0604) | (0.0621) |
|  |  |  |  |  |
| Cocaine | 0.749 | 0.661 | 0.741 | 0.670 |
|  | (0.501) | (0.502) | (0.512) | (0.513) |
|  |  |  |  |  |
| Stimulants | 0.180 | 0.203 | 0.192 | 0.212 |
|  | (0.367) | (0.370) | (0.364) | (0.367) |
|  |  |  |  |  |
| Sedatives | 1.407^*^ | 1.373^*^ | 1.397^*^ | 1.368^*^ |
|  | (0.625) | (0.625) | (0.630) | (0.629) |
|  |  |  |  |  |
| Tranquilizers | 0.604 | 0.535 | 0.582 | 0.513 |
|  | (0.409) | (0.412) | (0.402) | (0.404) |
|  |  |  |  |  |
| Heroine | -2.760 | -2.595 | -2.787 | -2.588 |
|  | (2.040) | (1.997) | (2.053) | (2.017) |
|  |  |  |  |  |
| Pain Killers | 0.454^†^ | 0.461^†^ | 0.436^†^ | 0.443^†^ |
|  | (0.239) | (0.241) | (0.237) | (0.239) |
|  |  |  |  |  |
| Marijuana | 0.265 | 0.303 | 0.289 | 0.327 |
|  | (0.282) | (0.281) | (0.285) | (0.284) |
|  |  |  |  |  |
| PCP | -0.183 | -0.338 | -0.0767 | -0.191 |
|  | (1.250) | (1.256) | (1.252) | (1.262) |
|  |  |  |  |  |
| Inhalants | 0.745^†^ | 0.687^†^ | 0.745^†^ | 0.686^†^ |
|  | (0.380) | (0.377) | (0.391) | (0.388) |
|  |  |  |  |  |
| Tobacco | 0.410 | 0.390 | 0.418 | 0.397 |
|  | (0.280) | (0.280) | (0.278) | (0.278) |
|  |  |  |  |  |
| Age of 1^st^ Alcohol Use | 0.0274 | 0.00363 | 0.0293 | 0.00557 |
|  | (0.113) | (0.113) | (0.114) | (0.114) |
|  |  |  |  |  |
| Risk Behaviors | 0.608^***^ | 0.660^***^ | 0.611^***^ | 0.663^***^ |
|  | (0.159) | (0.164) | (0.160) | (0.164) |
|  |  |  |  |  |
| ^c^2009 | -0.688 | -0.612 | -0.676 | -0.605 |
|  | (0.466) | (0.483) | (0.469) | (0.482) |
|  |  |  |  |  |
| 2010 | -1.125^†^ | -1.059^†^ | -1.113^†^ | -1.052^†^ |
|  | (0.614) | (0.600) | (0.619) | (0.605) |
|  |  |  |  |  |
| 2011 | -0.0812 | -0.0512 | -0.0783 | -0.0568 |
|  | (0.571) | (0.565) | (0.582) | (0.577) |
|  |  |  |  |  |
| 2012 | -0.194 | -0.134 | -0.224 | -0.170 |
|  | (0.528) | (0.530) | (0.554) | (0.554) |
|  |  |  |  |  |
| 2013 | -0.261 | -0.165 | -0.254 | -0.161 |
|  | (0.608) | (0.596) | (0.619) | (0.608) |
|  |  |  |  |  |
| 2014 | -0.844^†^ | -0.737 | -0.832^†^ | -0.729 |
|  | (0.495) | (0.487) | (0.500) | (0.491) |
|  |  |  |  |  |
| 2015 | -0.225 | -0.0905 | -0.193 | -0.0648 |
|  | (0.557) | (0.551) | (0.561) | (0.555) |
|  |  |  |  |  |
| 2016 | -1.385^*^ | -1.261^*^ | -1.366^*^ | -1.248^*^ |
|  | (0.536) | (0.525) | (0.537) | (0.526) |
|  |  |  |  |  |
| 2017 | -0.502 | -0.354 | -0.480 | -0.336 |
|  | (0.503) | (0.497) | (0.501) | (0.494) |
|  |  |  |  |  |
| 2018 | -0.639 | -0.491 | -0.613 | -0.470 |
|  | (0.511) | (0.506) | (0.516) | (0.510) |
|  |  |  |  |  |
| 2019 | -0.207 | -0.0371 | -0.183 | -0.0199 |
|  | (0.550) | (0.547) | (0.551) | (0.547) |
|  |  |  |  |  |
| MDMA | -0.269 | -0.241 | -0.298 | -0.268 |
|  | (0.480) | (0.485) | (0.490) | (0.493) |
|  |  |  |  |  |
| LCPU | 0.618 | 0.612 |  |  |
|  | (0.597) | (0.600) |  |  |
|  |  |  |  |  |
| Educational Level |  | -0.229 |  | -0.236 |
|  |  | (0.155) |  | (0.152) |
|  |  |  |  |  |
| Family Income |  | -0.192^**^ |  | -0.189^**^ |
|  |  | (0.0571) |  | (0.0569) |
|  |  |  |  |  |
| Psilocybin |  |  | 0.528 | 0.587 |
|  |  |  | (0.730) | (0.730) |
|  |  |  |  |  |
| DMT |  |  | 8.681^**^ | 8.540^**^ |
|  |  |  | (2.630) | (2.715) |
|  |  |  |  |  |
| Ayahuasca |  |  | -2.947^***^ | -3.469^***^ |
|  |  |  | (0.721) | (0.754) |
|  |  |  |  |  |
| Peyote/Mescaline |  |  | 0.173 | 0.0448 |
|  |  |  | (0.960) | (0.987) |
|  |  |  |  |  |
| LSD |  |  | 0.119 | 0.0361 |
|  |  |  | (0.749) | (0.751) |
|  |  |  |  |  |
| Constant | 14.49^***^ | 15.30^***^ | 14.46^***^ | 15.29^***^ |
|  | (0.882) | (0.912) | (0.889) | (0.919) |
| Observations | 5886 | 5886 | 5886 | 5886 |
| *R*^2^ | 0.140 | 0.146 | 0.141 | 0.147 |
| Source: 2008-2019 National Survey of Drug Use and Health, N=458,372  Standard errors in parentheses  ^a^ Men serve as the references category  ^b^ Single Serves as the reference category for marital statuses  ^c^ 2008 serves as the reference category for the year survey was taken  ^†^ *p* < 0.1, ^*^ *p* < 0.05, ^**^ *p* < 0.01, ^***^ *p* < 0.001 | | | | |

| Supplemental Table 5b. Weighted Multivariate Ordinary Squared Logistics Regression Predicting Psychological Distress Levels In the Past 30 Days Among Asians with Interaction Terms (Psychedelics * Educational Level) | | | | | | | |
| --- | --- | --- | --- | --- | --- | --- | --- |
|  | Model 5 | Model 6 | Model 7 | Model 8 | Model 9 | Model 10 | Model 11 |
| Age | -0.372^***^ | -0.373^***^ | -0.372^***^ | -0.372^***^ | -0.372^***^ | -0.371^***^ | -0.374^***^ |
|  | (0.0686) | (0.0685) | (0.0685) | (0.0685) | (0.0685) | (0.0685) | (0.0688) |
|  |  |  |  |  |  |  |  |
| ^a^Women | 0.587^**^ | 0.606^**^ | 0.610^**^ | 0.609^**^ | 0.609^**^ | 0.609^**^ | 0.598^**^ |
|  | (0.219) | (0.217) | (0.217) | (0.217) | (0.217) | (0.217) | (0.218) |
|  |  |  |  |  |  |  |  |
| ^b^Married | -1.519^***^ | -1.506^***^ | -1.510^***^ | -1.509^***^ | -1.512^***^ | -1.513^***^ | -1.509^***^ |
|  | (0.358) | (0.352) | (0.354) | (0.353) | (0.353) | (0.354) | (0.355) |
|  |  |  |  |  |  |  |  |
| Divorced | -0.152 | -0.175 | -0.160 | -0.169 | -0.171 | -0.174 | -0.141 |
|  | (0.898) | (0.903) | (0.901) | (0.901) | (0.901) | (0.902) | (0.898) |
|  |  |  |  |  |  |  |  |
| Widowed | -1.439^**^ | -1.375^*^ | -1.388^*^ | -1.383^*^ | -1.385^*^ | -1.382^*^ | -1.396^*^ |
|  | (0.548) | (0.553) | (0.553) | (0.553) | (0.553) | (0.555) | (0.552) |
|  |  |  |  |  |  |  |  |
| Religious Salience | 0.0294 | 0.0313 | 0.0301 | 0.0305 | 0.0304 | 0.0306 | 0.0316 |
|  | (0.0403) | (0.0411) | (0.0411) | (0.0411) | (0.0410) | (0.0411) | (0.0408) |
|  |  |  |  |  |  |  |  |
| Religious Attendance | -0.0641 | -0.0648 | -0.0663 | -0.0664 | -0.0660 | -0.0656 | -0.0680 |
|  | (0.0620) | (0.0621) | (0.0621) | (0.0621) | (0.0621) | (0.0620) | (0.0620) |
|  |  |  |  |  |  |  |  |
| Cocaine | 0.592 | 0.681 | 0.629 | 0.662 | 0.670 | 0.676 | 0.610 |
|  | (0.503) | (0.511) | (0.509) | (0.513) | (0.513) | (0.516) | (0.507) |
|  |  |  |  |  |  |  |  |
| Stimulants | 0.238 | 0.202 | 0.247 | 0.218 | 0.212 | 0.208 | 0.249 |
|  | (0.371) | (0.367) | (0.364) | (0.367) | (0.367) | (0.369) | (0.365) |
|  |  |  |  |  |  |  |  |
| Sedatives | 1.348^*^ | 1.396^*^ | 1.331^*^ | 1.368^*^ | 1.368^*^ | 1.383^*^ | 1.342^*^ |
|  | (0.629) | (0.630) | (0.633) | (0.628) | (0.629) | (0.625) | (0.633) |
|  |  |  |  |  |  |  |  |
| Tranquilizers | 0.535 | 0.532 | 0.506 | 0.511 | 0.513 | 0.513 | 0.500 |
|  | (0.412) | (0.403) | (0.405) | (0.404) | (0.404) | (0.404) | (0.405) |
|  |  |  |  |  |  |  |  |
| Heroine | -2.358 | -2.737 | -2.303 | -2.589 | -2.588 | -2.671 | -2.187 |
|  | (1.986) | (2.009) | (2.002) | (2.016) | (2.017) | (2.069) | (2.009) |
|  |  |  |  |  |  |  |  |
| Pain Killers | 0.442^†^ | 0.448^†^ | 0.430^†^ | 0.442^†^ | 0.443^†^ | 0.446^†^ | 0.422^†^ |
|  | (0.243) | (0.240) | (0.239) | (0.239) | (0.239) | (0.240) | (0.238) |
|  |  |  |  |  |  |  |  |
| Marijuana | 0.310 | 0.323 | 0.336 | 0.327 | 0.327 | 0.325 | 0.333 |
|  | (0.282) | (0.285) | (0.284) | (0.284) | (0.284) | (0.283) | (0.285) |
|  |  |  |  |  |  |  |  |
| PCP | -0.480 | -0.266 | -0.118 | -0.191 | -0.191 | -0.233 | -0.402 |
|  | (1.253) | (1.275) | (1.280) | (1.261) | (1.262) | (1.260) | (1.250) |
|  |  |  |  |  |  |  |  |
| Inhalants | 0.689^†^ | 0.684^†^ | 0.684^†^ | 0.691^†^ | 0.686^†^ | 0.680^†^ | 0.682^†^ |
|  | (0.381) | (0.385) | (0.390) | (0.388) | (0.388) | (0.389) | (0.392) |
|  |  |  |  |  |  |  |  |
| Tobacco | 0.388 | 0.401 | 0.400 | 0.397 | 0.397 | 0.398 | 0.392 |
|  | (0.280) | (0.278) | (0.278) | (0.278) | (0.278) | (0.278) | (0.278) |
|  |  |  |  |  |  |  |  |
| Age of 1^st^ Alcohol Use | 0.00361 | 0.00365 | 0.00619 | 0.00561 | 0.00557 | 0.00522 | 0.00557 |
|  | (0.113) | (0.114) | (0.114) | (0.114) | (0.114) | (0.114) | (0.114) |
|  |  |  |  |  |  |  |  |
| Risk Behaviors | 0.664^***^ | 0.661^***^ | 0.667^***^ | 0.664^***^ | 0.663^***^ | 0.663^***^ | 0.668^***^ |
|  | (0.164) | (0.164) | (0.164) | (0.164) | (0.164) | (0.164) | (0.163) |
|  |  |  |  |  |  |  |  |
| ^c^2009 | -0.588 | -0.615 | -0.590 | -0.606 | -0.605 | -0.613 | -0.585 |
|  | (0.488) | (0.483) | (0.487) | (0.482) | (0.482) | (0.479) | (0.486) |
|  |  |  |  |  |  |  |  |
| 2010 | -1.042^†^ | -1.062^†^ | -1.040^†^ | -1.051^†^ | -1.052^†^ | -1.061^†^ | -1.039^†^ |
|  | (0.599) | (0.606) | (0.602) | (0.605) | (0.605) | (0.611) | (0.603) |
|  |  |  |  |  |  |  |  |
| 2011 | -0.0286 | -0.0636 | -0.0468 | -0.0544 | -0.0568 | -0.0642 | -0.0316 |
|  | (0.564) | (0.577) | (0.575) | (0.577) | (0.577) | (0.581) | (0.573) |
|  |  |  |  |  |  |  |  |
| 2012 | -0.121 | -0.166 | -0.166 | -0.175 | -0.170 | -0.179 | -0.161 |
|  | (0.530) | (0.556) | (0.555) | (0.556) | (0.554) | (0.556) | (0.555) |
|  |  |  |  |  |  |  |  |
| 2013 | -0.140 | -0.179 | -0.139 | -0.158 | -0.161 | -0.167 | -0.131 |
|  | (0.590) | (0.614) | (0.602) | (0.608) | (0.608) | (0.613) | (0.598) |
|  |  |  |  |  |  |  |  |
| 2014 | -0.717 | -0.747 | -0.712 | -0.729 | -0.729 | -0.736 | -0.716 |
|  | (0.486) | (0.492) | (0.491) | (0.491) | (0.491) | (0.493) | (0.490) |
|  |  |  |  |  |  |  |  |
| 2015 | -0.0659 | -0.0793 | -0.0419 | -0.0647 | -0.0648 | -0.0731 | -0.0402 |
|  | (0.551) | (0.559) | (0.555) | (0.555) | (0.555) | (0.559) | (0.555) |
|  |  |  |  |  |  |  |  |
| 2016 | -1.224^*^ | -1.278^*^ | -1.219^*^ | -1.248^*^ | -1.248^*^ | -1.258^*^ | -1.202^*^ |
|  | (0.528) | (0.528) | (0.528) | (0.526) | (0.526) | (0.529) | (0.528) |
|  |  |  |  |  |  |  |  |
| 2017 | -0.331 | -0.352 | -0.314 | -0.336 | -0.336 | -0.341 | -0.303 |
|  | (0.500) | (0.498) | (0.496) | (0.494) | (0.494) | (0.496) | (0.498) |
|  |  |  |  |  |  |  |  |
| 2018 | -0.470 | -0.489 | -0.447 | -0.470 | -0.470 | -0.480 | -0.450 |
|  | (0.508) | (0.515) | (0.513) | (0.510) | (0.510) | (0.514) | (0.511) |
|  |  |  |  |  |  |  |  |
| 2019 | -0.00885 | -0.0453 | 0.00202 | -0.0197 | -0.0199 | -0.0275 | 0.0120 |
|  | (0.551) | (0.552) | (0.548) | (0.547) | (0.547) | (0.549) | (0.550) |
|  |  |  |  |  |  |  |  |
| MDMA | -0.222 | -1.668 | -0.271 | -0.266 | -0.268 | -0.259 | -0.264 |
|  | (0.482) | (1.601) | (0.490) | (0.494) | (0.493) | (0.487) | (0.488) |
|  |  |  |  |  |  |  |  |
| LCPU | 2.395 |  |  |  |  |  |  |
|  | (1.622) |  |  |  |  |  |  |
|  |  |  |  |  |  |  |  |
| Educational Level | -0.193 | -0.267^†^ | -0.215 | -0.238 | -0.236 | -0.241 | -0.199 |
|  | (0.159) | (0.157) | (0.156) | (0.152) | (0.152) | (0.154) | (0.156) |
|  |  |  |  |  |  |  |  |
| Family Income | -0.188^**^ | -0.191^**^ | -0.186^**^ | -0.189^**^ | -0.189^**^ | -0.190^**^ | -0.184^**^ |
|  | (0.0577) | (0.0569) | (0.0568) | (0.0569) | (0.0569) | (0.0571) | (0.0571) |
|  |  |  |  |  |  |  |  |
| LCPU * Educational Level | -0.523 |  |  |  |  |  |  |
|  | (0.458) |  |  |  |  |  |  |
|  |  |  |  |  |  |  |  |
| Psilocybin |  | 0.556 | 2.478 | 0.585 | 0.587 | 0.589 | 0.632 |
|  |  | (0.723) | (2.005) | (0.730) | (0.730) | (0.729) | (0.730) |
|  |  |  |  |  |  |  |  |
| DMT |  | 8.353^**^ | 8.769^**^ | -8.630 | 8.540^**^ | 8.522^**^ | 8.858^**^ |
|  |  | (2.647) | (2.808) | (6.757) | (2.715) | (2.712) | (2.903) |
|  |  |  |  |  |  |  |  |
| Ayahuasca |  | -3.468^***^ | -3.438^***^ | -3.464^***^ | -3.469^***^ | -3.475^***^ | -3.434^***^ |
|  |  | (0.753) | (0.749) | (0.754) | (0.754) | (0.756) | (0.747) |
|  |  |  |  |  |  |  |  |
| Peyote/Mescaline |  | 0.134 | -0.0652 | 0.0465 | 0.0448 | -0.915 | -0.0353 |
|  |  | (0.983) | (1.029) | (0.986) | (0.987) | (2.810) | (1.043) |
|  |  |  |  |  |  |  |  |
| LSD |  | 0.0691 | -0.0180 | 0.0395 | 0.0361 | 0.0420 | 2.601 |
|  |  | (0.747) | (0.758) | (0.751) | (0.751) | (0.750) | (2.025) |
|  |  |  |  |  |  |  |  |
| MDMA * Educational Level |  | 0.415 |  |  |  |  |  |
|  |  | (0.451) |  |  |  |  |  |
|  |  |  |  |  |  |  |  |
| Psilocybin * Educational Level |  |  | -0.540 |  |  |  |  |
|  |  |  | (0.515) |  |  |  |  |
|  |  |  |  |  |  |  |  |
| DMT * Educational Level |  |  |  | 4.579^*^ |  |  |  |
|  |  |  |  | (2.097) |  |  |  |
|  |  |  |  |  |  |  |  |
| Ayahuasca * Educational Level |  |  |  |  | 0 |  |  |
|  |  |  |  |  | (.) |  |  |
|  |  |  |  |  |  |  |  |
| Peyote/Mescaline * Educational Level |  |  |  |  |  | 0.309 |  |
|  |  |  |  |  |  | (0.883) |  |
|  |  |  |  |  |  |  |  |
| LSD * Educational Level |  |  |  |  |  |  | -0.774 |
|  |  |  |  |  |  |  | (0.580) |
|  |  |  |  |  |  |  |  |
| Constant | 15.15^***^ | 15.44^***^ | 15.18^***^ | 15.30^***^ | 15.29^***^ | 15.31^***^ | 15.15^***^ |
|  | (0.904) | (0.926) | (0.929) | (0.920) | (0.919) | (0.932) | (0.901) |
| Observations | 5886 | 5886 | 5886 | 5886 | 5886 | 5886 | 5886 |
| *R*^2^ | 0.146 | 0.147 | 0.147 | 0.147 | 0.147 | 0.147 | 0.148 |
| Source: 2008-2019 National Survey of Drug Use and Health, N=458,372  Standard errors in parentheses  ^a^ Men serve as the references category  ^b^ Single Serves as the reference category for marital statuses  ^c^ 2008 serves as the reference category for the year survey was taken  ^†^ *p* < 0.1, ^*^ *p* < 0.05, ^**^ *p* < 0.01, ^***^ *p* < 0.001 | | | | | | | |

| Supplemental Table 5C. Weighted Multivariate Ordinary Squared Logistics Regression Predicting Psychological Distress Levels In the Past 30 Days Among Asians with Interaction Terms (Psychedelics * Family Income Level) | | | | | | | |
| --- | --- | --- | --- | --- | --- | --- | --- |
|  | Model 12 | Model 13 | Model 14 | Model 15 | Model 16 | Model 17 | Model 18 |
| Age | -0.372^***^ | -0.371^***^ | -0.372^***^ | -0.373^***^ | -0.372^***^ | -0.371^***^ | -0.375^***^ |
|  | (0.0685) | (0.0685) | (0.0684) | (0.0685) | (0.0685) | (0.0684) | (0.0680) |
|  |  |  |  |  |  |  |  |
| ^a^Women | 0.591^**^ | 0.612^**^ | 0.609^**^ | 0.609^**^ | 0.609^**^ | 0.611^**^ | 0.601^**^ |
|  | (0.218) | (0.217) | (0.217) | (0.217) | (0.217) | (0.215) | (0.217) |
|  |  |  |  |  |  |  |  |
| ^b^Married | -1.522^***^ | -1.509^***^ | -1.512^***^ | -1.507^***^ | -1.512^***^ | -1.516^***^ | -1.511^***^ |
|  | (0.356) | (0.354) | (0.354) | (0.354) | (0.353) | (0.354) | (0.353) |
|  |  |  |  |  |  |  |  |
| Divorced | -0.165 | -0.171 | -0.170 | -0.165 | -0.171 | -0.185 | -0.139 |
|  | (0.897) | (0.902) | (0.901) | (0.902) | (0.901) | (0.905) | (0.894) |
|  |  |  |  |  |  |  |  |
| Widowed | -1.426^*^ | -1.384^*^ | -1.386^*^ | -1.379^*^ | -1.385^*^ | -1.379^*^ | -1.371^*^ |
|  | (0.547) | (0.553) | (0.552) | (0.553) | (0.553) | (0.557) | (0.548) |
|  |  |  |  |  |  |  |  |
| Religious Salience | 0.0290 | 0.0300 | 0.0302 | 0.0306 | 0.0304 | 0.0301 | 0.0300 |
|  | (0.0404) | (0.0409) | (0.0411) | (0.0411) | (0.0410) | (0.0411) | (0.0412) |
|  |  |  |  |  |  |  |  |
| Religious Attendance | -0.0634 | -0.0654 | -0.0660 | -0.0665 | -0.0660 | -0.0634 | -0.0668 |
|  | (0.0621) | (0.0621) | (0.0621) | (0.0621) | (0.0621) | (0.0620) | (0.0622) |
|  |  |  |  |  |  |  |  |
| Cocaine | 0.616 | 0.682 | 0.665 | 0.657 | 0.670 | 0.672 | 0.620 |
|  | (0.498) | (0.511) | (0.513) | (0.512) | (0.513) | (0.510) | (0.502) |
|  |  |  |  |  |  |  |  |
| Stimulants | 0.201 | 0.215 | 0.212 | 0.229 | 0.212 | 0.192 | 0.228 |
|  | (0.368) | (0.367) | (0.366) | (0.368) | (0.367) | (0.370) | (0.367) |
|  |  |  |  |  |  |  |  |
| Sedatives | 1.357^*^ | 1.374^*^ | 1.363^*^ | 1.366^*^ | 1.368^*^ | 1.476^*^ | 1.336^*^ |
|  | (0.625) | (0.631) | (0.629) | (0.629) | (0.629) | (0.634) | (0.630) |
|  |  |  |  |  |  |  |  |
| Tranquilizers | 0.510 | 0.523 | 0.508 | 0.515 | 0.513 | 0.542 | 0.442 |
|  | (0.413) | (0.403) | (0.405) | (0.404) | (0.404) | (0.404) | (0.409) |
|  |  |  |  |  |  |  |  |
| Heroine | -2.447 | -2.660 | -2.542 | -2.581 | -2.588 | -2.834 | -2.081 |
|  | (1.935) | (2.021) | (1.979) | (2.013) | (2.017) | (2.001) | (1.856) |
|  |  |  |  |  |  |  |  |
| Pain Killers | 0.451^†^ | 0.451^†^ | 0.441^†^ | 0.444^†^ | 0.443^†^ | 0.452^†^ | 0.405^†^ |
|  | (0.242) | (0.238) | (0.240) | (0.239) | (0.239) | (0.241) | (0.242) |
|  |  |  |  |  |  |  |  |
| Marijuana | 0.310 | 0.323 | 0.328 | 0.327 | 0.327 | 0.320 | 0.338 |
|  | (0.281) | (0.285) | (0.284) | (0.284) | (0.284) | (0.282) | (0.284) |
|  |  |  |  |  |  |  |  |
| PCP | -0.377 | -0.224 | -0.183 | -0.182 | -0.191 | -0.408 | -0.318 |
|  | (1.270) | (1.267) | (1.262) | (1.260) | (1.262) | (1.259) | (1.303) |
|  |  |  |  |  |  |  |  |
| Inhalants | 0.695^†^ | 0.691^†^ | 0.689^†^ | 0.683^†^ | 0.686^†^ | 0.600 | 0.682^†^ |
|  | (0.377) | (0.387) | (0.389) | (0.388) | (0.388) | (0.390) | (0.389) |
|  |  |  |  |  |  |  |  |
| Tobacco | 0.396 | 0.397 | 0.399 | 0.398 | 0.397 | 0.390 | 0.405 |
|  | (0.280) | (0.278) | (0.277) | (0.278) | (0.278) | (0.277) | (0.279) |
|  |  |  |  |  |  |  |  |
| Age of 1^st^ Alcohol Use | 0.00405 | 0.00583 | 0.00564 | 0.00560 | 0.00557 | 0.00741 | 0.00660 |
|  | (0.113) | (0.114) | (0.114) | (0.114) | (0.114) | (0.114) | (0.113) |
|  |  |  |  |  |  |  |  |
| Risk Behaviors | 0.663^***^ | 0.665^***^ | 0.664^***^ | 0.664^***^ | 0.663^***^ | 0.658^***^ | 0.667^***^ |
|  | (0.164) | (0.164) | (0.164) | (0.164) | (0.164) | (0.165) | (0.163) |
|  |  |  |  |  |  |  |  |
| ^c^2009 | -0.597 | -0.610 | -0.602 | -0.605 | -0.605 | -0.657 | -0.569 |
|  | (0.488) | (0.484) | (0.485) | (0.482) | (0.482) | (0.475) | (0.488) |
|  |  |  |  |  |  |  |  |
| 2010 | -1.046^†^ | -1.059^†^ | -1.049^†^ | -1.052^†^ | -1.052^†^ | -1.114^†^ | -1.026^†^ |
|  | (0.600) | (0.609) | (0.608) | (0.605) | (0.605) | (0.622) | (0.602) |
|  |  |  |  |  |  |  |  |
| 2011 | -0.0370 | -0.0623 | -0.0538 | -0.0581 | -0.0568 | -0.103 | -0.0228 |
|  | (0.566) | (0.579) | (0.580) | (0.576) | (0.577) | (0.586) | (0.576) |
|  |  |  |  |  |  |  |  |
| 2012 | -0.128 | -0.171 | -0.168 | -0.175 | -0.170 | -0.215 | -0.140 |
|  | (0.531) | (0.555) | (0.556) | (0.556) | (0.554) | (0.557) | (0.553) |
|  |  |  |  |  |  |  |  |
| 2013 | -0.140 | -0.169 | -0.155 | -0.154 | -0.161 | -0.218 | -0.0895 |
|  | (0.596) | (0.612) | (0.612) | (0.608) | (0.608) | (0.630) | (0.602) |
|  |  |  |  |  |  |  |  |
| 2014 | -0.719 | -0.738 | -0.725 | -0.729 | -0.729 | -0.778 | -0.678 |
|  | (0.490) | (0.493) | (0.496) | (0.491) | (0.491) | (0.499) | (0.492) |
|  |  |  |  |  |  |  |  |
| 2015 | -0.0631 | -0.0795 | -0.0588 | -0.0661 | -0.0648 | -0.146 | 0.00418 |
|  | (0.555) | (0.562) | (0.560) | (0.556) | (0.555) | (0.569) | (0.560) |
|  |  |  |  |  |  |  |  |
| 2016 | -1.234^*^ | -1.265^*^ | -1.242^*^ | -1.248^*^ | -1.248^*^ | -1.314^*^ | -1.174^*^ |
|  | (0.531) | (0.530) | (0.533) | (0.526) | (0.526) | (0.536) | (0.532) |
|  |  |  |  |  |  |  |  |
| 2017 | -0.327 | -0.349 | -0.330 | -0.336 | -0.336 | -0.387 | -0.248 |
|  | (0.500) | (0.500) | (0.500) | (0.494) | (0.494) | (0.506) | (0.498) |
|  |  |  |  |  |  |  |  |
| 2018 | -0.468 | -0.483 | -0.465 | -0.470 | -0.470 | -0.531 | -0.406 |
|  | (0.509) | (0.517) | (0.517) | (0.510) | (0.510) | (0.525) | (0.512) |
|  |  |  |  |  |  |  |  |
| 2019 | -0.0145 | -0.0331 | -0.0147 | -0.0201 | -0.0199 | -0.0742 | 0.0444 |
|  | (0.553) | (0.551) | (0.553) | (0.547) | (0.547) | (0.558) | (0.552) |
|  |  |  |  |  |  |  |  |
| MDMA | -0.254 | -0.728 | -0.270 | -0.263 | -0.268 | -0.234 | -0.286 |
|  | (0.485) | (0.909) | (0.493) | (0.493) | (0.493) | (0.490) | (0.484) |
|  |  |  |  |  |  |  |  |
| LCPU | 1.608 |  |  |  |  |  |  |
|  | (1.240) |  |  |  |  |  |  |
|  |  |  |  |  |  |  |  |
| Educational Level | -0.221 | -0.239 | -0.235 | -0.237 | -0.236 | -0.244 | -0.215 |
|  | (0.153) | (0.152) | (0.151) | (0.152) | (0.152) | (0.151) | (0.151) |
|  |  |  |  |  |  |  |  |
| Family Income | -0.180^**^ | -0.196^**^ | -0.187^**^ | -0.190^**^ | -0.189^**^ | -0.198^***^ | -0.164^**^ |
|  | (0.0604) | (0.0609) | (0.0596) | (0.0570) | (0.0569) | (0.0581) | (0.0586) |
|  |  |  |  |  |  |  |  |
| LCPU * Family Income | -0.179 |  |  |  |  |  |  |
|  | (0.191) |  |  |  |  |  |  |
|  |  |  |  |  |  |  |  |
| Psilocybin |  | 0.583 | 0.835 | 0.592 | 0.587 | 0.646 | 0.593 |
|  |  | (0.730) | (1.400) | (0.731) | (0.730) | (0.727) | (0.719) |
|  |  |  |  |  |  |  |  |
| DMT |  | 8.667^**^ | 8.464^**^ | -2.157 | 8.540^**^ | 8.364^**^ | 7.816^**^ |
|  |  | (2.716) | (2.813) | (3.000) | (2.715) | (2.702) | (2.860) |
|  |  |  |  |  |  |  |  |
| Ayahuasca |  | -3.508^***^ | -3.458^***^ | -3.465^***^ | -3.469^***^ | -3.487^***^ | -3.370^***^ |
|  |  | (0.751) | (0.756) | (0.754) | (0.754) | (0.753) | (0.742) |
|  |  |  |  |  |  |  |  |
| Peyote/Mescaline |  | 0.0745 | 0.0275 | 0.0510 | 0.0448 | -3.616^*^ | -0.110 |
|  |  | (0.978) | (0.990) | (0.986) | (0.987) | (1.653) | (1.067) |
|  |  |  |  |  |  |  |  |
| LSD |  | 0.0439 | 0.0297 | 0.0237 | 0.0361 | 0.102 | 3.001^*^ |
|  |  | (0.749) | (0.749) | (0.750) | (0.751) | (0.751) | (1.442) |
|  |  |  |  |  |  |  |  |
| MDMA * Family Income |  | 0.0853 |  |  |  |  |  |
|  |  | (0.149) |  |  |  |  |  |
|  |  |  |  |  |  |  |  |
| Psilocybin * Family Income |  |  | -0.0449 |  |  |  |  |
|  |  |  | (0.213) |  |  |  |  |
|  |  |  |  |  |  |  |  |
| DMT * Family Income |  |  |  | 3.149^**^ |  |  |  |
|  |  |  |  | (0.933) |  |  |  |
|  |  |  |  |  |  |  |  |
| Ayahuasca * Family Income |  |  |  |  | 0 |  |  |
|  |  |  |  |  | (.) |  |  |
|  |  |  |  |  |  |  |  |
| Peyote/Mescaline * Family Income |  |  |  |  |  | 0.701^*^ |  |
|  |  |  |  |  |  | (0.311) |  |
|  |  |  |  |  |  |  |  |
| LSD * Family Income |  |  |  |  |  |  | -0.550^*^ |
|  |  |  |  |  |  |  | (0.233) |
|  |  |  |  |  |  |  |  |
| Constant | 15.20^***^ | 15.33^***^ | 15.27^***^ | 15.30^***^ | 15.29^***^ | 15.40^***^ | 15.09^***^ |
|  | (0.901) | (0.920) | (0.916) | (0.920) | (0.919) | (0.922) | (0.908) |
| Observations | 5886 | 5886 | 5886 | 5886 | 5886 | 5886 | 5886 |
| *R*^2^ | 0.146 | 0.147 | 0.147 | 0.147 | 0.147 | 0.148 | 0.149 |
| Source: 2008-2019 National Survey of Drug Use and Health, N=458,372  Standard errors in parentheses  ^a^ Men serve as the references category  ^b^ Single Serves as the reference category for marital statuses  ^c^ 2008 serves as the reference category for the year survey was taken  ^†^ *p* < 0.1, ^*^ *p* < 0.05, ^**^ *p* < 0.01, ^***^ *p* < 0.001 | | | | | | | |
